# Glutathione-activatable synthetic channel for hopping-mediated anion transport

**DOI:** 10.1039/d6sc00359a

**Published:** 2026-02-17

**Authors:** Sandip Chattopadhayay, Debraj Ganguly, Triveni Sodnawar, Pinaki Talukdar

**Affiliations:** a Department of Chemistry, Indian Institute of Science Education and Research Pune Dr Homi Bhabha Road, Pashan Pune 411008 Maharashtra India ptalukdar@iiserpune.ac.in; b Department of Biotechnology, Savitribai Phule Pune University Pune 411007 Maharashtra India; c Department of Chemistry, School of Natural Sciences, Shiv Nadar Institution of Eminence Delhi NCR 201314 India

## Abstract

Controlled transport of ions across the cellular membrane is an essential process. While nature employs stimuli-gated transmembrane proteins to facilitate the appropriate transport of essential ions or molecules across cellular membranes, the endeavor to create a stimuli-controlled synthetic analogue presents considerable challenges. Herein, we introduced isophthalamide-based synthetic ion transporters 1a–1e and a protransporter 1c′. Transport studies divulged that even though the protransporter 1c′ cannot transport the anions, the glutathione-based activation generates a self-assembled anion channel 1c in the membrane and turns ON the anion transport with preferential selectivity towards the chloride anion. Detailed mechanistic studies validated that the transport of anions occurs *via* the antiport mechanism. An electrophysiological experiment divulged that the protransporter is inefficient in forming stable channels in the membrane. In contrast, the addition of the GSH releases the compound 1c, which forms a stable channel in the membrane with an average diameter of 4.7 ± 0.3 Å. The calculated single-channel conductance is 165 ± 1 pS, and the average *P*_Cl^−^_/*P*_K^+^_ = 5.0 ± 1.3. A dodecameric assembly of the monomeric rosette of 1c and [(1c)_12_ + Cl^−^] was geometrically optimized to understand the ion channel formation and investigate the responsible channel–ion interactions for the ion transport process.

## Abstract 2

A glutathione-activatable synthetic anion channel is reported. The doubly 2,4-dinitrobenzene-protected form lacks ion channel activity due to poor self-assembly. Activation with glutathione restores self-assembly and turns ON ion channel activity.

## Graphical abstract

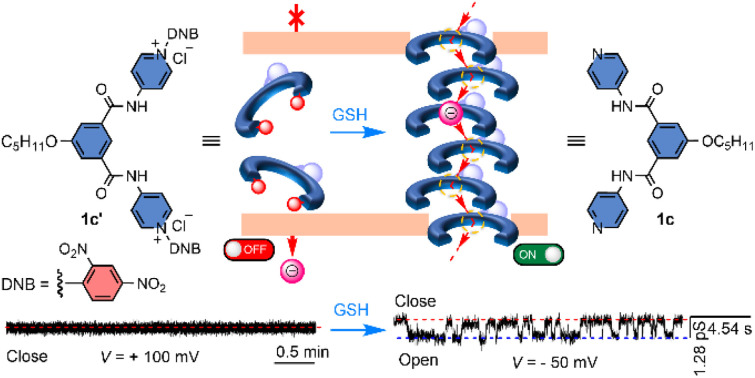

## 1 Introduction

The cellular membrane acts as a protective layer, preventing unwanted guests from entering while allowing the selective passage of ions or large polar molecules to maintain normal physiological functions.^1–3^ Membrane-embedded, structurally and functionally complex proteins, such as ion channels and pores, control ion translocation in response to specific stimuli. Importantly, malfunctions in these transmembrane proteins can result in serious diseases known as channelopathies.^4–6^ Consequently, the design of synthetic transporters offers promising prospects. These synthetic channel-forming molecules hold potential applications in elucidating transport mechanisms, advancing drug delivery systems, developing biosensors, and providing therapeutic solutions for various channel-related diseases.

Inspired by nature, different stimuli-gated systems have been developed in recent years to modulate the movement of the ions across the bilayer membrane. Different stimuli have been used to control the ion transport process across the membrane, including pH, light, ligands, voltage, enzymes, and GSH.^7–18^ With the parallel development of pH, light, enzyme, and ligand-gated synthetic transporters in recent years, designing a synthetic transport system in which the ion flux can be modulated with GSH also requires significant attention. GSH as a stimulus in synthetic ionophore systems creates hope for generating an alternative strategy to decorate different stimuli-gated synthetic ion transport systems, thereby opening up avenues for their utilization in various therapeutic applications.

The GSH-mediated transport activation concept was introduced by Manna and coworkers using synthetic transporters.^18^ A GSH-triggered activation was used in thiourea-based tripodal compounds to activate the Cl^−^ ion transport process. Gabbaï and coworkers introduced GSH as an external stimulus for controlling the Cl^−^ ion transport activity of antimony-based anion transporters by manipulating both the lipophilicity of the transporter and its overall anion affinity.^14^ Gale and associates invented gold complexes as efficient switchable anion transporters by complexing bisimidazole anion transporters with Au^III^, which blocks the anion binding site.^19^ A GSH-based decomplexation of gold initiates the H^+^/Cl^−^ cotransport by releasing the active transporter across the liposome. Recently, Manna and coworkers developed a thiourea-based procarrier linked with the Arg–Gly–Asp (RGD) peptide.^20^ The GSH-induced release of active transporter from the procarrier initiated H^+^/Cl^−^ cotransport. All of the above-mentioned examples are based on synthetic carrier systems. In the domain of artificial ion channels, our group developed the GSH-triggerable ion channel system for the transmembrane transport of cation–anion pairs.^12^ The rare example of a GSH-based activation in the synthetic ion channel continuously stimulates our intellectual pursuit to introduce different synthetic ion channels where GSH can trigger ion transport activity.

**Fig. 1:**
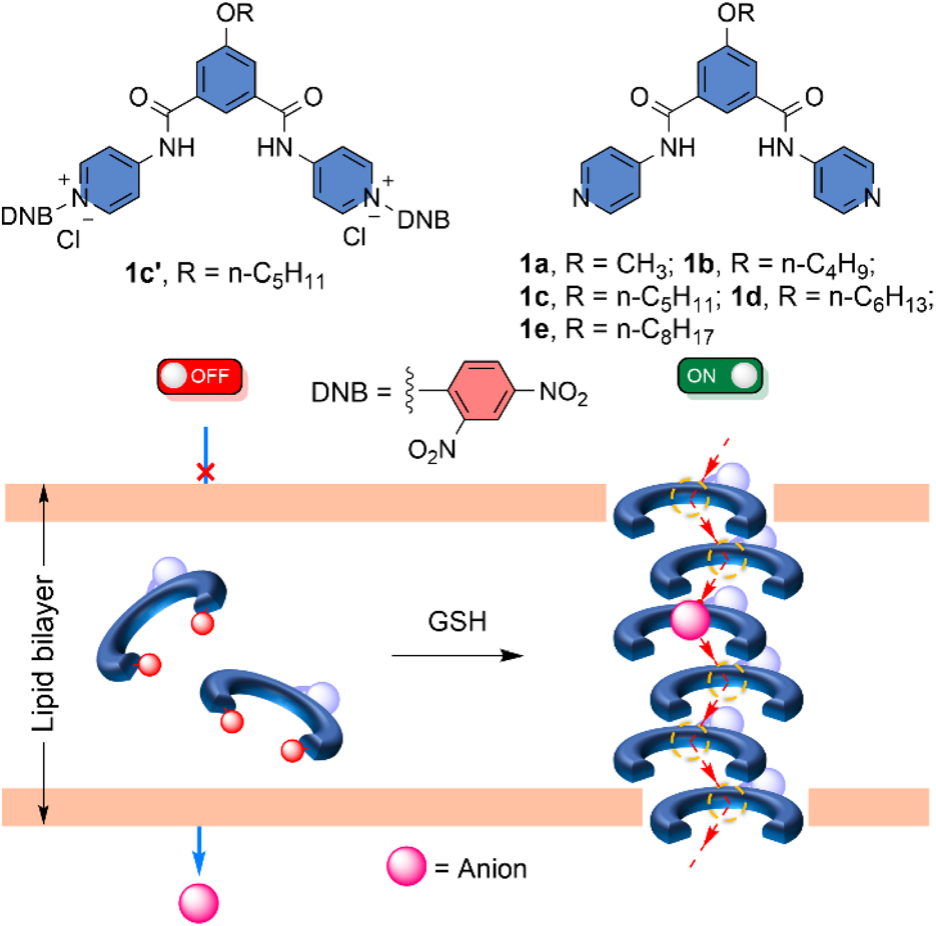
Chemical structure of compounds 1a–1e and 1c′, and graphical representation of the generation of barrel-rosette anion channel from the corresponding protransporter by using GSH as a stimulus.

Herein, we designed an isophthalamide-based self-assembled barrel rosette ion channel 1a–1e, which can transport the anion across the membrane (Fig. 1). Different synthetic analogues were used to modulate the membrane insertion efficiency by altering the lipophilicity of the channel-forming molecule. Moreover, to mask the ion transport activity, the pyridine groups of compound 1c were attached to the 2,4-dinitrochlorobenzene (DNCB) to generate the protransporter 1c′. The attachment of 2,4-dinitrobenzene (DNB) to the transport active channel molecule decreases membrane insertion efficiency by altering lipophilicity and disrupting the self-assembly process. Therefore, it is expected that no transport behaviour will be shown across the membrane. Notably, the addition of GSH can turn ON the transport process by releasing the channel-forming molecule in the membrane,^21^ which can self-assemble to form the barrel-rosette anion channel.

## 2 Results and discussion

### 2.1 Design and synthesis of 1a–1e and 1c′

**Scheme 1:**
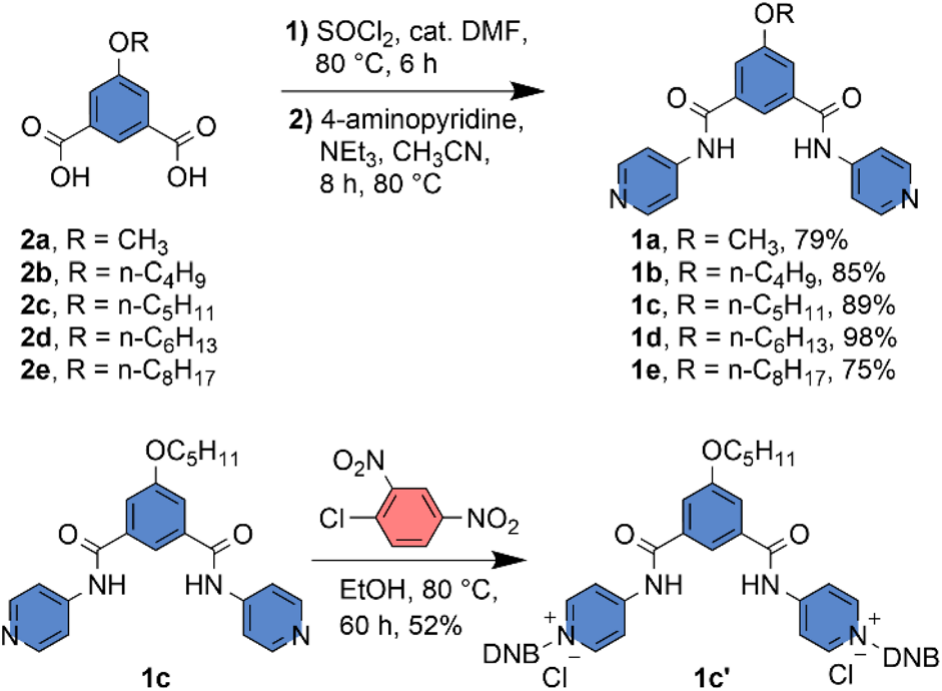
Synthetic scheme of compounds 1a–1e and 1c′.

Compounds 2a–2e were synthesized as described in the SI. Compounds 2a–2e were treated with SOCl_2_ with catalytic DMF at 80 °C to convert them to the corresponding acid chloride. The acid chloride was directly coupled with 4-aminopyridine at 80 °C with NEt_3_ as a base and CH_3_CN as a solvent to obtain the desired compounds 1a–1e with a significant yield (Scheme 1). The protransporter 1c′ was synthesized by reacting 1c with 2,4-dinitrochlorobenzene at 80 °C in EtOH solvent (Scheme 1). All compound characterization data are provided in the SI.

### 2.2 Self-assembly and morphological study

**Fig. 2:**
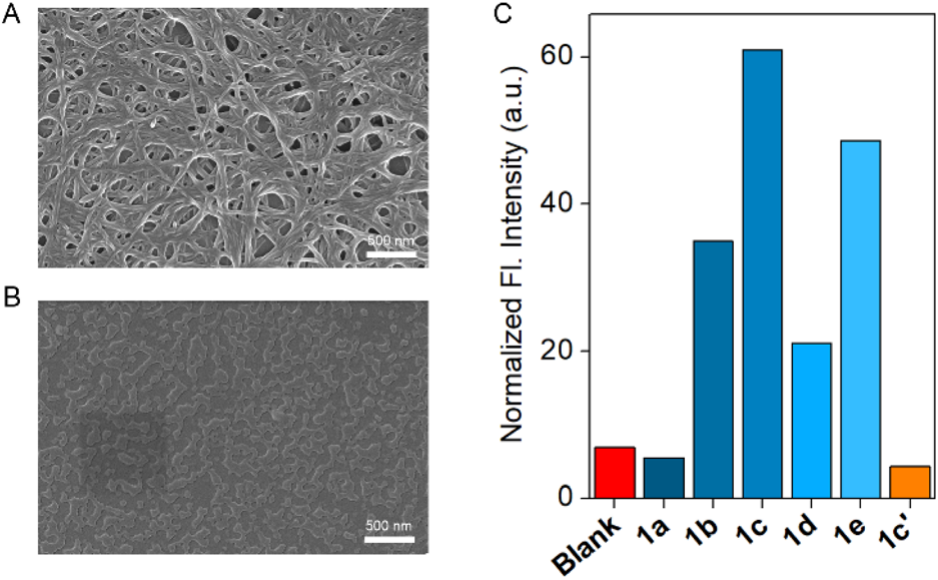
Morphological study of 1c (A) and 1c′ (B) in CH_3_CN solvent by using FESEM. Ion transport activity comparison of 1a–1e and 1c′ across EYPC-LUVs ⊃ HPTS (C).

The self-aggregation property of compound 1c was initially investigated with concentration-dependent ^1^H NMR in CDCl_3_ solvent at 25 °C. Increasing the concentration of compound 1c also increased the downfield shift of the H_a_ proton, indicating that at elevated concentrations, the compound is capable of forming intermolecular H-bonding (Fig. S2). Furthermore, the temperature-dependent ^1^H NMR experiment confirmed that increasing the temperature results in an upfield shift of the H_a_ proton (Fig. S3). This data revealed that an elevation in temperature breaks the intermolecular H-bonding, thereby upfielding the H_a_ proton. Furthermore, to understand the aggregation pattern of compound 1c, field-emission scanning electron microscopy (FESEM) was performed in CH_3_CN and MeOH solvents (Fig. S1). Irrespective of the solvent chosen, the compound forms a long helical fibril morphology, which indicates that compound 1c exhibits strong aggregation due to the formation of intermolecular H-bonding (Fig. 2A, S1A and C). On the contrary, compound 1c′ did not form any long helical fibril morphology in either CH_3_CN or MeOH solvent (Fig. S1B and D). In MeOH solvent, 1c′ formed small spherical structures with an average diameter of 0.39 µm. In the case of the CH_3_CN solvent, an insignificant assembly of the compound was noticed (Fig. 2B). This data indicated that even though compound 1c formed a strong aggregation, the corresponding protransporter 1c′ did not show any strong aggregation property.

### 2.3 Ion binding studies

The ion binding efficiencies of active transporter 1c and protransporter 1c′ were investigated by ^1^H NMR titration in acetonitrile-d_3_ with a host concentration of 2 mM.^22^ An increase in the equivalent of the tetrabutylammonium chloride (TBACl) salt with compound 1c showed a significant downfield shift of the H_a_, H_b_, and H_c_ protons (Fig. S4), confirming the involvement of these protons in the overall ion binding process through the N–H⋯Cl^−^ and two aromatic C–H⋯Cl^−^ hydrogen bonding interactions. Whereas, an insignificant downfield shift of compound 1c′ was noticed upon the addition of the TBACl salt (Fig. S13), confirming that compound 1c′ is inefficient in binding the Cl^−^ ion in the ion binding pocket. A significant change in the peak position during the titration with compound 1c and TBACl salt inspired us to investigate the ion binding process with the other TBAX (X^−^ = Br^−^, I^−^, NO_3_^−^, and ClO_4_^−^) salts (Fig. S6–S12). Titration data indicated that increasing the size of the anion decreases the downfield shift of the H_a_, H_b_, and H_c_ protons. In the case of TBAClO_4_, an insignificant change in the peak position was noticed, indicating that the larger size of the ClO_4_^−^ ion restricts it from binding in the ion binding pocket of compound 1c. The BindFit v0.5 program^23^ was further used to investigate the binding constant of compound 1c with TBAX salt. A 1 : 1 model of host:guest confirmed that compound 1c has the highest ion binding constant for Cl^−^ ion (26 055 M^–1^ ± 35%) followed by the Br^−^ (2301 M^–1^ ± 9%), NO_3_^−^ (91 M^–1^ ± 1%), and I^−^ (79 M^–1^ ± 2%) ions. An insignificant change in the peak positions during the titration of compound 1c with TBAClO_4_ and 1c′ with TBACl restricts us from investigating the corresponding ion binding constant. The efficiency of the Cl^−^ ion binding was also confirmed with HRMS spectra (Fig. S14).^24^ An equimolar mixing of 1c and TBACl provided the mass corresponding to the [1c + Cl^−^] complex, reconfirming the formation of 1 : 1 host : guest complex.

### 2.4 Transmembrane ion transport

A significant change in the ion binding constant of compound 1c with different TBAX salts led us to check its ion transport ability across the vesicular membrane. An egg yolk phosphatidylcholine large unilamellar vesicles (EYPC-LUVs), entrapped with a pH-sensitive 8-hydroxypyrene-1,3,6-trisulfonate (HPTS, p*K*_a_ = 7.2) dye,^25–27^ was used for evaluation of an initial assessment of the ion transport activity of compounds 1a–1e and 1c′. During the investigation of the transport studies, a 0.8 unit of pH gradient (pH_in_ = 7 and pH_out_ = 7.8) was created by the exogenous addition of 20 µL of the 0.5 M NaOH solution at *t* = 20 s. Then, the change in the HPTS fluorescence activity was monitored over time after the addition of the transporters at *t* = 100 s. Finally, Triton X-100 was added at *t* = 300 s to lyze the vesicles to achieve maximum fluorescence intensity of the HPTS dye. An initial transport activity screening of the compounds 1a–1e and 1c′ at 100 nM concentration showed a significant variation of the transport activity upon changing the substitution. Comparison data showed the activity sequence 1c > 1e > 1b > 1d > 1a ≈ 1c′ (Fig. 2C and S16). The marked difference between compound 1c and its corresponding protransporter 1c′ suggests the potential for their application as an OFF-to-ON transport system activated by specific stimuli. Further, a concentration-dependent ion transport activity was investigated for compounds 1b–1e (Fig. S17–S20). The insufficient transport activity of the 1a and 1c′ restricts us from investigating their concentration-dependent study. A Hill analysis of compounds 1b–1e validated that compound 1c has the lowest EC_50_ value (109.63 ± 9.92 nM, 0.16 mol%), followed by compounds 1e (188.96 ± 82.95 nM, 0.28 mol%), 1b (0.93 ± 0.1 µM, 1.38 mol%), and 1d (1.90 ± 2.99 µM, 2.81 mol%). This sequence also supported the earlier-mentioned activity comparison. The EC_50_ value of compounds 1a and 1c′ cannot be evaluated due to their insignificant transport activity. Analysis of all compounds revealed a Hill coefficient (*n*) ≈ 1, indicating the formation of a thermodynamically stable self-assembled ion channel across the bilayer membrane.

### 2.5 Ion selectivity studies

**Fig. 3:**
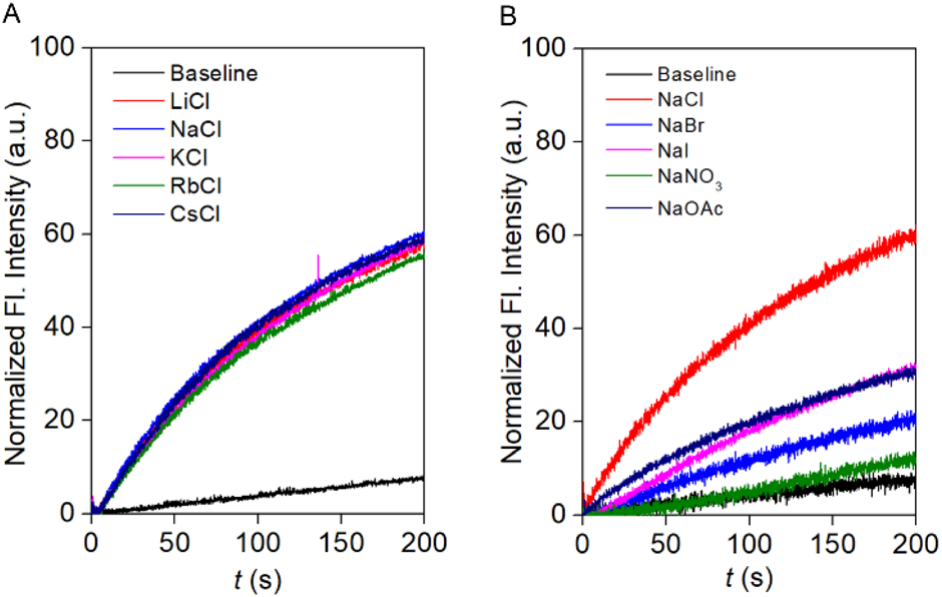
Cation (A) and anion (B) selectivity of compound 1c across EYPC-LUVs ⊃ HPTS at 100 nM concentration.

To investigate the ion selectivity of the designed compound, the most transport-active compound, 1c, was used. To elucidate the involvement of different ions in the transport process, firstly, the anion selectivity of compound 1c was investigated across the EYPC-LUVs ⊃ HPTS by changing the extravesicular NaX (X^−^ = Cl^−^, Br^−^, I^−^, NO_3_^−^, and OAc^−^) salts.^28,29^ A prominent variation in the ion transport activity confirmed the involvement of the anions in the overall transport process. The observed anion selectivity order is Cl^−^ > OAc^−^ ≈ I^−^ > Br^−^ > NO_3_^−^ (Fig. 3B). The experimentally obtained results did not follow any of the Hofmeister series, indicating that a combination of hydration energy and size of the anions governs anion selectivity (Fig. S21). To address the involvement of the cations in the ion transport process, the extravesicular MCl salts (M^+^ = Li^+^, Na^+^, K^+^, Rb^+^, and Cs^+^) were varied. An insignificant change in the ion transport activity was noticed during the alteration of the extravesicular cations (Fig. 3A). These data validated that cations are not involved in the overall transport process.

### 2.6 Chloride influx and cation selectivity studies

For assurance of the capability of the Cl^−^ ion transport by compound 1c, a halide-sensitive lucigenin dye was used.^30,31^ During the experiment, EYPC-LUVs were entrapped with 1 mM lucigenin and 200 mM NaNO_3_ at pH 7.0. The change in the lucigenin fluorescence activity was monitored in the presence of the compound 1c by creating a Cl^−^/NO_3_^−^ gradient. As expected, increasing the concentration of compound 1c increases the lucigenin fluorescence quenching (Fig. S23), indicating that compound 1c is capable of translocating the Cl^−^ ion across the membrane.

Furthermore, the involvement of cation selectivity was verified across the EYPC-LUVs system using lucigenin by varying the extravesicular MCl salt (M^+^ = Li^+^, Na^+^, K^+^, Rb^+^, and Cs^+^). As expected, an indistinguishable change in the lucigenin fluorescence activity was noticed (Fig. S24), confirming that compound 1c cannot transport the cations across the membrane. This cation selectivity also supports our earlier observation during the cation selectivity assay across the EYPC-LUVs ⊃ HPTS.

### 2.7 Mechanistic studies

**Fig. 4:**
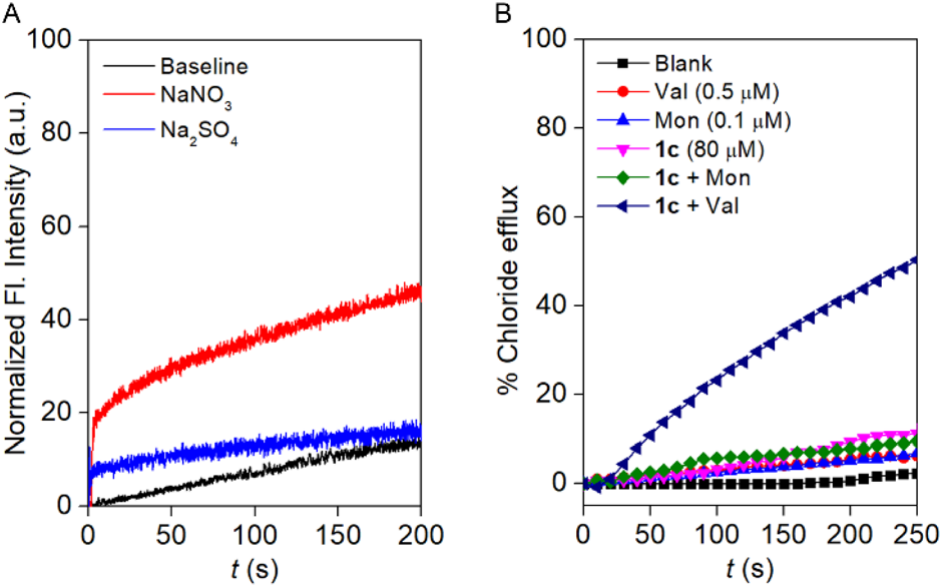
Cl^−^ efflux by compound 1c′ (2.5 µM) across EYPC-LUVs ⊃ lucigenin with intravesicular NaCl salt and iso-osmolar NaNO_3_ or Na_2_SO_4_ as extravesicular salt (A). Cl^−^ ion efflux by 1c in the absence and presence of monensin and valinomycin (B).

A prominent selectivity towards anions makes it crucial to understand the transport mechanism operated by compound 1c. Antiport (OH^−^/A^−^ or H^+^/M^+^, where A^−^ and M^+^ are anions and cations) or symport (M^+^/OH^−^, M^+^/A^−^ or H^+^/A^−^) of the ions can cause an increment in the HPTS fluorescence activity. The non-involvement of the metal ions rules out the possibility of symport of M^+^/OH^−^, M^+^/A^−^ ions, and antiport of H^+^/M^+^. Initially, the change in the Cl^−^ influx by compound 1c was monitored in the absence and presence of valinomycin (a K^+^ ion transporter).^32^ If compound 1c transports the ions *via* an antiport mechanism, it is expected to couple with the valinomycin and enhance the influx of the Cl^−^ ions in the presence of the valinomycin. On the contrary, in the case of the symport process, the Cl^−^ influx process is expected to remain unaltered in the presence of valinomycin. Interestingly, a significant enhancement in the lucigenin fluorescence quenching was observed in the presence of valinomycin (Fig. S26). This data preliminarily indicated that compound 1c follows the antiport as a dominant transport mechanism. Moreover, to confirm the transport mechanism, a NO_3_^−^/SO_4_^2−^ assay was carried out.^33^ During the assay, the EYPC-LUVs were entrapped with 1 mM lucigenin and 200 mM NaCl, and the pH was adjusted to 7.0. An extravesicular solution of iso-osmolar NaNO_3_ or Na_2_SO_4_ was used to create the ionic gradient, and fluorescence intensity was monitored after the addition of compound 1c. SO_4_^2−^ is a doubly negatively charged anion and, thereby, difficult to transport across the bilayer compared to the NO_3_^−^ ion. It is expected that if compound 1c follows the antiport process as the transport mechanism, then changing the extravesicular ion from NO_3_^−^ to SO_4_^2−^ will significantly reduce the fluorescence activity gain of the lucigenin dye. Notably, a significant difference in the fluorescence activity of the lucigenin dye was noticed upon variation of the extravesicular NaNO_3_ salt to Na_2_SO_4_ salt (Fig. 4A). This data confirmed that compound 1c predominantly follows the antiport as a transport mechanism.

Additionally, an ion-selective electrode (ISE) assay was conducted in the presence of valinomycin (an electrogenic K^+^ carrier) and monensin (an electroneutral H^+^/K^+^ antiporter) to unveil the ion transport mechanism. An insignificant enhancement in the Cl^−^ ion efflux was observed in the presence of monensin, confirming that electroneutral transport is not occurring by compound 1c. Whereas a significant enhancement in the Cl^−^ ion efflux was noticed in the presence of valinomycin, divulging that compound 1c followed mainly the electrogenic mode of ion transport (Fig. 4B). Subsequently, the effect of compound 1c on the membrane was investigated by 5(6)-carboxyfluorescein (CF) assay.^34^ CF is a self-quenching dye; therefore, the encapsulation of 50 mM of CF dye across the EYPC-LUVs quenches the fluorescence activity of the CF dye. In case of CF dye leakage, the fluorescent activity is expected to be enhanced due to the decrease in the concentration of the CF dye in the bulk solution. Interestingly, no significant enhancement in the CF fluorescence activity was observed upon the addition of compound 1c at different concentrations (Fig. S29). This data validated that compound 1c cannot form any pores across the membrane or disintegrate the bilayer membrane.

### 2.8 Mode of ion transport

The transport of ions can occur either through the carrier or channel mode across the membrane. Initially, a DPPC-based assay was conducted to understand the mode of the transport process.^35,36^ DPPC has a phase transition temperature of 41 °C. Hence, if the transporter behaves as a carrier, the corresponding ion transport activity is expected to decrease below 41 °C due to the difficulty in the shuttling movement of the carrier. Whereas, in the case of the channel, irrespective of whether it is above or below the transition temperature of the DPPC lipid, the transport activity is expected to show an insignificant change or remain unaltered. During the experiment, regardless of the temperature, no discernible change in transport activity was observed (Fig. S30). This data rules out the possibility of the carrier mechanism and provides preliminary support for channel formation by compound 1c in the membrane.

**Fig. 5:**
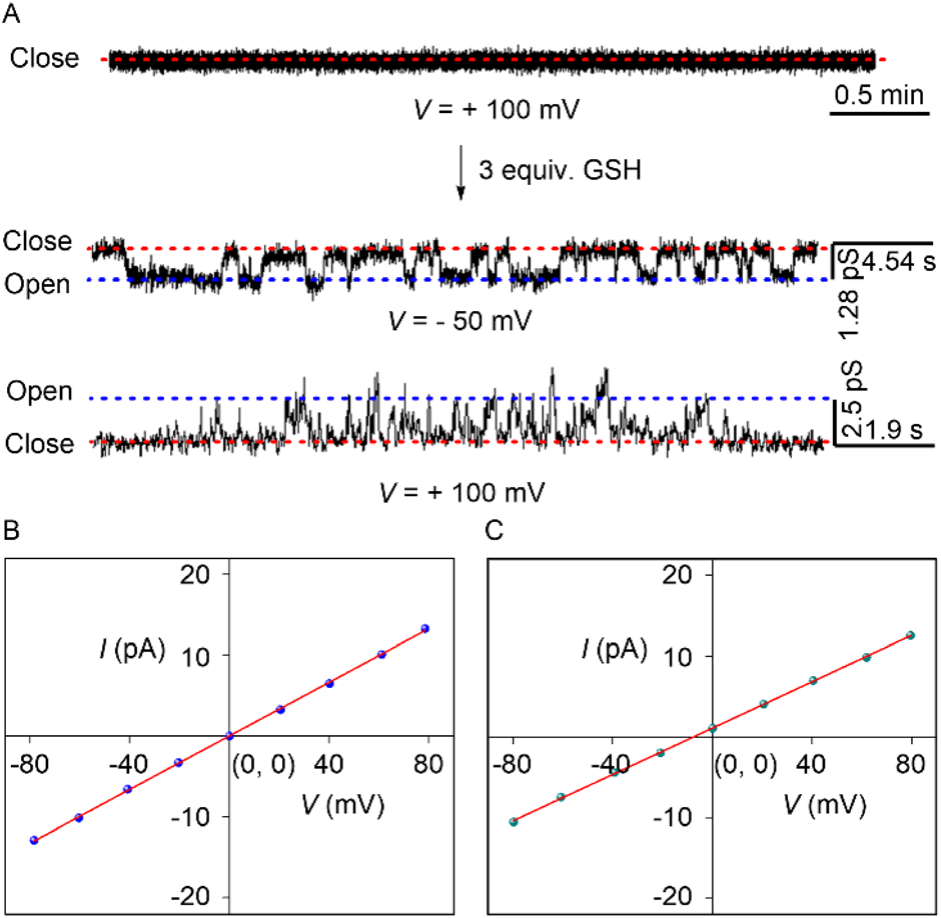
Opening-closing events of *in situ* generated channel 1c from protransporter 1c′ by GSH as stimuli (A). *I*–*V* plot of channel forming compound 1c with symmetric (*cis*/*trans* = 1 M/1 M KCl) (B) and unsymmetric (*cis*/*trans* = 1 M/2 M KCl) (C) unbuffered KCl solution.

Finally, the real-time channel formation by the compound 1c was investigated across the black lipid membrane (BLM).^33^ A bilayer membrane was formed by painting a solution of diphytanoyl phosphatidylcholine (diPhyPC) lipid in *n*-decane across the orifice of a polystyrene chamber. Both the *cis* and *trans* chambers were filled with an unbuffered 1 M KCl solution. The addition of the 1c (5 µM) in the *cis* chamber rapidly turns on the opening-closing events at +ve and −ve holding potentials. This data confirmed that compound 1c is capable of forming an ion channel in the membrane with an average diameter of 4.7 ± 0.3 Å. Further, the single-channel conductance of compound 1c was investigated with a symmetric 1 M unbuffered KCl solution (Fig. 5B and S32). A distinct linear increment in the current was noticed with an increase in the voltage. This data indicated that our developed channel obeyed the ohmic behavior and is non-dipolic. The average single channel conductance with compound 1c was 165 ± 1 pS.

Further, the anion/cation selectivity was determined using *cis*/*trans* = 1 M/2 M KCl salt. A distinct −ve reversible voltage was noticed with an average reversible voltage (*V*_r_) of −6.95 mV (Fig. 5C and S33). The reversible voltage was used further to evaluate the permeability ratio. The experimentally evaluated average permeability ratio *P*_Cl^−^_/*P*_K^+^_ = 5.0 ± 1.3, confirming that 1c exhibits 5 times higher permeability towards the Cl^−^ ion compared to the K^+^ ion.

### 2.9 GSH-triggered activation of ion transport

Before evaluating the GSH-based transport activation process, the efficient release of the active transporter 1c from the protransporter 1c′ was evaluated by the ^1^H NMR experiment. A 2 mM stock solution of compound 1c′ was prepared in the DMSO-*d*_6_ solvent in an NMR tube. The generation of the 1c was monitored by sequential addition of the GSH into the NMR tube. An instantaneous change in the peak positions of the H_a_′ → H_a_, H_b_′ → H_b_, H_c_′ → H_c_, and H_d_′ → H_d_ was noticed after the addition of 2 equivalents of GSH in the NMR tube (Fig. S34). This data indicated the successful conversion of the protransporter 1c′ to the active transporter 1c upon the addition of the GSH. Further HRMS was collected after reacting compound 1c′ with GSH to understand the successful formation of the side product GS-DNB. A distinct peak in the HRMS spectra verified the successful formation of the GS-DNB (Fig. S35) during the generation of 1c from 1c′. Although the cleavage of DNB is known to be more pronounced in the presence of GSH,^21^ we have also tested the cleavage of the DNB group using Na_2_S_2_O_4_ as a reducing agent. We observed insignificant changes in the ^1^H NMR spectrum after the addition of 2 equivalents of Na_2_S_2_O_4_ (Fig. S37), indicating that the cleavage of the DNB group is not susceptible to Na_2_S_2_O_4_.

Finally, GSH-based channel activation was carried out across the black lipid membrane (BLM). The addition of protransporter 1c′ showed no opening-closing events even after the addition of 2–3 h, indicating that inadequate self-assembly restricts it from forming a stable ion channel in the membrane. Whereas the addition of the 3 equivalents of GSH rapidly triggered the frequent opening-closing events at different holding potentials (Fig. 5A). The frequent opening-closing events are due to the *in situ* release of channel-forming compound 1c in the membrane. Therefore, this experimental data confirmed that 1c′ can be utilized as a GSH-triggered OFF-to-ON channel formation in the membrane.

### 2.10 Theoretical studies

**Fig. 6:**
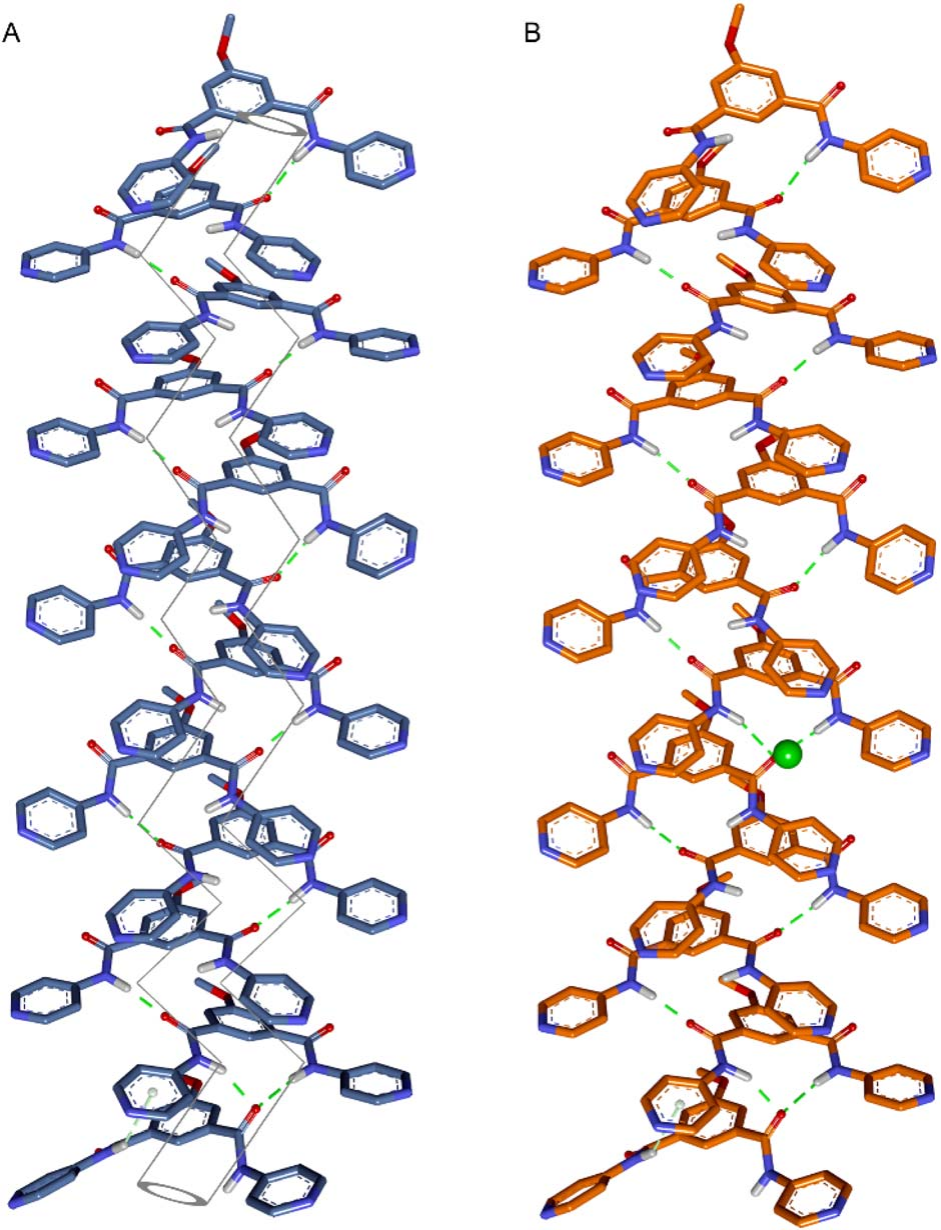
Geometry optimized structure of a dodecameric assembly of the monomeric rosette of 1c (A) and [(1c)_12_ + Cl^−^] (B) with an imaginary ion translocation pathway. Only polar hydrogens are shown for clarity of the structure.

Compound 1c was crystallized by slow evaporation of MeOH solvent. The crystal structure of compound 1c was used to understand the disassembled structure of protransporter 1c′ and find out the probable channel assembly formation by 1c in the membrane. Initially, the monomeric unit of the crystal structure of compound 1c was geometrically optimized with the Cl^−^ ion by using the Gaussian 09 program package^37,38^ using the B3LYP functional and 6–311++G(d,p) basis set. Geometry-optimized data validated that the H_a_, H_b_, and H_c_ protons are involved in Cl^−^ ion binding (Fig. S40), corroborating the experimentally obtained ^1^H NMR titration data. Based on the experimentally evaluated Hill coefficient value, a monomeric rosette of a dodecameric assembly of compound 1c was generated by taking the monomeric layer of the crystal structure of compound 1c. The pentyl chain was replaced with a methyl chain to reduce the computation time. Subsequently, the assembly was optimized using MOPAC software^39^ with the PM6-DH+ method (Fig. 6A and S40).^40^ The average calculated diameter is 3.6 ± 0.2 Å. The calculated diameter is in agreement with the experimentally evaluated channel diameter from the electrophysiological experiment. Furthermore, to understand the probable interaction of the Cl^−^ ion during passage through the ion translocation pathway, the Cl^−^ ion was placed at different positions within the monomeric rosette of the dodecameric assembly of the channel. Optimized results revealed that the Cl^−^ ion has hydrogen bonding interactions with the N–H and aromatic C–H protons (Fig. 6B, S41–S43). Therefore, these overall ion and channel interactions facilitate the passage of ions through the ion translocation pathway. Moreover, to understand the disassembly process of protransporter 1c′, geometry optimization was carried out with the Gaussian 09 program package using the B3LYP functional and 6–311++G(d,p) basis set. Optimized data indicated that the 2,4-dinitrobenzene unit remains perpendicular to the isophthalamide unit (Fig. S39). Hence, the structural orientation of the molecule probably restricts it from forming a strong self-assembly *via* the formation of intermolecular H-bonding and π–π stacking interactions. This geometry optimizes data also indicated that one of the Cl^−^ ions of compound 1c′ preferably stays in its anion binding pocket. This data explains why compound 1c′ shows an insignificant Cl^−^ ion binding in the ^1^H NMR titration experiment.

## 3 Conclusions

We developed a glutathione (GSH)-activated synthetic anion channel system. A series of molecules was synthesized to modulate ion transport activity by varying the lipophilicity of the compounds. Among these, compound 1c demonstrated superior ion transport efficacy. However, the corresponding protransporter 1c′ could not mediate ion transport due to inadequate self-assembly. Comprehensive transport studies revealed that compound 1c exhibited anion selectivity, with a particular preference for Cl^−^ ions, and followed an antiport mechanism. Electrophysiological experiments confirmed the formation of ion channels by compound 1c within the membrane with an average channel diameter of 4.7 ± 0.3 Å and single-channel conductance of 165 ± 1 pS. Additionally, the selectivity assay demonstrated that the ion channel exhibited five-fold greater permeability towards Cl^−^ ions compared to K^+^ ions. A GSH-mediated activation of protransporter 1c′ initiated transport activity by releasing the channel-forming molecule 1c within the membrane. Theoretical studies confirmed that intramolecular hydrogen bonding and π–π stacking interactions stabilize the channel assembly. Further analysis revealed that the N–H and aromatic C–H groups of the assembled channel interact with the chloride ion, and these specific channel–ion interactions cooperatively facilitate anion transport through the ion translocation pathway.

## Author contributions

P. T. conceived and directed the project. S. C. carried out all of the experiments. D. G. synthesised and characterised the compounds. T. S. carried out the NMR titration experiments. S. C. and P. T. wrote the manuscript. All authors approved the final version of the manuscript.

## Conflicts of interest

There are no conflicts to declare.

## Supplementary Material

SC-017-D6SC00359A-s001

SC-017-D6SC00359A-s002

## Data Availability

CCDC 2454559 (1c) contains the supplementary crystallographic data for this paper.^41^ The datasets supporting this article have been uploaded as part of the supplementary information (SI). Supplementary information: data for this paper, including synthesis, compound characterization, experimental procedures, and theoretical calculations. See DOI: https://doi.org/10.1039/d6sc00359a.
