# Supplementary material for: Glutathione-activatable synthetic channel for hopping-mediated anion transport
Source: Chem Sci. 2026 Feb 17;17(14):6995–7001. doi: 10.1039/d6sc00359a (PMC12911877; doi:10.1039/d6sc00359a)
Supplement: SC-017-D6SC00359A-s001 [file SC-017-D6SC00359A-s001.pdf]

Supplementary Information (SI) for Chemical Science.
This journal is © The Royal Society of Chemistry 2026

Supporting Information

Glutathione-activatable synthetic channel for hopping-mediated anion transport

Sandip Chattopadhayay,[a] Debraj Ganguly,[a] Triveni Sodnawar,[b] and Pinaki Talukdar*[a,c]

[a] Department of Chemistry, Indian Institute of Science Education and Research Pune, Dr. Homi Bhabha Road, Pashan, Pune 411008, Maharashtra, India

[b] Department of Biotechnology, Savitribai Phule Pune University, Pune 411007, Maharashtra, India

[c] Department of Chemistry, School of Natural Sciences, Shiv Nadar Institution of Eminence, Delhi NCR 201314, India

Table of Contents

**I.**	**General Methods**	**S2**
**II.**	**Physical Measurements**	**S2**
**III.**	**Synthesis**	**S3 – S6**
**IV.**	**Morphological Study**	**S6**
**V.**	**Concentration Dependent ^{1}H NMR Studies**	**S6 – S7**
**VI.**	**Temperature Dependent ^{1}H NMR Studies**	**S7 – S8**
**VII.**	**Ion Binding Studies**	**S8 – S13**
**VIII.**	**Mass Spectrometric Studies for Anion Recognition**	**S14**
**IX.**	**Ion Transport Studies**	**S14 − S25**
**X.**	**Planar Bilayer Conductance Measurements**	**S25 − S29**
**XI.**	**GSH-based Release of the Transport Active Ion Channel**	**S29 – S31**
**XII.**	**Single Crystal X-Ray Diffraction Study**	**S32 – S40**
**XIII.**	**Theoretical Studies**	**S40 – S45**
**XIV.**	**NMR Data**	**S46 − S57**
**XV.**	**References**	**S58**

I. General methods:

Reagents and compounds used for the synthesis were purchased from Sigma-Aldrich, Avra Synthesis Pvt. Ltd., and Spectrochem and used without further purification. For chemical reactions, MeOH, DMF, CH_3CN, EtOH, $CHCl_3$, and Pet ether were purchased from commercial suppliers and used without further purification. All reactions were performed under a nitrogen atmosphere using an N_2 gas balloon and monitored by TLC, performed on pre-coated aluminum plates of silica gel 60 F_{254} (0.25 mm, E. Merck). Column chromatographies were performed on Merck silica gel (100–200 mesh). Egg yolk phosphatidylcholine (EYPC) was obtained from Avanti Polar Lipids as a solution in $CHCl_3$ (25 mg/mL). HEPES buffer, HPTS, Lucigenin, Triton X–100, NaOH, and all inorganic salts were purchased in molecular biology grade from Sigma. Large unilamellar vesicles (LUVs) were prepared by using a mini extruder, equipped with a polycarbonate membrane of 100 nm or 200 nm pore size, purchased from Avanti Polar Lipids.

II. Physical measurements:

The ^{1}H NMR spectra were recorded at 400 MHz, ^{19}F spectra at 377 MHz, and ^{13}C spectra at 101 MHz using a Bruker NMR instrument. The residual solvent signals (deuterium) were considered as an internal reference (δH = 7.26 ppm for $CDCl_3$, δH = 3.31 ppm for MeOH-d_4, δH = 2.50 ppm for DMSO-d_6) to calibrate the spectra. All chemical shifts were reported in parts per million (ppm). The following abbreviations were used to indicate multiplicity patterns: s: singlet, d: doublet, t: triplet, q: quartet, p: pentet, h: hexet, m: multiplet, dd: doublet of doublets, ddd: doublet of doublet of doublets, dt: doublet of triplets, td: triplet of doublets. Coupling constants were measured in Hz. High-resolution mass spectra (HRMS) were recorded on electrospray ionization time-of-flight (ESI–TOF) with +ve or −ve mode. The pH of buffer solutions was adjusted using a Hanna HI98108 pHep$^+$ pH meter. ChemBio Draw 21.0.0 software was used to draw structures and process the Figures. All buffer solutions were prepared from autoclaved water. Fluorescence experiments were recorded using a Fluoromax-4 and Fluoromax-4+ from Jobin Yvon Edison, equipped with an injector port and a magnetic stirrer in a microfluorescence cuvette. The extravesicular dye was removed by performing gel chromatography using Sephadex G-50. The fluorescence data were processed using Origin 8.5 software. Conductance measurement through an ion channel was carried out in a planar bilayer lipid membrane (BLM) workstation obtained from Warner Instrument, consisting of a head-stage and its corresponding amplifier BC-535, an 8-pole Bessel filter LPF-8, Axon CNS Digidata 1440A, and pClamp 10 software. The conductance data were analyzed using Clampfit 10.6 software.

III. Synthesis:

A. Synthesis of active transporters:

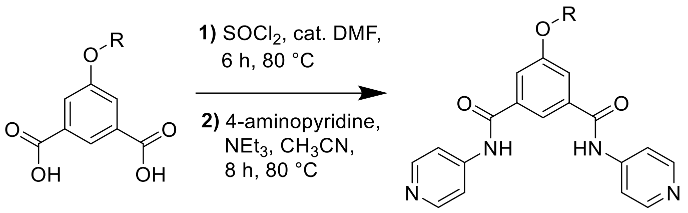

2a, R = CH_3
2b, R = n-C_4H_9
2c, R = n-C_5H_{11}
2d, R = n-C_6H_{13}
2e, R = n-C_8H_{17}

1a, R = CH_3, 79%
1b, R = n-C_4H_9, 85%
1c, R = n-C_5H_{11}, 89%
1d, R = n-C_6H_{13}, 98%
1e, R = n-C_8H_{17}, 75%

Scheme S1: Synthetic scheme for the compounds **1a**–**1e**.

The general synthetic procedure of compound 2a–2e: Compound **2a**–**2e** were synthesized by following the reported literature procedure.[S1–S4] All compounds were directly used for the next step without further purification.

The general synthetic procedure of compound 1a–1e: In a clean and dry 50 mL round-bottomed flask, compound **2a**–**2e** (1 equiv.) was dissolved in $SOCl_2$ (6 mL), and a catalytic amount of DMF (1–2 drops) was added to it. The reaction mixture was kept at 80 °C for 6 h. After the formation of the corresponding acid chloride of compound **2a**–**2e**, additional $SOCl_2$ was removed under reduced pressure, and the mixture was kept in a high vacuum for 1 h to remove the trace amount of $SOCl_2$ from it. The acid chloride was used directly in the subsequent step reaction without further purification.

In another clean and dry 50 mL double-neck round-bottomed flask, the required amount of 4-aminopyridine (2 equiv.) was dissolved in a dry 10 mL CH_3CN solvent, and DIPEA (5 equiv.) was added. The reaction mixture was stirred for 15–20 min at room temperature. The corresponding acid chloride of compound **2a**–**2e** (dissolved in 4 mL of CH_3CN) was added dropwise into the reaction mixture, which was further kept at 80 °C for 6 h. After the reaction was completed, CH_3CN and DIPEA were evaporated using a rotary evaporator. It was further transferred into a separating funnel with EtOAc (25 mL) and washed with $NaHCO_3$ (25 mL). The aqueous layer was extracted with EtOAc (3 × 25 mL), and then the combined organic layer was washed with brine solution (25 mL). Finally, the organic layer was dried over Na_2SO_4, and the solvent was evaporated under reduced pressure. Finally, the crude product was purified through 100–200 mesh silica column chromatography to obtain pure compounds **1a**–**1e**.

Synthesis of 5-methoxy-N^1,N^3-di(pyridin-4-yl)isophthalamide (1a): By taking 500 mg of compound **2a**, compound **1a** was synthesized. The crude product was purified through 100–200 mesh silica column chromatography using methanol/chloroform eluent (12% MeOH/$CHCl_3$), yielding **1a** (693 mg, 79%). **^{1}H NMR (400 MHz, 1:10 (v/v) MeOH-d_4:$CDCl_3$):** δ 9.68 (s, 2H), 8.43 (d, J = 5.7 Hz, 4H), 7.96 (t, J = 1.5 Hz, 1H), 7.69 (dd, 4H), 7.61 (d, J = 1.4 Hz, 2H), 3.89 (s, 3H). **^{13}C NMR (101 MHz, 1:10 (v/v) MeOH-d_4:$CDCl_3$):** δ 166.29, 160.17, 149.91, 146.21, 135.83, 118.14, 117.18, 114.45, 55.80. **HRMS (ESI) m/z:** $[M+H]^+$ Calcd. for $C_{19}H_{16}N_4O_3H^+$: 349.1296, Found 349.1291. **IR (Neat, *ν*/cm^{-1})**: 3442, 3093, 2921, 2851, 2361, 1676, 1595, 1518, 1459, 1420, 1334, 1298, 1246, 1117, 1054, 1004, 827, 741, 664.

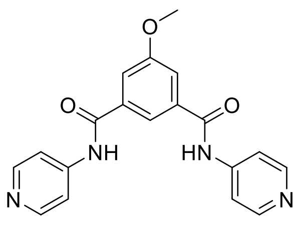

Synthesis of 5-butoxy-N^1,N^3-di(pyridin-4-yl)isophthalamide (1b): By taking 500 mg of compound **2b**, compound **1b** was synthesized. The crude product was purified through 100–200 mesh silica column chromatography using methanol/chloroform eluent (8% MeOH/$CHCl_3$), yielding **1b** (735 mg, 85%). **^{1}H NMR (400 MHz, DMSO-d_6):** δ 10.72 (s, 2H), 8.51 (d, J = 3.5 Hz, 4H), 8.13 (t, J = 1.5 Hz, 1H), 7.80 (d, J = 6.5 Hz, 4H), 7.73 (d, J = 1.4 Hz, 2H), 4.16 (t, J = 6.4 Hz, 2H), 1.77 (p, J = 6.5 Hz, 2H), 1.49 (dt, J = 14.6, 7.4 Hz, 2H), 0.97 (t, J = 7.4 Hz, 3H).**^{13}C NMR (101 MHz, DMSO-d_6):** δ 165.57, 158.70, 150.38, 145.75, 135.86, 119.53, 117.14, 114.13, 67.95, 30.66, 18.74, 13.71. **HRMS (ESI) m/z:** $[M+H]^+$ Calcd. for $C_{22}H_{23}N_4O_3H^+$ 391.1765, Found 391.1766. **IR (Neat, *ν*/cm^{-1}):** 3742, 3707, 3317, 3191, 2959, 2941, 2927, 2873, 1684, 1669, 1588, 1511, 1451, 1417 ,1331, 1309, 1292, 1232, 1211, 1117, 1067, 1027, 1007, 959, 882, 828, 750, 686.

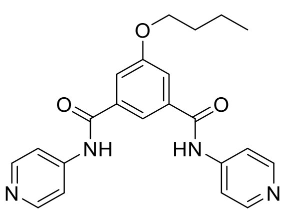

Synthesis of 5-pentoxy-N^1,N^3-di(pyridin-4-yl)isophthalamide (1c): By taking 500 mg of compound **2c**, compound **1c** was synthesized. The crude product was purified through 100–200 mesh silica column chromatography using methanol/chloroform eluent (6% MeOH/$CHCl_3$), yielding **1c** (717 mg, 89%). **^{1}H NMR (400 MHz, DMSO-d_6):** δ 10.71 (s, 2H), 8.50 (dd, 4H), 8.13 (d, J = 1.6 Hz, 1H), 7.80 (dd, 4H), 7.73 (d, J = 1.5 Hz, 2H), 4.15 (t, J = 6.4 Hz, 2H), 1.79 (p, J = 6.4 Hz, 2H), 1.46 (dt, J = 13.7, 6.3 Hz, 2H), 1.37 (dt, J = 14.9, 7.2 Hz, 2H), 0.92 (t, J = 7.1 Hz, 3H). **^{13}C NMR (101 MHz, DMSO-d_6):** δ 165.58, 158.70, 150.40, 145.75, 135.87, 119.54, 117.13, 114.13, 68.24, 28.30, 27.71, 21.90, 13.95. **HRMS (ESI) m/z:** $[M+H]^+$ Calcd. for $C_{23}H_{24}N_4O_3H^+$ 405.1922, Found 405.1922. **IR (Neat, ν/cm^{-1}):** 3901, 3422, 3307, 2951, 1683, 1650, 1591, 1511, 1439, 1418, 1347, 1333, 1312, 1289, 1211, 1117, 1057, 1046, 1001, 947, 883, 825, 735, 677, 626.

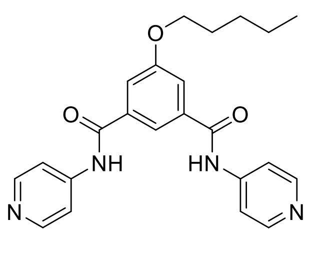

Synthesis of 5-(hexyloxy)-N^1,N^3-di(pyridin-4-yl)isophthalamide (1d): By taking 500 mg of compound **2d**, compound **1d** was synthesized. The crude product was purified through 100–200 mesh silica column chromatography using methanol/chloroform eluent (5% MeOH/$CHCl_3$), yielding **1d** (768 mg, 98%). **^{1}H NMR (400 MHz, 1:10 (v/v) MeOH-d_4:$CDCl_3$):** δ 9.45 (s, 2H), 8.47 (d, J

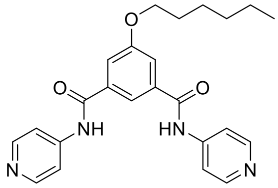

= 5.3 Hz, 4H), 7.93 (t, *J* = 1.5 Hz, 1H), 7.69 (dd, 4H), 7.61 (d, *J* = 1.4 Hz, 2H), 4.06 (t, *J* = 6.5 Hz, 2H), 1.80 (p, 2H), 1.46 (p, *J* = 14.8, 7.0 Hz, 2H), 1.33 (h, *J* = 7.3, 3.6 Hz, 4H), 0.90 (t, 3H). **^{13}C NMR (101 MHz, 1:10 (v/v) MeOH-*d*$_4$:CDCl$_3$):** δ 166.28, 159.74, 149.99, 146.12, 135.77, 117.84, 117.63, 114.40, 68.79, 31.45, 29.00, 25.58, 22.53, 13.93. **HRMS (ESI) m/z:** $[M+H]^+$ Calcd. for $C_{24}H_{26}N_4O_3H^+$ 419.2078, Found 419.2077. **IR (Neat, *ν*/cm^{-1}):** 3309, 2954, 2928, 2871, 2855, 1676, 1588, 1510, 1440, 1417, 1331, 1310, 1291, 1231, 1210, 1115, 1031, 1002, 942, 878, 825, 725, 667.

Synthesis of 1e 5-(octyloxy)-*N*^{1},*N*^{3}-di(pyridin-4-yl)isophthalamide (1e): By taking 500 mg of compound **2c**, compound **1c** was synthesized. The crude product was purified through 100–200 mesh silica column chromatography using methanol/chloroform as eluent (4% MeOH/$CHCl_3$), yielding **1c** (570 mg, 75%). **^{1}H NMR (400 MHz, 1:10 (v/v) MeOH-*d*$_4$:CDCl$_3$):** δ 9.98 (s, 2H), 8.35 (d, *J* = 5.7 Hz, 4H), 7.90 (t, *J* = 1.5 Hz, 1H), 7.68 – 7.65 (m, 4H), 7.57 (d, *J* = 1.4 Hz, 2H), 3.99 (t, *J* = 6.5 Hz, 2H), 1.72 (p, *J* = 6.7 Hz, 2H), 1.37 (q, *J* = 7.3 Hz, 2H), 1.30 – 1.20 (m, 4H), 1.20 – 1.14 (m, 4H), 0.81 – 0.74 (m, 3H). **^{13}C NMR (101 MHz, 1:10 (v/v) MeOH-*d*$_4$:CDCl$_3$):** δ 166.53, 159.71, 149.76, 146.41, 135.80, 118.02, 117.65, 114.52, 68.73, 31.71, 29.22, 29.15, 29.02, 25.88, 22.55, 13.91. **HRMS (ESI) m/z:** $[M+H]^+$ Calcd. for $C_{26}H_{30}N_3O_4H^+$ 447.2391, Found 447.2390. **IR (Neat, *ν*/cm^{-1}):** 3236, 3164, 3084, 2954, 2925, 2855, 2361, 2336, 1723, 1682, 1587, 1510, 1464, 1436, 1416, 1334, 1309, 1292, 1229, 1209, 1185, 1110, 1041, 1001, 944, 889, 861, 829, 739, 723, 677.

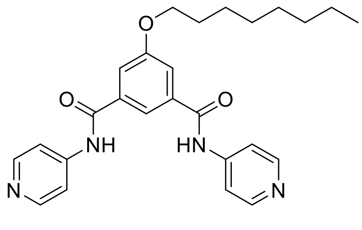

B. Synthesis of protransporter:

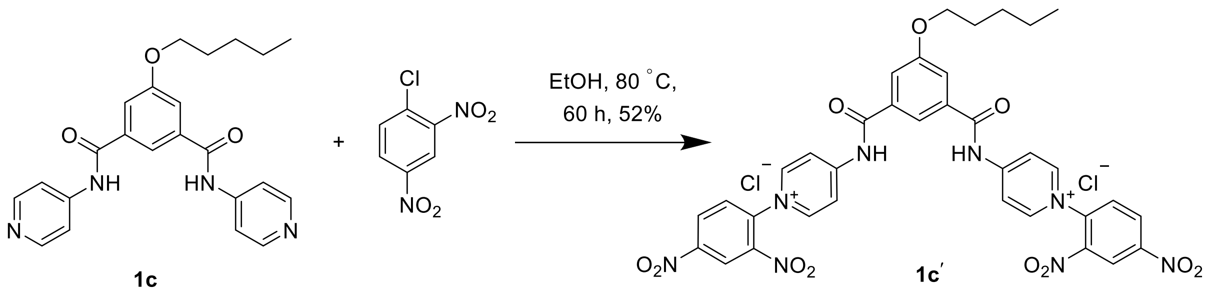

Scheme S2: Synthetic scheme for the protransporter **1c'**.

Compound **1c** (50 mg, 0.124 mmol, 1 equiv.) was taken in a clean and dry 10 mL round-bottom flask and dissolved in 4 mL of ethanol. 2,4-Dinitrochlorobenzene (150 mg, 0.742 mmol, 6 equiv.) was added, and the reaction mixture was heated at 80 °C for 60 h. After the reaction was completed, the reaction mixture was filtered by washing with ice-cold ethanol, and the precipitate was dried in a desiccator, yielding **1c'** (52 mg, 52%). **^{1}H NMR (400 MHz, DMSO-*d*$_6$):** δ 12.47 (s, 2H), 9.14 (d, *J* = 7.0 Hz, 4H), 9.12 (d, *J* = 2.6 Hz, 2H), 8.96 (dd, *J* = 8.7, 2.6 Hz, 2H), 8.70 (d, *J* = 6.9 Hz, 4H), 8.62 (s, 1H), 8.40 (d, *J* = 8.7 Hz, 2H), 7.98 (s, 2H), 4.25 (t, *J* = 6.4 Hz, 2H), 1.81 (q, *J* = 6.8 Hz, 2H), 1.50 – 1.45 (m, 2H), 1.43 – 1.38 (m, 2H), 0.93 (t, *J* = 7.1 Hz, 3H). **^{13}C NMR (101 MHz, DMSO-*d*$_6$):** δ 166.44, 159.07,

154.72, 148.88, 146.12, 143.40, 138.63, 134.05, 132.03, 130.16, 121.58, 121.20, 119.33, 115.29, 68.64, 28.24, 27.67, 21.88, 13.96. **HRMS (ESI) m/z:** $[M]^{2+}$ Calcd. for $C_{35}H_{30}N_8O_{11}^{2+}$ 369.1012, Found 369.1015. **IR (Neat, *ν*/cm^{-1}):** 3937, 3907, 3821, 3744, 3405, 3108, 2928, 1701, 1640, 1609, 1588, 1543, 1518, 1482, 1465, 1343, 1287, 1224, 1198, 1104, 1076, 1032, 836, 743, 700, 633, 614.

IV. Morphological Study:

Field emission scanning electron microscopy (FESEM) was used to understand the morphological pattern of protansporter **1c'** and active ion channel **1c**. A 1 mM stock solution of either **1c'** or **1c** was prepared in HPLC grade CH_3CN and MeOH solvents, and the solution was drop-casted on a silicon wafer, dried, and then used for FESEM studies.

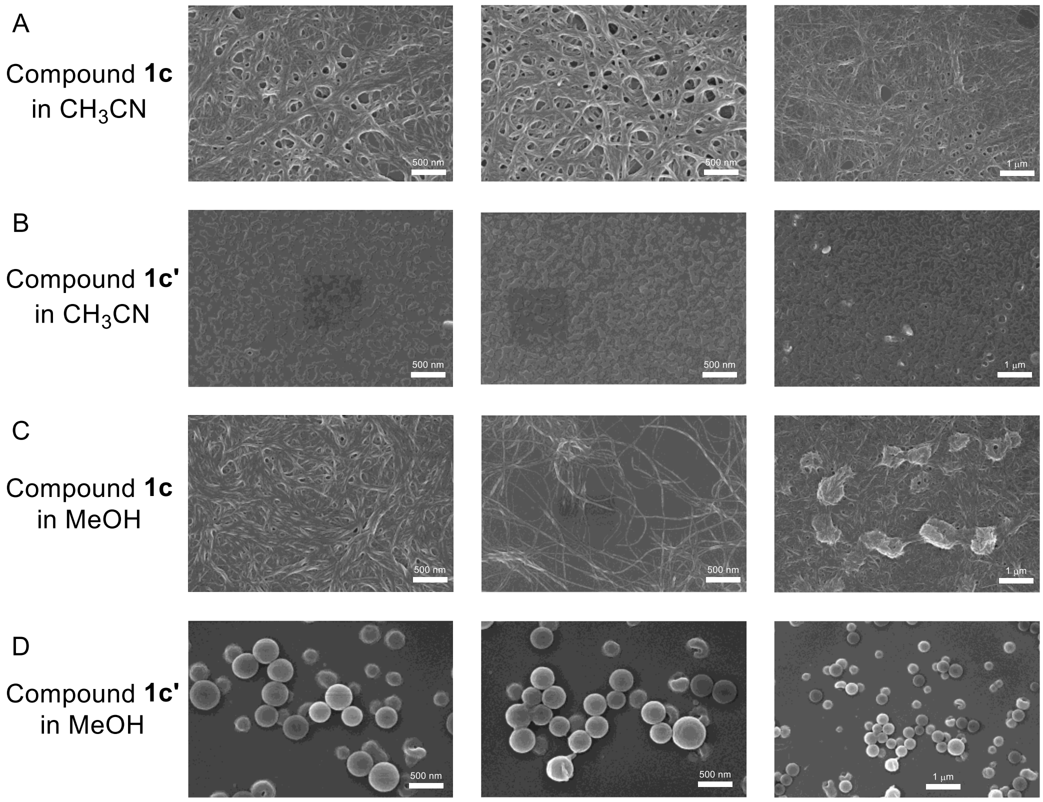

Fig. S1 FESEM images of **1c** (A, B), and **1c'** in CH_3CN (C) and MeOH (D) solvents.

V. Concentration Dependent ^{1}H NMR Studies:

To investigate the self-assembly property of compound **1c**, a concentration-dependent ^{1}H NMR was taken in $CDCl_3$ solvent at 25 °C. If compound **1c** forms an ion channel through self-assembly via π-π stacking, H-bonding, and other interactions, then some of the proton chemical shift positions are likely to shift with an increase in the concentration of the compound.

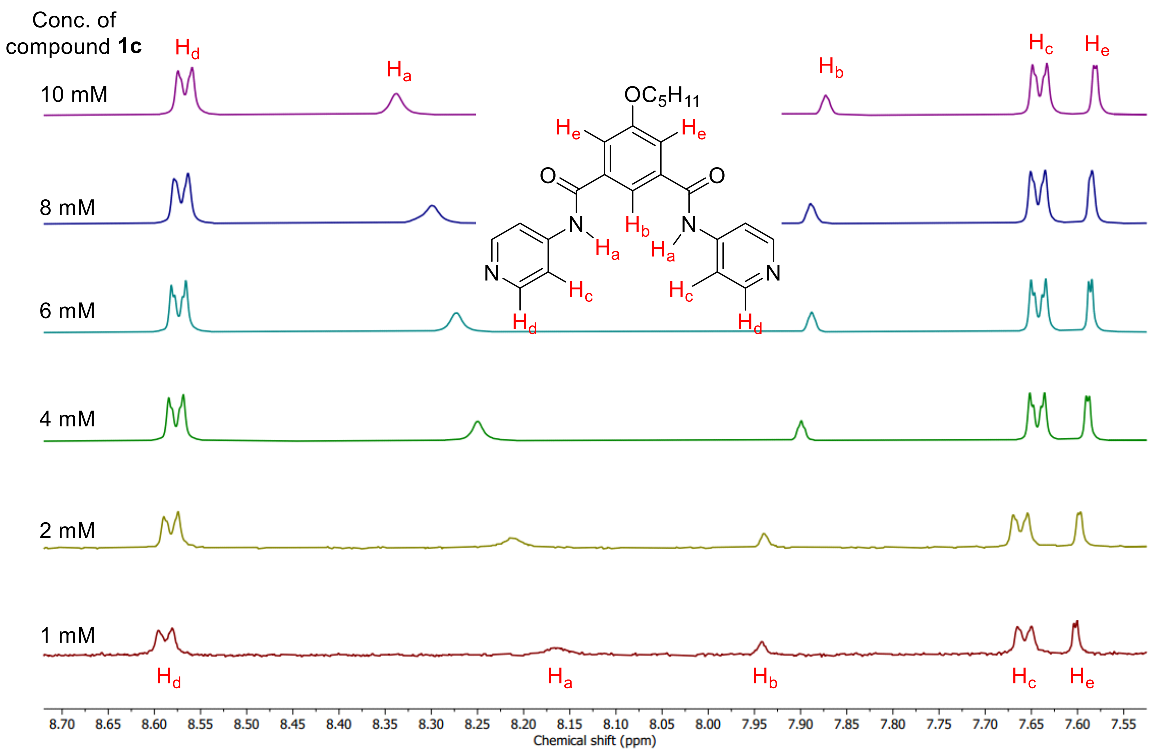

Fig. S2 Concentration-dependent ^{1}H NMR of channel forming compound **1c** in $CDCl_3$ solvent at 25 °C.

During the experiment, a prominent downfield shift of the H_a proton was noticed along with the upfield shift of H_b, H_c, H_d, and H_e protons, validating that compound **1c** underwent self-aggregation by forming both H-bonding interactions as well as via π-π stacking interactions.

VI. Temperature Dependent ^{1}H NMR Studies:

To ascertain whether the observed peak shift arises from intermolecular hydrogen-bond formation, temperature-dependent ^{1}H NMR studies were performed on compound **1c** (10 mM) in DMSO-d_6. A distinct, gradual upfield shift of the H_a protons was observed with an increase in temperature, validating the breakage of intermolecular H-bonding at elevated temperatures. This data also supports the self-aggregation process of compound **1c** at room temperature.

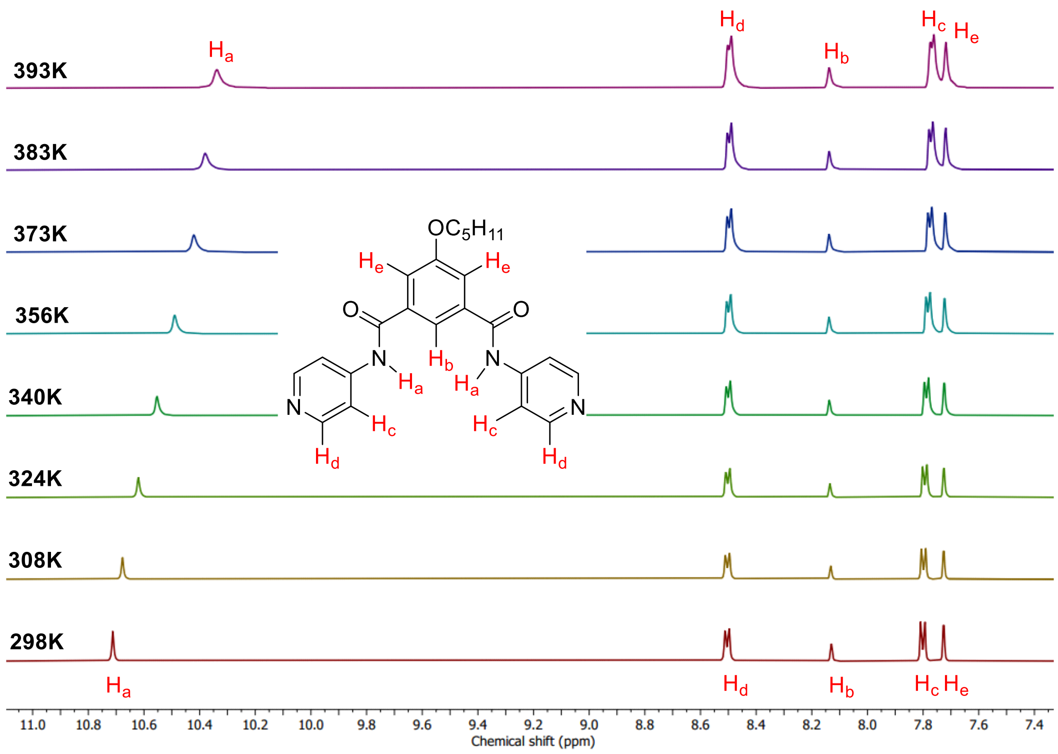

Fig. S3 ^{1}H NMR of channel forming compound **1c** (10 mM) at variable temperature in DMSO-d_6 solvent.

VII. Ion Binding Studies:[S5]

To evaluate the ion binding efficiency of compound **1c** towards anions, ^{1}H NMR titration was carried out with different TBAX salts ($X^- = Cl^-$, Br^-, I^-, NO_3^-, and ClO_4^-). The guest (salts) concentration was increased gradually, and the ^{1}H NMR was recorded after each addition. A significant downfield shift of N-H_a, C-H_b, and C-H_c protons implied that protons H_a, H_b, and H_c are involved in the overall binding of the anions. The BindFit v0.5 software was used to analyze the binding constant with different ions with a 1:1 binding model.

Alongside the protransporter **1c'** was used for the ^{1}H NMR titration with the TBACl salt to understand its ion binding efficiency. Sequential addition of the TBACl salt did not change any peak position of the N-H_a, C-H_b, and C-H_c protons, indicating that, likewise, the compound **1c**, protransporter **1c'** cannot effectively bind with the Cl^- ion in its ion binding pocket.

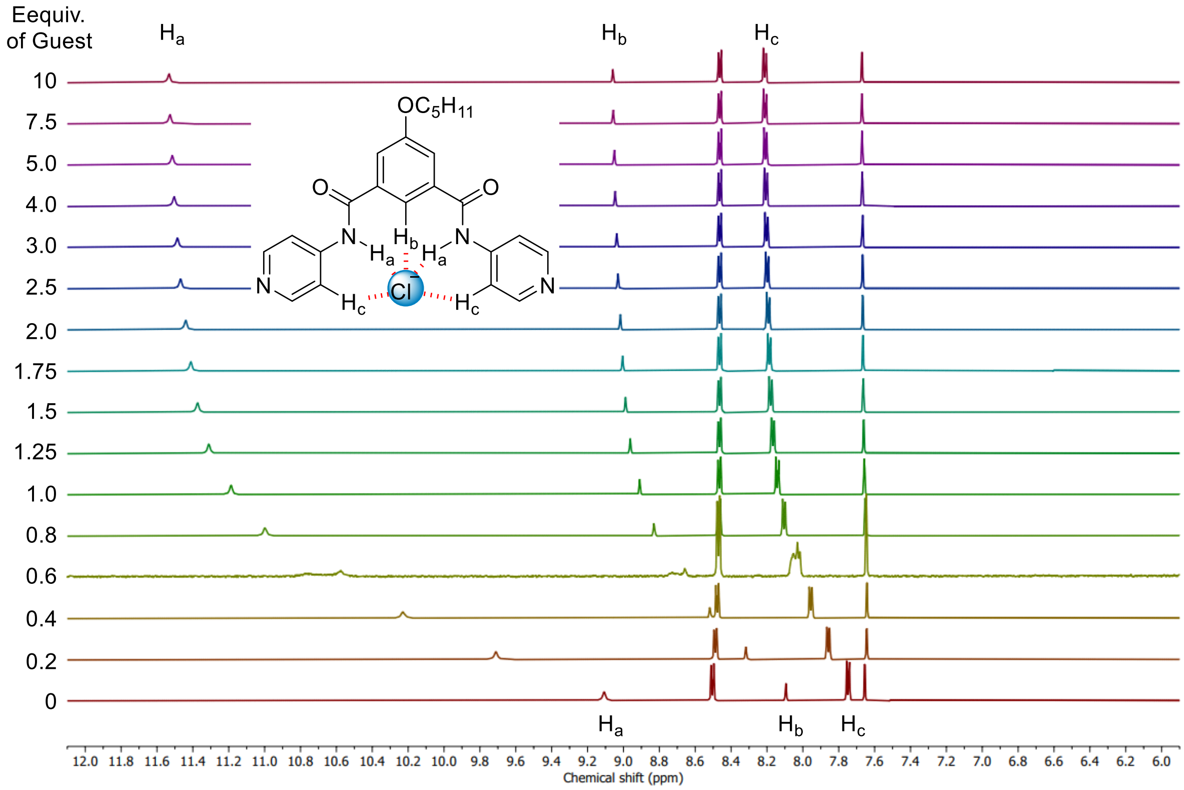

Fig. S4 ^{1}H NMR titration of compound **1c** (2 mM) with TBACl salt in CD_3CN at 25 °C.

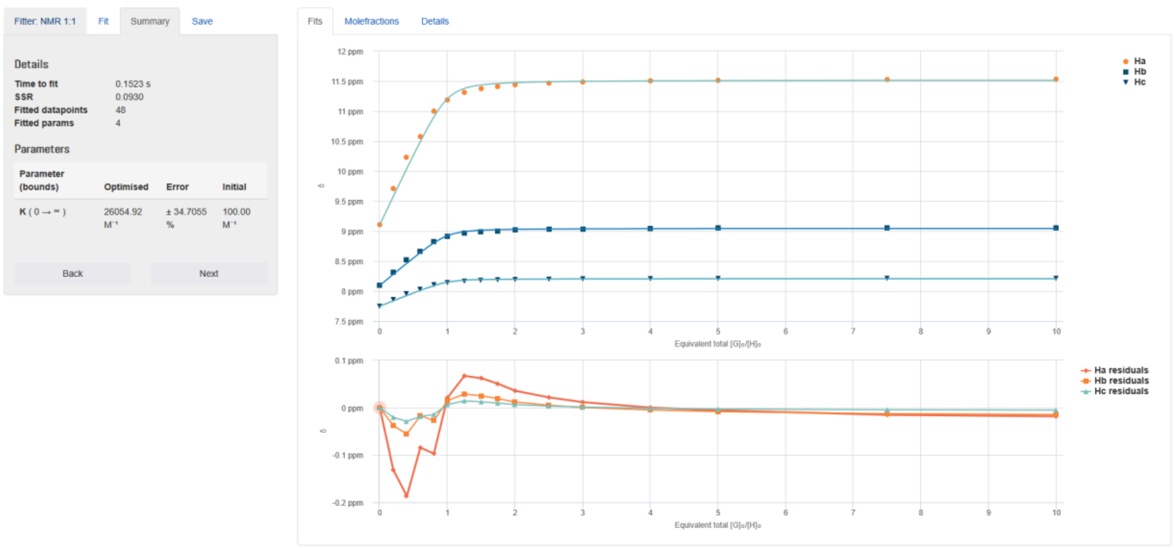

Fig. S5 Screenshot of the fitted data plot from supramolecular.org in BindFit v0.5. The calculated binding constant was found to be 26055 M^{-1} ± 35% in 1:1 receptor-to-anion binding model (left side). The changing pattern of chemical shift and chemical shift residuals with the increasing equivalent of TBACl (right side). The Bindfit URL for this experiment: http://app.supramolecular.org/bindfit/view/e525a88c-a40e-4b65-b79a-ac36bbabf4ea.

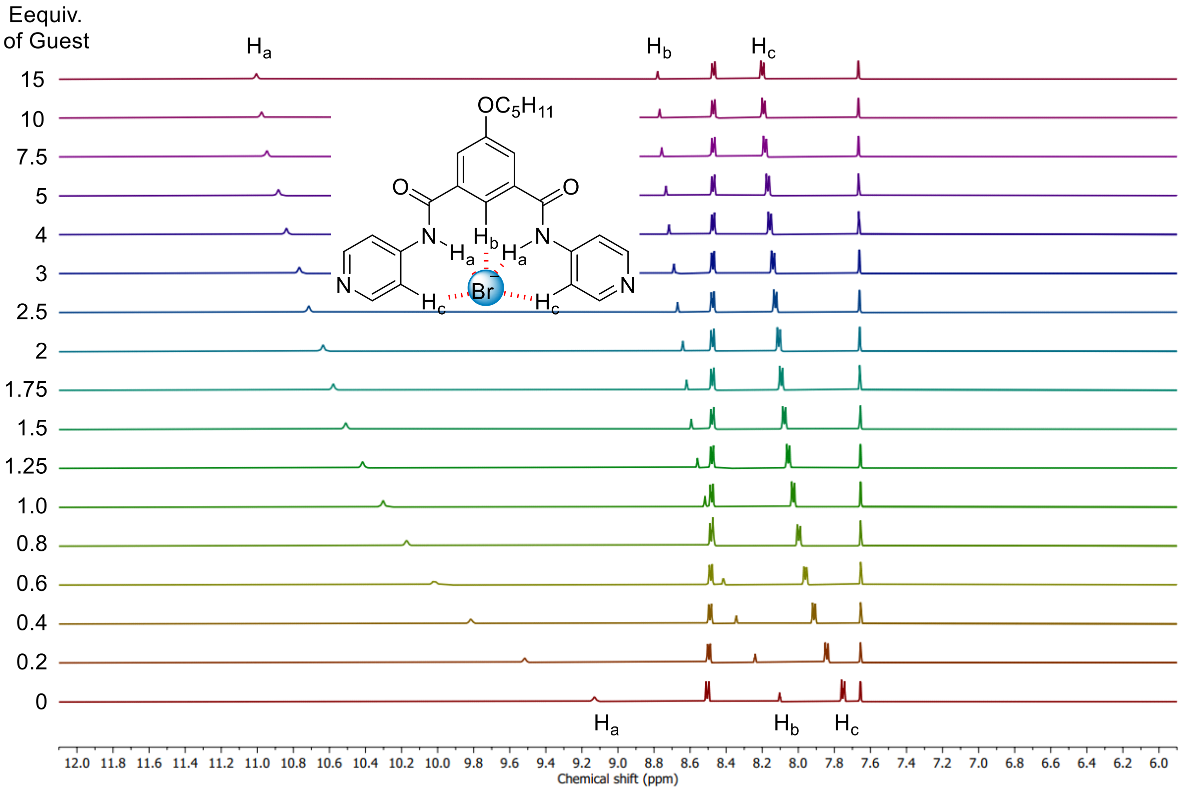

Fig. S6 ^{1}H NMR titration of compound **1c** (2 mM) with TBABr salt in CD_3CN at 25 °C.

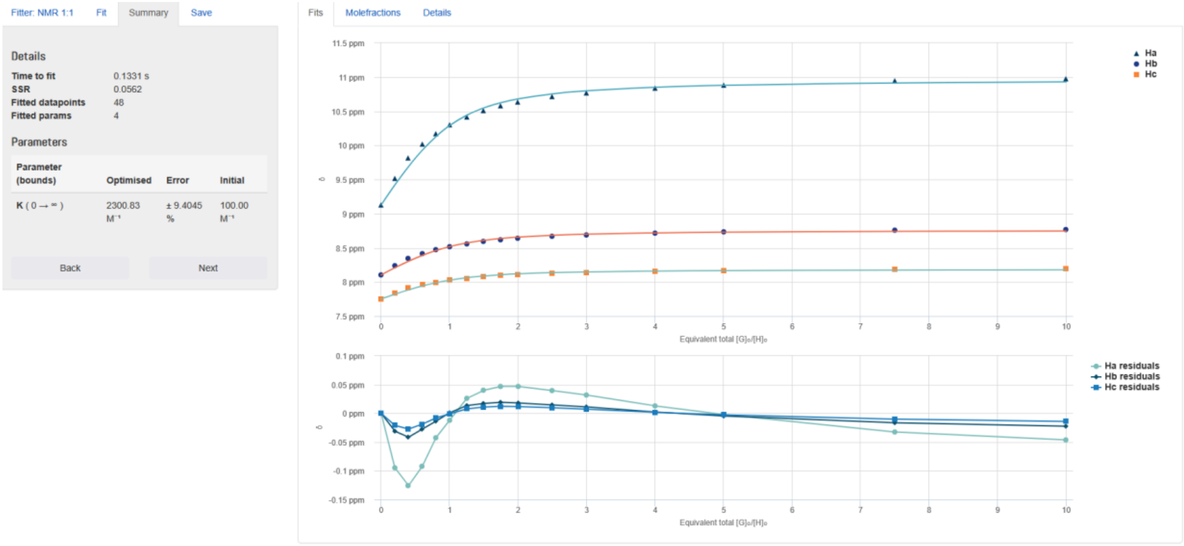

Fig. S7 Screenshot of the fitted data plot from supramolecular.org in BindFit v0.5. The calculated binding constant was found to be 2301 M^{-1} ± 9% in 1:1 receptor-to-anion binding model (left side). The changing pattern of chemical shift and chemical shift residuals with the increasing equivalent of TBABr (right side). The Bindfit URL for this experiment: http://app.supramolecular.org/bindfit/view/8dd988c7-198b-4921-a05b-32194b777f4a.

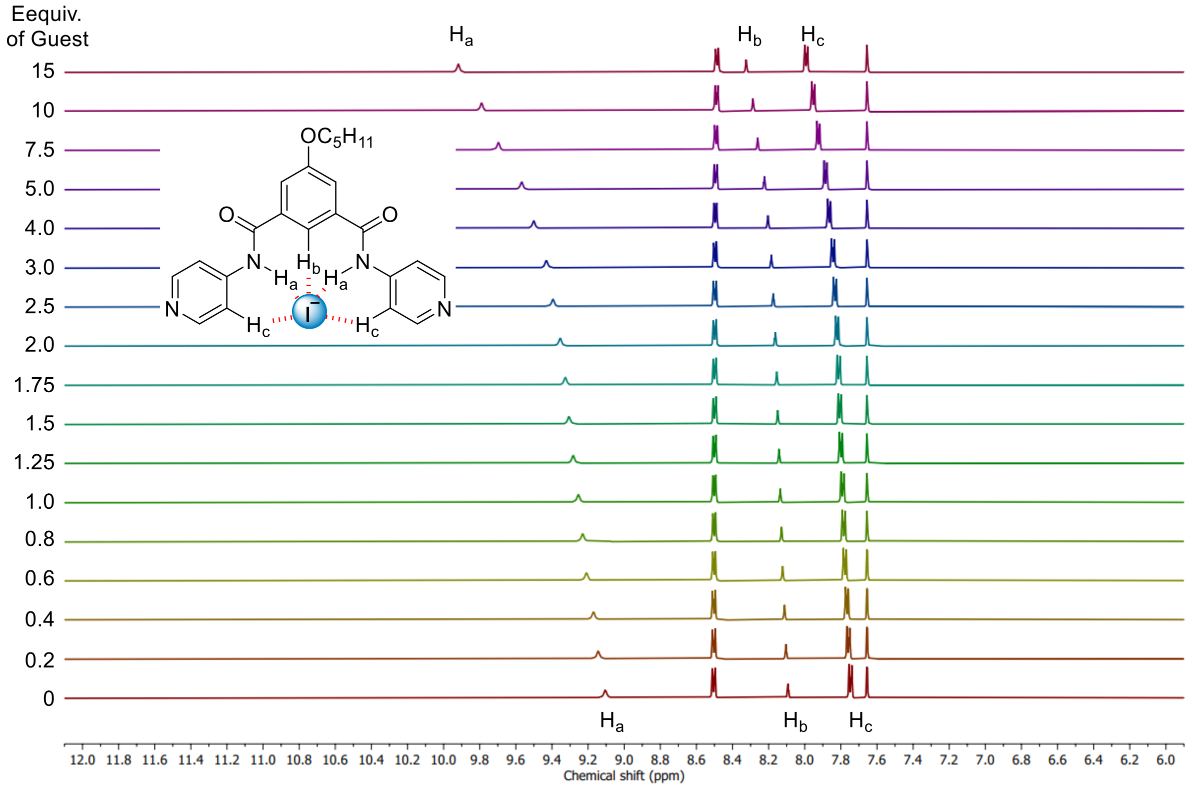

Fig. S8 ^{1}H NMR titration of compound **1c** (2 mM) with TBAI salt in CD_3CN at 25 °C.

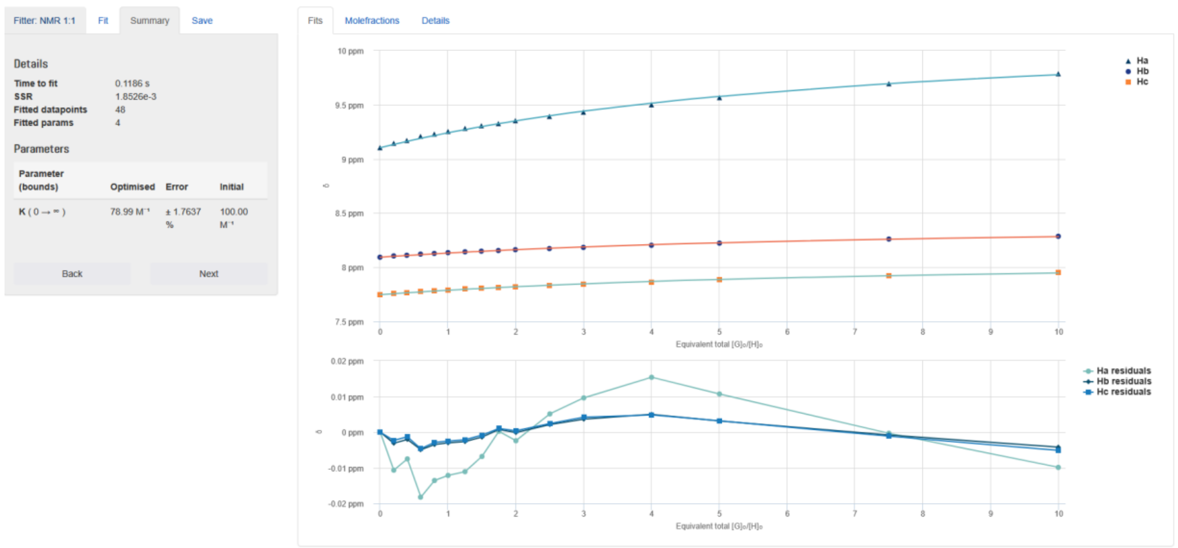

Fig. S9 Screenshot of the fitted data plot from supramolecular.org in BindFit v0.5. The calculated binding constant was found to be 79 M^{-1} ± 2% in 1:1 receptor-to-anion binding model (left side). The changing pattern of chemical shift and chemical shift residuals with the increasing equivalent of TBAI (right side). The Bindfit URL for this experiment: http://app.supramolecular.org/bindfit/view/f39e3f96-8712-4bae-ab14-948aabe74f29.

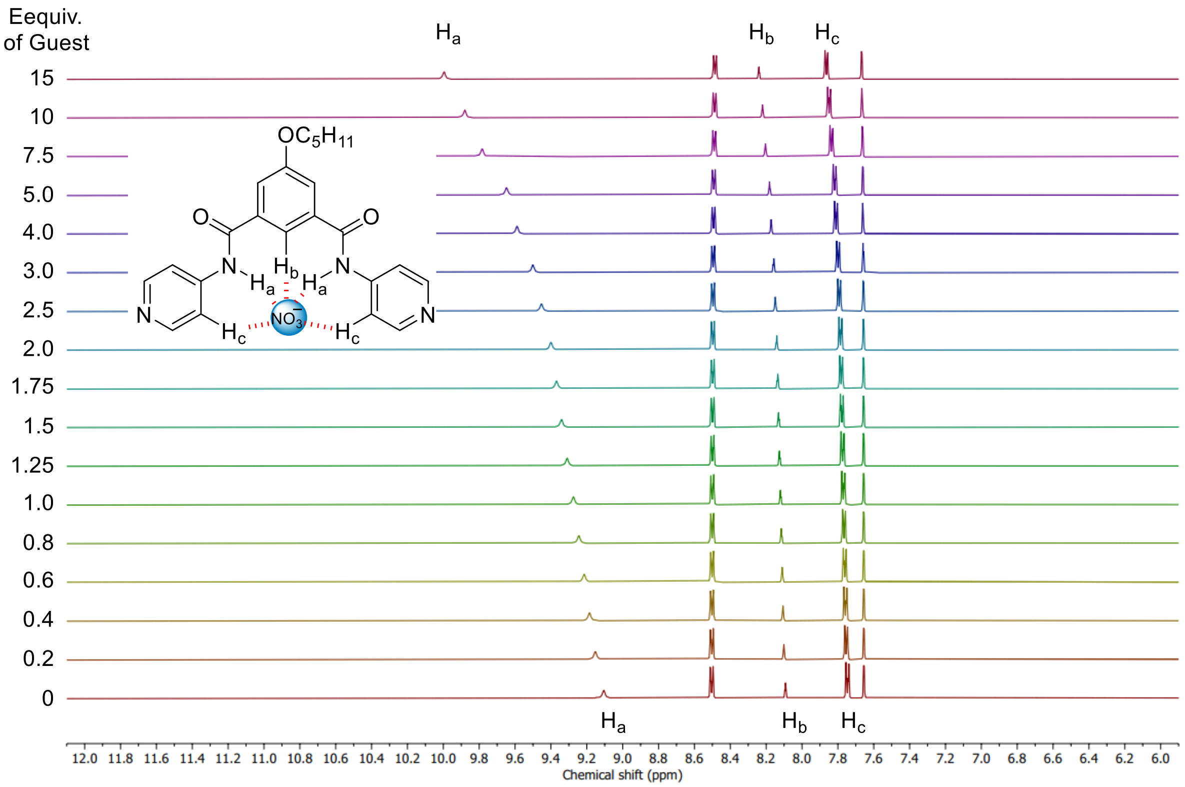

Fig. S10 ^{1}H NMR titration of compound **1c** (2 mM) with $TBANO_3$ salt in CD_3CN at 25 °C.

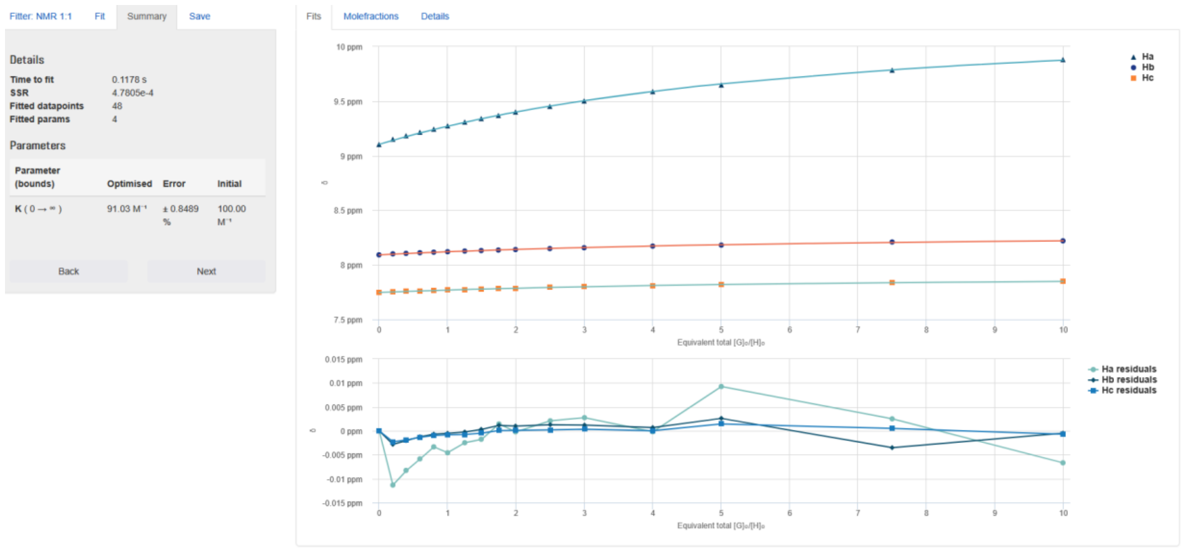

Fig. S11 Screenshot of the fitted data plot from supramolecular.org in BindFit v0.5. The calculated binding constant was found to be 91 M^{-1} ± 1% in 1:1 receptor-to-anion binding model (left side). The changing pattern of chemical shift and chemical shift residuals with the increasing equivalent of $TBANO_3$ (right side). The Bindfit URL for this experiment: http://app.supramolecular.org/bindfit/view/500f37aa-a2a3-48f0-9201-47576a91a122.

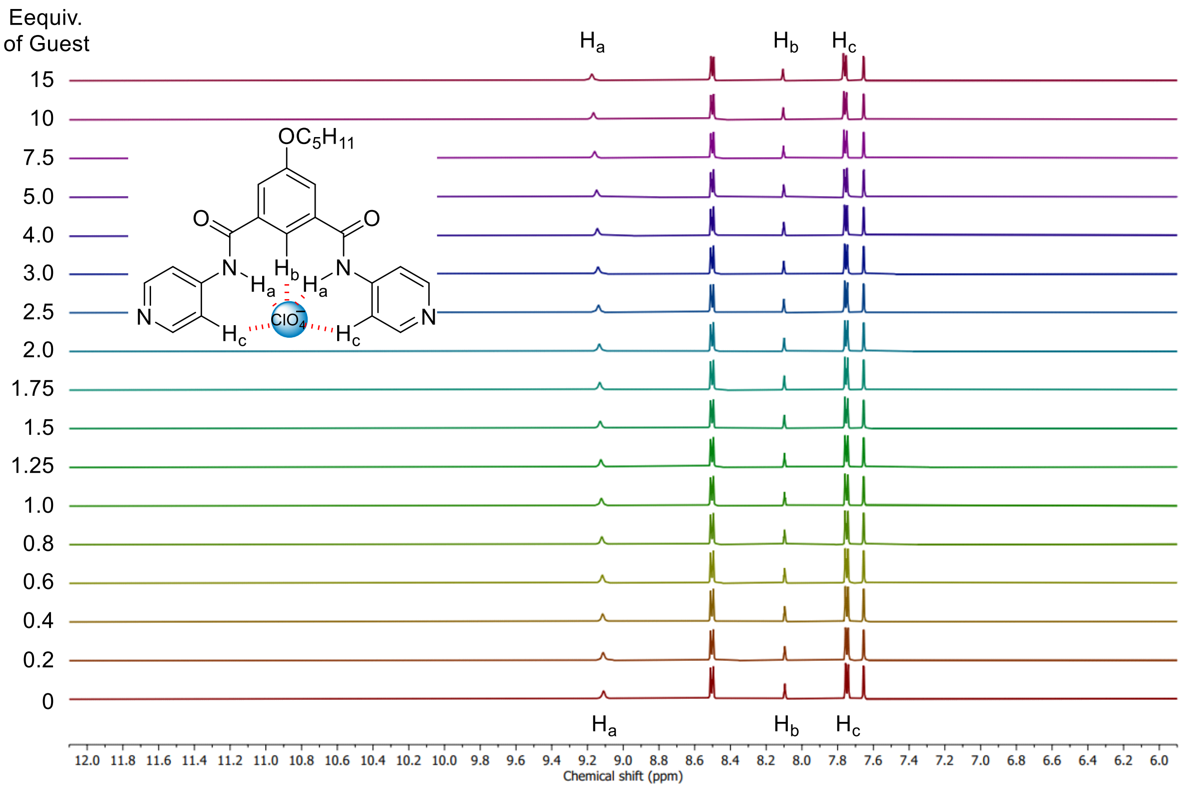

Fig. S12 ^{1}H NMR titration of compound **1c** (2 mM) with $TBAClO_4$ salt in CD_3CN at 25 °C.

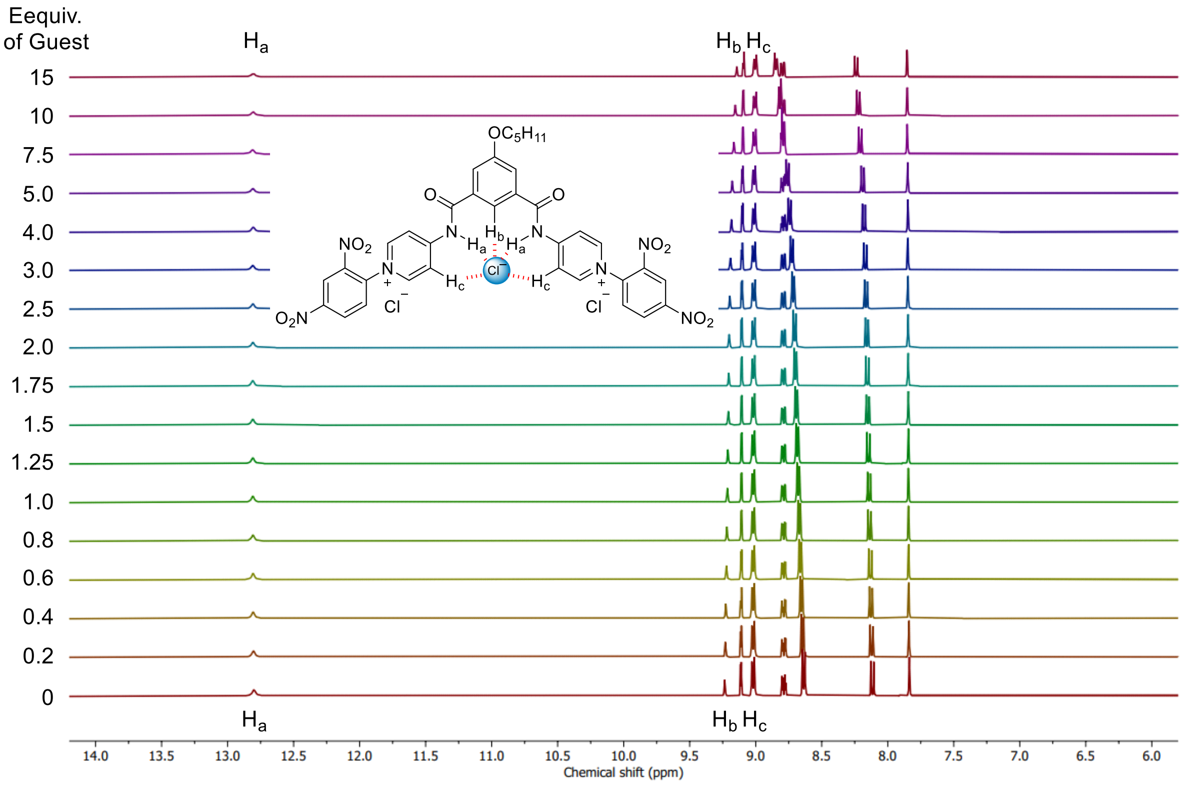

Fig. S13 ^{1}H NMR titration of compound **1c'** (2 mM) with TBACl salt in CD_3CN at 25 °C.

VIII. Mass Spectrometric Studies for Anion Recognition:[S6]

The samples were prepared in CH_3CN by mixing compound **1c** and TBACl. Complexation of **1c** and Cl^- was observed when **1c** and Cl^- were mixed in a 1:1 ratio. The samples were electrosprayed as 20 µM solutions of **1c** and TBACl in CH_3CN at flow rates of 0.4 mL/min. The parameters for the sample cone (40 V) and extractor cone voltage (5 V) were optimized for maximum intensities of the desired complexes, and data were recorded in −ve mode. Signals corresponding to [M+Cl^-] (where, M = exact mass of **1c**) confirmed the formation of a 1:1 complex between the compound **1c** and Cl^- ion.

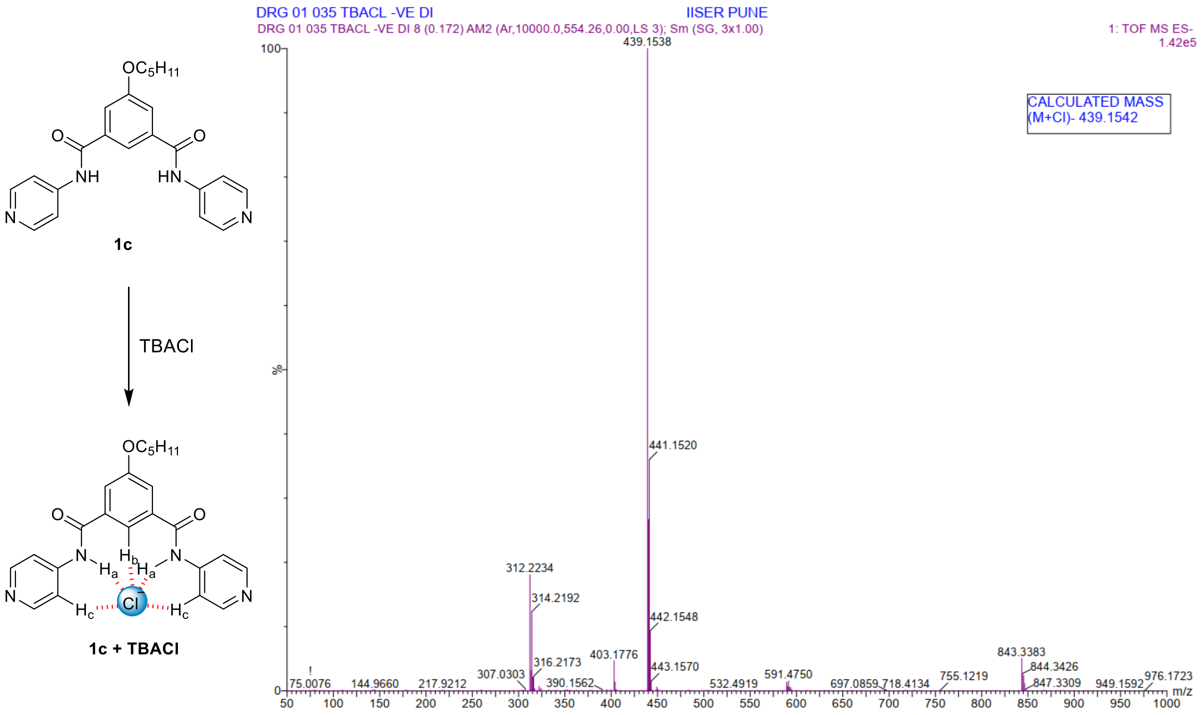

Fig. S14 ESI-MS spectrum recorded from of 1:1 molar mixture of **1c** and TBACl prepared in CH_3CN in −ve mode.

IX. Ion Transport Studies:[S7,S8]

A. Ion transporting activity studies across EYPC–LUVs⊃HPTS:

Preparation of HEPES buffer and stock solutions: The HEPES buffer (pH = 7.0) was prepared by dissolving an appropriate amount of solid HEPES (10 mM) and NaCl (100 mM) in autoclaved water. The pH was adjusted to 7.0 by the addition of aliquots from the NaOH solution (0.5 M). HPLC grade DMSO was used to prepare the stock solution for all the derivatives.

Preparation of EYPC–LUVs⊃HPTS with NaCl: In a dry and clean round-bottom flask (10 mL), 1 mL of egg yolk phosphatidylcholine (EYPC, 25 mg/mL in $CHCl_3$) was dried by purging nitrogen gas with continuous rotation to make a thin transparent film of EYPC. Then, to remove any trace amount of $CHCl_3$, it was kept under high vacuum for 4 h. Further, the dried thin film was hydrated with 1 mL

HEPES buffer (1 mM HPTS, 10 mM HEPES, 100 mM NaCl, pH = 7.0), and the resulting suspension was vortexed for 1 h at 10 min intervals. The hydrated suspension was subjected to 21 freeze-thaw cycles (liquid N_2, 55 °C), followed by extrusion through a 100 nm pore-size polycarbonate membrane 21 times (an odd number of passes), to obtain vesicles with an average diameter of ~100 nm. Finally, size exclusion chromatography using gel filtration (Sephadex G-50) was performed to remove the unentrapped extravesicular HPTS dyes with HEPES buffer (10 mM HEPES, 100 mM NaCl, pH 7.0). Collected vesicles were diluted to 6 mL to get EYPC–LUVs⊃HPTS. Final conditions: ~ 5.0 mM EYPC, Inside: 1 mM HPTS, 10 mM HEPES, 100 mM NaCl, pH = 7.0, Outside: 10 mM HEPES, 100 mM NaCl, pH = 7.0.

Ion transport activity by HPTS assay: In a clean and well-dried fluorescence cuvette, 1975 μL of HEPES buffer (10 mM HEPES, 100 mM NaCl, pH =7.0) and 25 μL of EYPC–LUVs⊃HPTS vesicle were added. The cuvette was placed in a slowly stirring condition using a magnetic stirrer equipped with the fluorescence instrument. The start of the experiment was considered as t = 0 s. The time-dependent HPTS emission intensity was monitored at λ_{em} = 510 nm (λ_{ex} = 450 nm) by creating a pH gradient (~ 0.8) between the intra- and extra-vesicular system by the addition of 20 μL NaOH (0.5 M) at t = 20 s. Then, different concentrations of channel-forming molecules in DMSO were added at t = 100 s. Finally, the vesicles were lyzed by the addition of 10% Triton X-100 solutions (25 μL) at t = 300 s to destroy the pH gradient.

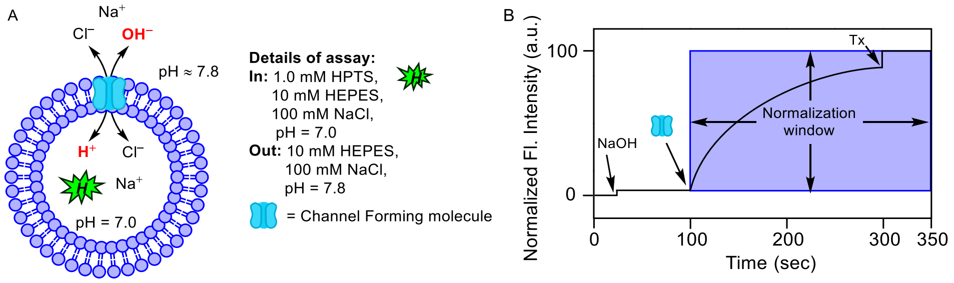

Fig. S15 Schematic representation of ion transport activity across EYPC–LUVs⊃HPTS vesicle (A), and normalization window for the same fluorescence kinetics experiment of ion transport (B).

The time axis was normalized according to Equation S1:

$$t = t - 100 \qquad \text{Equation S1}$$

where, in normalized data, t = 0 s was the timing of compound addition during the experiment, and t = 200 s was the timing of Triton X–100 addition.

The time-dependent data were normalized to fractional fluorescence intensity (in percentage) using Equation S2

$$I_F = [(I_t - I_0) / (I_\infty - I_0)] \times 100 \quad \text{Equation S2}$$

where, I_0 = Fluorescence intensity just before the channel forming molecule addition (at $t = 0$ s), I_∞ = Final fluorescence intensity after addition of Triton X–100, I_t = Fluorescence intensity at time t.

Comparison of ion transport activity in EYPC–LUVs⊃HPTS: To investigate the relative transport activity of channel-forming molecules **1a**–**1e**, and the protransporter **1c'**, an ion transport experiment was done across EYPC–LUVs⊃HPTS. A comparison study with 100 nM concentration revealed the activity sequence of **1c** > **1e** > **1b** > **1d** > **1a** ≈ **1c'**.

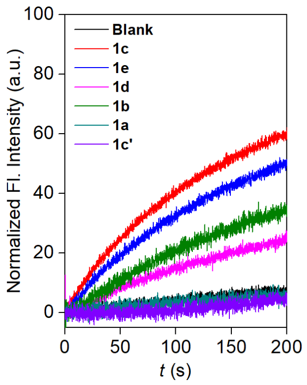

Fig. S16 Activity comparison of the compounds **1a**–**1e** and **1c'** across EYPC–LUVs⊃HPTS at 100 nM concentration.

Dose-response activity in EYPC–LUVs⊃HPTS: The fluorescence kinetics of each channel forming molecule at different concentrations was studied over the course of time. The concentration profile data were evaluated at $t = 290$ s to get the effective concentration, EC_{50} (i.e., the concentration of transporter needed to achieve 50% ion efflux activity) using the Hill equation (Equation S3):

$$Y = Y_\infty + (Y_0 - Y_\infty) / [1 + (c/EC_{50})^n] \quad \text{Equation S3}$$

where, Y_0 = Fluorescence intensity just before the addition of channel forming molecule (at $t = 0$ s), Y_∞ = Fluorescence intensity with excess compound concentration, c = concentration of channel forming molecule, and n = Hill coefficient (i.e., indicative of the number of monomers needed to form an active supramolecule).

Dose-response activity of compounds 1b–1e with NaCl salt:

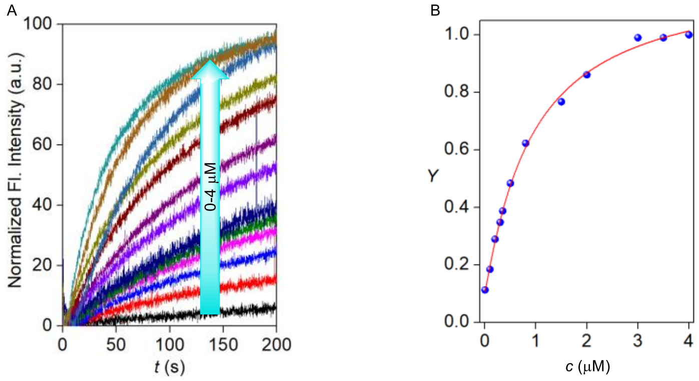

Model	Hill2 (User)		
Equation	y = b+(a-b)/(1+(x/EC50)^n)		
Reduced Chi-Sqr	2.83002E-4		
Adj. R-Square	0.99738		
		Value	Standard Error
D	a	0.10616	0.01625
D	b	1.20148	0.05365
D	n	1.07936	0.08531
D	EC50	0.9315	0.10276

Fig. S17 Concentration-dependent ion transport activity of compound **1b** (0–4 μM) with NaCl salt across EYPC–LUVs⊃HPTS (A), and corresponding Hill plot of compound **1b** at t = 190 s (B).

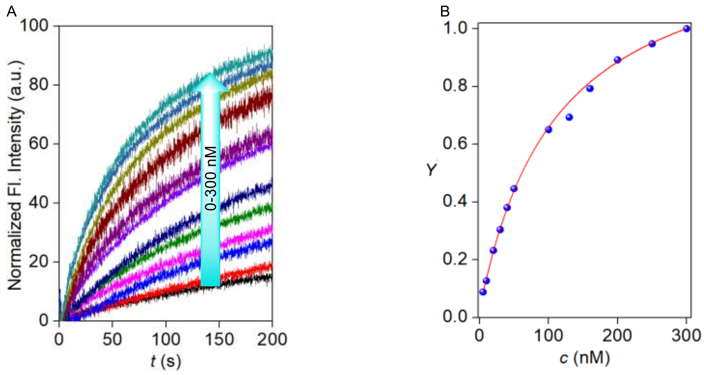

Model	Hill2 (User)		
Equation	y = b+(a-b)/(1+(x/EC50)^n)		
Reduced Chi-Sqr	4.99336E-5		
Adj. R-Square	0.99958		
		Value	Standard Error
D	a	0.02864	0.01344
D	b	1.35073	0.0606
D	n	1.01333	0.06076
D	EC50	109.63794	9.91552

Fig. S18 Concentration-dependent ion transport activity of compound **1c** (0–300 nM) with NaCl salt across EYPC–LUVs⊃HPTS (A), and corresponding Hill plot of compound **1c** at t = 190 s (B).

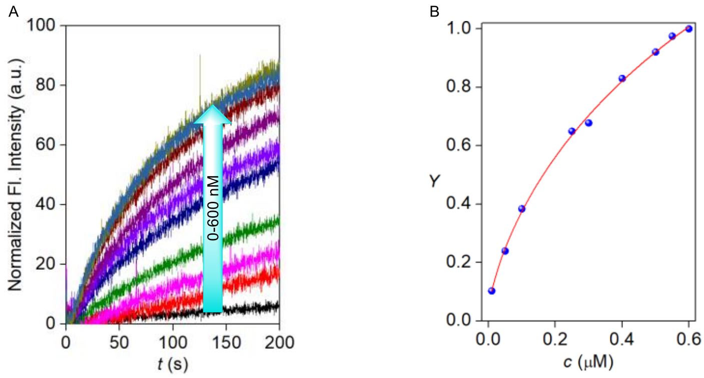

Model	Hill2 (User)		
Equation	y = b+(a-b)/(1+(x/EC50)^n)		
Reduced Chi-Sqr	2.44405E-4		
Adj. R-Square	0.99776		
		Value	Standard Error
C	a	0.02929	0.04481
C	b	3.25473	2.2096
C	n	0.71968	0.16958
C	EC50	1.90498	2.99351

Fig. S19 Concentration-dependent ion transport activity of compound **1d** (0–600 nM) with NaCl salt across EYPC–LUVs⊃HPTS (A), and corresponding Hill plot of compound **1d** at t = 190 s (B).

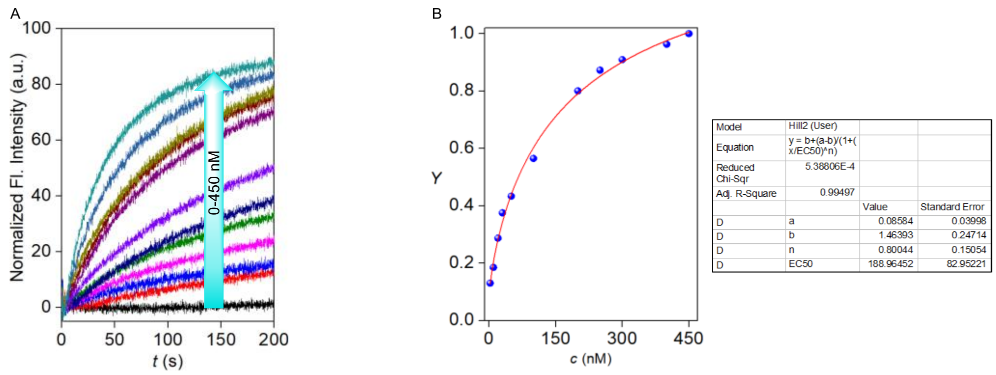

Fig. S20 Concentration-dependent ion transport activity of compound **1e** (0–450 nM) with NaCl salt across EYPC–LUVs⊃HPTS (A), and corresponding Hill plot of compound **1e** at t = 190 s (B).

B. Ion selectivity studies across EYPC–LUVs⊃HPTS:

Buffer and stock solution preparation: HEPES buffer was prepared by dissolving an appropriate amount of solid HEPES and a salt (either NaCl, NaBr, NaI, $NaNO_3$, NaOAc, LiCl, KCl, RbCl, and CsCl) in autoclaved water to get 10 mM HEPES and 100 mM salt, respectively. Subsequently, the pH was adjusted to 7.0 by the addition of 0.5 M NaOH solution. The stock solution of the most active compound **1c** was prepared in HPLC grade DMSO solution for the studies.

Anion selectivity assay: In a clean fluorescence cuvette, 1975 μL of HEPES buffer (10 mM HEPES, 100 mM NaX, at pH = 7.0; where, X^- = Cl^-, Br^-, I^-, NO_3^-, and OAc^-) was added, followed by addition of 25 μL of 100 mM NaCl entrapped EYPC–LUVs⊃HPTS vesicle in slowly stirring condition by a magnetic stirrer equipped with the fluorescence instrument (at t = 0 s). The time-dependent HPTS emission intensity was monitored at λ_{em} = 510 nm (λ_{ex} = 450 nm) by creating a pH gradient (~ 0.8) between the intra- and extra-vesicular system by the addition of 20 μL NaOH (0.5 M) at t = 20 s. The channel forming molecule **1c** was added at t = 100 s, and at t = 300 s, 25 μL of 10% Triton X–100 was added to lyze all vesicles for the complete destruction of the pH gradient. For data analysis and comparison, time (X-axis) was normalized between the point of addition of channel forming molecule (i.e., t = 100 s was normalized to t = 0 s) and the end point of the experiment (i.e., t = 300 s was normalized to t = 200 s) using Equation S1. Fluorescence intensities (I_t) were normalized to fractional emission intensity I_F using Equation S2.

Cation selectivity assay: In a clean fluorescence cuvette, 1975 μL of HEPES buffer (10 mM HEPES, 100 mM MCl, at pH = 7.0; where, M^+ = Li^+, Na^+, K^+, Rb^+, and Cs^+) was added, followed by addition of 25 μL of 100 mM NaCl entrapped EYPC–LUVs⊃HPTS vesicle in slowly stirring condition by a magnetic stirrer equipped with the fluorescence instrument (at t = 0 s). The time-dependent HPTS emission intensity was monitored at λ_{em} = 510 nm (λ_{ex} = 450 nm) by creating a pH gradient (~ 0.8) between the intra- and extra-vesicular system by the addition of 20 μL NaOH (0.5 M) at t = 20 s. The channel forming molecule **1c** was added at t = 100 s, and at t = 300 s, 25 μL of 10% Triton X–100 was

added to lyze all vesicles for the complete destruction of the pH gradient. For data analysis and comparison, time (X-axis) was normalized between the point of addition of channel forming molecule (i.e., t = 100 s was normalized to t = 0 s) and the endpoint of the experiment (i.e., t = 300 s was normalized to t = 200 s) using Equation S1. Fluorescence intensities (I_t) were normalized to fractional emission intensity I_F using Equation S2.

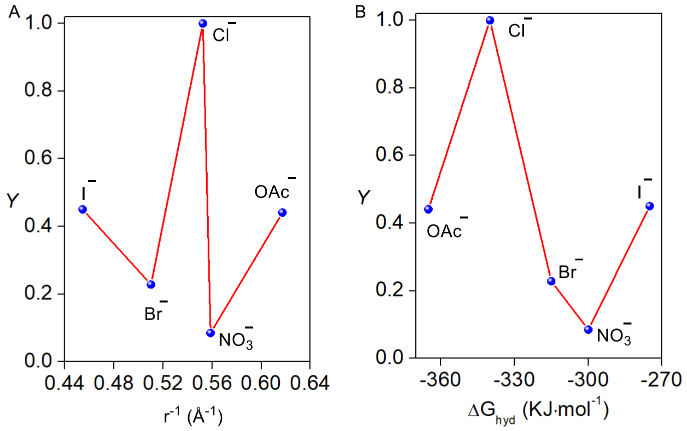

Fig. S21 Fractional activity Y (relative to Cl^-) as a function of the reciprocal anion radius (A), and fractional activity Y (relative to Cl^-) as a function of the anion hydration energy (B).

C. Chloride transport activity across EYPC–LUVs⊃lucigenin vesicles:

Buffer and stock solution preparation: HEPES buffer was prepared by dissolving an appropriate amount of solid HEPES and $NaNO_3$ salt in autoclaved water to achieve 10 mM HEPES and 200 mM $NaNO_3$, respectively. Subsequently, the pH was adjusted to 7.0 by adding 0.5 M NaOH solution. The stock solution of the most active compound **1c** was prepared in HPLC grade CH_3CN solution for the studies.

Preparation of EYPC–LUVs⊃lucigenin: In a clean and dry small (10 mL) round-bottom flask, 1 mL egg yolk phosphatidylcholine (EYPC, 25 mg/mL stock in $CHCl_3$) was added. The solution was dried by purging nitrogen with continuous rotation to form a thin transparent film of EYPC. The transparent film was kept in a high-vacuum environment for 4 h to remove all traces of $CHCl_3$ at room temperature. The resulting film was hydrated with 1 mL buffer solution (1 mM lucigenin, 10 mM HEPES, and 200 mM $NaNO_3$, pH = 7.0), and the resulting suspension was vortexed at 10 min intervals for 1 h. The hydrated suspension was subjected to 21 freeze-thaw cycles (liquid N_2, 55 °C), followed by extrusion through a 200 nm pore-size polycarbonate membrane 21 times (an odd number of passes), to obtain vesicles with an average diameter of ~200 nm. Extravesicular dyes were removed by gel filtration (using Sephadex G-50) with buffer solution (10 mM HEPES and 200 mM $NaNO_3$, pH = 7.0), and diluted to 4 mL to get EYPC–LUVs⊃lucigenin. Final conditions: ~ 5 mM EYPC; Inside: 1 mM lucigenin, 10 mM HEPES, 200 mM $NaNO_3$, pH = 7.0; Outside: 10 mM HEPES, 200 mM $NaNO_3$, pH = 7.0.

Dose-dependent Cl^- transport by lucigenin assay: In a clean and dry fluorescence cuvette, 1975 µL of buffer solution (10 mM HEPES, 200 mM $NaNO_3$, and pH = 7.0) and 25 µL EYPC–LUVs⊐lucigenin was taken. This suspension was placed in a slow stirring condition in the fluorescence instrument equipped with a magnetic stirrer. The start of the experiment was considered as $t = 0$ s. The fluorescence intensity of lucigenin was monitored at λ_{em} = 535 nm (λ_{ex} = 455 nm) over the course of time. The chloride gradient was created by the addition of 2.0 M NaCl (33.3 µL) at $t = 20$ s between the intra- and extra-vesicular systems, followed by the addition of channel-forming molecule **1c** at $t = 100$ s. Finally, vesicles were lyzed by adding 10% Triton X–100 (25 µL) at $t = 300$ s for the destruction of the chloride gradient.

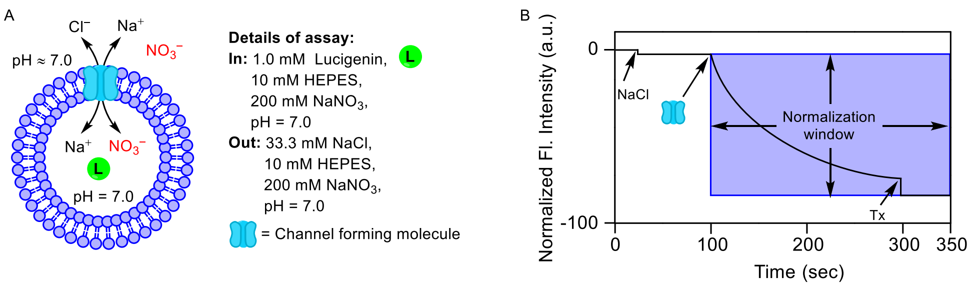

Fig. S22 Schematic representation of ion transport activity across EYPC–LUVs⊐lucigenin vesicle (A), and normalization window for the same fluorescence kinetics experiment of ion transport (B).

The time-dependent data were normalized to fractional (in percentage) fluorescence intensity using Equation S4:

$$I_F = [(I_t - I_0) / (I_\infty - I_0)] \times (-100) \qquad \text{Equation S4}$$

where, I_0 = Fluorescence intensity just before the channel forming molecule addition (at $t = 0$ s). I_∞ = Final fluorescence intensity after the addition of Triton X−100. I_t = Fluorescence intensity at time t.

For data analysis and comparison, time (X−axis) was normalized between the point of compound addition (i.e., $t = 100$ s was normalized to $t = 0$ s) and the endpoint of the experiment (i.e., $t = 300$ s was normalized to $t = 200$ s).

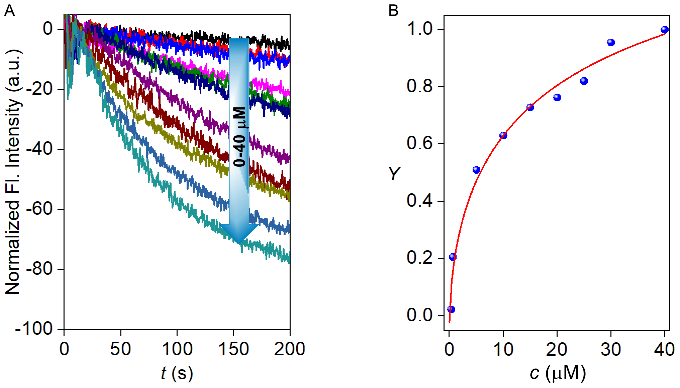

Model	Hill2 (User)		
Equation	y = b+(a-b)/(1+(x /EC50)^n)		
Reduced Chi-Sqr	0.00265		
Adj. R-Square	0.98048		
		Value	Standard Error
D	a	-0.01992	0.05047
D	b	1.73008	0.74343
D	n	0.59387	0.17827
D	EC50	24.22644	34.09389

Fig. S23 Chloride influx study of compound **1c** (0–40 μM) across EYPC–LUVs⊃lucigenin (A), and corresponding Hill plot of compound **1c** at t = 190 s (B).

Cation selectivity assay across EYPC–LUVs⊃lucigenin vesicles: The vesicles were prepared using the same procedure as discussed above.

Details of the assay: In a clean and dry fluorescence cuvette, 1975 μL of buffer solution (10 mM HEPES, 200 mM $NaNO_3$, and pH = 7.0) and 25 μL EYPC–LUVs⊃lucigenin were taken. The suspension was kept in a slow stirring condition in the fluorescence instrument equipped with a magnetic stirrer. The start of the experiment was considered as t = 0 s. The quenching of lucigenin fluorescence intensity was monitored over time at λ_{em} = 535 nm (λ_{ex} = 455 nm). At t = 20 s, the chloride gradient was created by the addition of 2 M chloride salts (33.3 μL) of different cations MCl ($M^+ = Li^+$, Na^+, K^+, Rb^+, and Cs^+), followed by the addition of channel forming molecule **1c** at t = 100 s. Finally, vesicles were lyzed by the addition of 10% Triton X−100 (25 μL) at t = 300 s to diminish the applied chloride gradient. The time-dependent data were normalized to fractional (in percentage) fluorescence intensity using Equation S4.

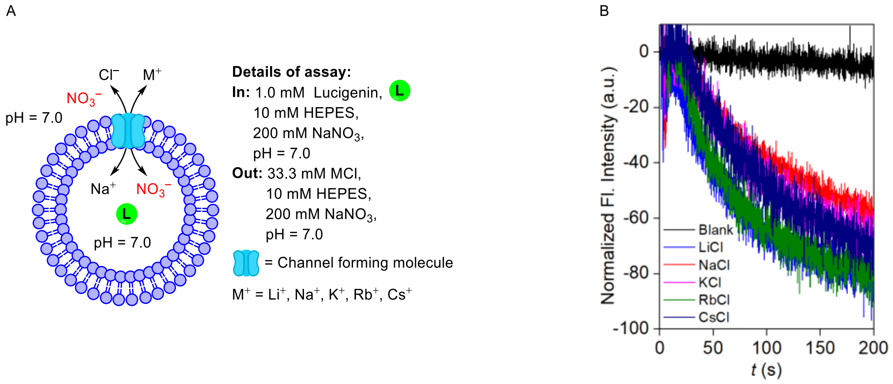

Fig. S24 Schematic representation of cation selectivity assay across EYPC–LUVs⊃lucigenin (A), Cation selectivity of channel forming compound **1c** (10 μM) by varying extravesicular cations (B).

D. Mechanistic study of ion transport across EYPC−LUVs⊐Lucigenin:

Cl^- transport by lucigenin assay in the presence of valinomycin:[S9] In a clean and dry fluorescence cuvette, 1975 µL of buffer solution (10 mM HEPES, 200 mM $NaNO_3$, and pH = 7.0) and 25 µL EYPC–LUVs⊐lucigenin were taken and slowly stirred in a fluorescence instrument equipped with a magnetic stirrer. The start of the experiment was considered as $t = 0$ s. The time-dependent fluorescence intensity of lucigenin was monitored at $\lambda_{em} = 535$ nm ($\lambda_{ex} = 455$ nm). A solution of 2 M KCl (33.3 µL) was added at $t = 20$ s to create a chloride gradient between the intra- and extra-vesicular system, followed by the addition of valinomycin (1 µM) at $t = 50$ s and channel-forming molecule **1c** (200 nM) at $t = 100$ s. Finally, the destruction of the chloride gradient was done by the addition of 10 % Triton X−100 (25 µL) at $t = 300$ s. The time-dependent data were normalized to fractional (in percentage) fluorescence intensity using Equation S4.

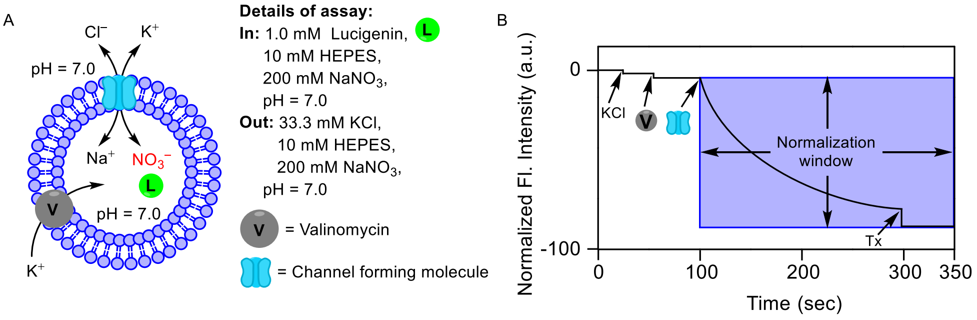

Fig. S25 Schematic representation of valinomycin assay across EYPC–LUVs⊐lucigenin (A), and normalization window for the same fluorescence kinetics experiment of ion transport (B).

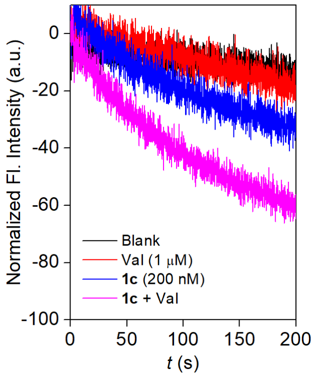

Fig. S26 Chloride influx by channel **1c** in the presence and absence of valinomycin across EYPC–LUVs⊐lucigenin.

NO_3^-/SO_4^{2-} assay:[S10]

Buffer and stock solution preparation: HEPES buffer was prepared by dissolving an appropriate amount of solid HEPES and NaCl salt in autoclaved water to get 10 mM HEPES and 200 mM NaCl salt, respectively. Subsequently, the pH was adjusted to 7.0 by adding 0.5 M NaOH solution. Similarly,

iso-osmolar $NaNO_3$ buffer (10 mM HEPES and 200 mM $NaNO_3$, pH = 7.0) and Na_2SO_4 (10 mM HEPES and 66.6 mM Na_2SO_4, pH = 7.0) buffer solutions were prepared. The stock solution of the most active compound **1c** was prepared in HPLC grade CH_3CN solution for the studies.

Preparation of EYPC–LUVs⊃lucigenin: Lucigenin vesicles were prepared by following the same protocol as mentioned above. Final conditions: ~ 5 mM EYPC; Inside: 1 mM lucigenin, 10 mM HEPES, 200 mM NaCl, pH = 7.0; Outside: 10 mM HEPES, 200 mM NaCl, pH = 7.0.

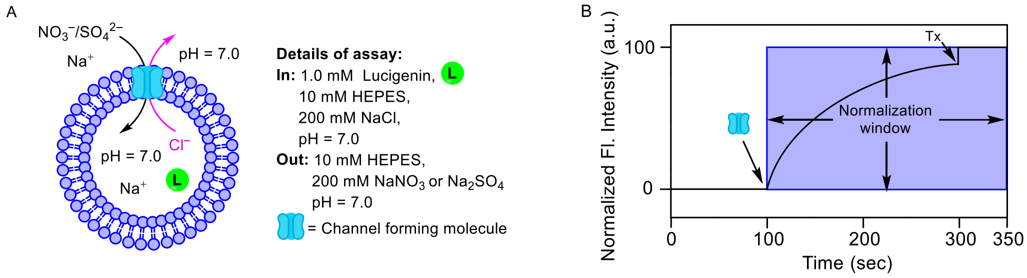

Fig. S27 Schematic representation of NO_3^-/SO_4^{2-} assay across EYPC–LUVs⊃lucigenin (A), and normalization window for same fluorescence kinetics experiment of ion transport (B).

Details of the assay: In clean and dry fluorescence cuvette, 1950 µL of buffer solution (either 10 mM HEPES, 200 mM $NaNO_3$ and pH = 7.0 or 10 mM HEPES, 66.6 mM Na_2SO_4 and pH = 7.0) and 50 µL EYPC–LUVs⊃lucigenin were taken and slowly stirred in fluorescence instrument equipped with a magnetic stirrer. The start of the experiment was considered as t = 0 s. Channel-forming molecule **1c** (25 µM) was added at t = 100 s. The time-dependent fluorescence intensity of lucigenin was monitored at λ_{em} = 535 nm (λ_{ex} = 455 nm). Finally, the chloride gradient was destroyed by adding 10 % Triton X−100 (25 µL) at t = 300 s. The time-dependent data were normalized to fractional (in percentage) fluorescence intensity using Equation S2.

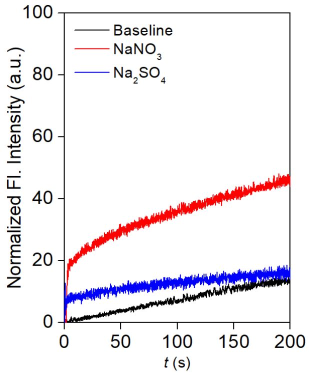

Fig. S28 Chloride efflux by channel **1c** across EYPC–LUVs⊃lucigenin in the presence of iso-osmolar $NaNO_3$ and Na_2SO_4 as extravesicular buffer.

E. Evaluation of membrane stability and channel nature:[S10]

Preparation of EYPC-LUVs⊃CF vesicles: In a clean and dry small (10 mL) round bottom flask, 0.5 mL egg yolk phosphatidylcholine (EYPC, 25 mg/mL stock in $CHCl_3$) was added.

A thin lipid film was prepared by evaporating a chloroform solution of EYPC under a stream of N_2, followed by drying under high vacuum for 4 h to remove residual $CHCl_3$. The lipid film was then hydrated with 0.5 mL of buffer (10 mM HEPES, 10 mM NaCl, 50 mM CF, pH 7.0) for 1 h with occasional vortexing (4–5 times) and subsequently subjected to 23 freeze-thaw cycles. The resulting vesicle suspension was extruded through a 200 nm pore size polycarbonate membrane. Unencapsulated dye was removed by size-exclusion chromatography (Sephadex G-50) using HEPES buffer (10 mM HEPES, 100 mM NaCl, pH 7.0). Final concentration: ~ 2.5 mM EYPC lipid; intravesicular solution: 10 mM HEPES, 10 mM NaCl, 50 mM CF, pH 7.0; extravesicular solution: 10 mM HEPES, 100 mM NaCl, pH 7.0.

CF assay details: In a clean and dry fluorescence cuvette, 1950 μL of HEPES buffer solution (10 mM HEPES, 100 mM NaCl, pH 7.0) and 50 μL EYPC–LUVs⊃CF were taken. The suspension was kept in a slow stirring condition in the fluorescence instrument equipped with a magnetic stirrer at t = 0 s. The fluorescence intensity was monitored over time at λ_{em} = 517 nm (λ_{ex} = 492 nm). At t = 100 s, channel forming compound **1c** was added to it at different concentrations. Finally, vesicles were lyzed by the addition of 10% Triton X–100 (25 μL) at t = 300 s for 100% efflux of CF dyes. The time-dependent data were normalized to fractional (in percentage) fluorescence intensity using Equation S2, and the time axis (X–axis) was normalized using Equation S1.

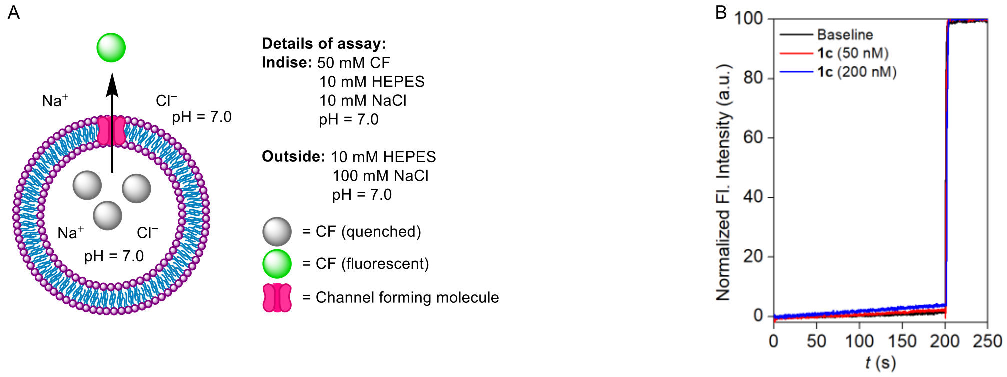

Fig. S29 Schematic representation of the CF assay across EYPC–LUVs⊃CF (A) and fluorescence kinetics experiment of channel forming compound **1c** at different concentrations (B).

F. Evaluation of the mode of ion transport by DPPC assay:[S11,S12]

Buffer and stock solution preparation: HEPES buffer was prepared by dissolving an appropriate amount of solid HEPES and NaCl salt in autoclaved water to get 10 mM HEPES and 100 mM NaCl, respectively. Subsequently, the pH was adjusted to 7.0 by the addition of 0.5 M NaOH solution. The

stock solution of the most active compound **1c** was prepared using an HPLC grade DMSO solution for the studies.

Preparation of vesicles: Both EYPC–LUVs⊃HPTS were prepared using the above-mentioned method. During the preparation of the vesicles, the required temperature (either 25 °C or 45 °C) was maintained during the extrusion process.

Details of the assay: In a clean and well-dry fluorescence cuvette, 1975 µL of HEPES buffer (10 mM HEPES, 100 mM NaCl, pH =7.0) and 25 µL of EYPC–LUVs⊃HPTS vesicle were added into it. The cuvette was placed in a slowly stirring condition using a magnetic stirrer equipped with the fluorescence instrument (t = 0 s). The time-dependent HPTS emission intensity was monitored at λ_{em} = 510 nm (λ_{ex} = 450 nm) by creating a pH gradient (~ 0.8) between the intra- and extra-vesicular system by the addition of 20 µL NaOH (0.5 M) at t = 20 s. Compound **1c** (100 nM) was added at t = 100 s. Finally, the vesicles were lyzed by the addition of 10% Triton X−100 solutions (25 µL) at t = 300 s to destroy the pH gradient.

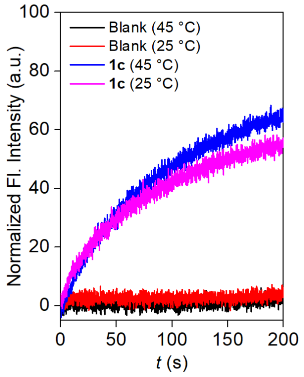

Fig. S30 Fluorescence kinetics experiment of DPPC assay with channel forming compound **1c** (25 µM) at 25 °C and 45 °C.

X. Planar Bilayer Conductance Measurements:[S13]

Diphytanoylphosphatidylcholine (Avanti Polar Lipids), dissolved in n-decane (20 mg/mL), was used to form a Bilayer lipid membrane (BLM) across an aperture of 150 µM diameter in a polystyrene cup (Warner Instrument, USA). Both cis and trans chambers were filled with symmetrical unbuffered 1M KCl solution. The cis chamber was connected to the BC 535 head stage (Warner Instrument, USA) by an Ag-AgCl electrode, and the trans chamber was held at virtual ground. Compound **1c** was added to the cis chamber (working concentration 5 µM), and the solution was stirred with a magnetic stirrer for 15 min. The addition of compound **1c** rapidly triggered the current flow with open-close transition events at different holding potentials, confirming the formation of ion channels inside the bilayer membrane. Currents were low pass filtered at 1 kHz using pClamp9 software (Molecular Probes, USA)

and an analog-to-digital converter (Digidata 1440, Molecular Probes). All data were analyzed using the software pClamp 10.7.

The average current was calculated from the trace, and then conductance and other calculations were made accordingly.

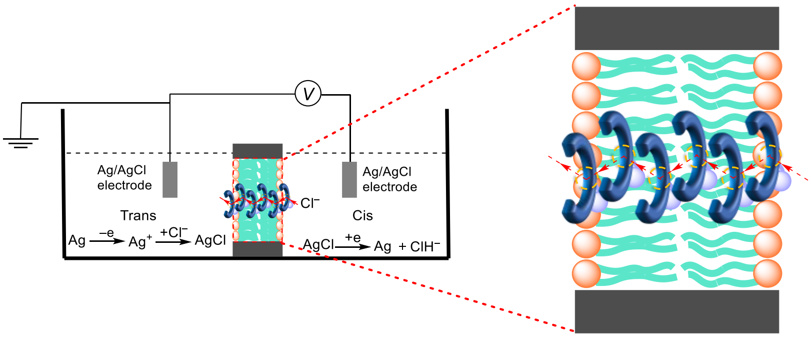

Fig. S31 Systematic representation of bilayer lipid membrane experiment.

A. Channel diameter calculation:

Channel diameter was calculated by using Hille's equation,

$$1/g = (l + \pi d/4) \times (4\rho/\pi d^2) \qquad \text{Equation S5}$$

where, g = corrected conductance (obtained by multiplying measured conductance with the Sansom's correction factor 5.61), l = thickness of the membrane = 34 Å, ρ = resistivity of 1 M KCl solution, and d = diameter of the ion channel.

The calculated average diameter from Hille's equation for channel **1c** is 4.7 ± 0.3 Å.

B. Single-channel conductance calculation:

Table. S1: Table for experimentally calculated single-channel conductance of compound **1c**.

Entry	Slope	G (pS)
**1**	0.16622	166.22
**2**	0.16596	165.96
**3**	0.16431	164.31
**4**	0.16526	165.26
**5**	0.16641	166.41
		Average = 165.63

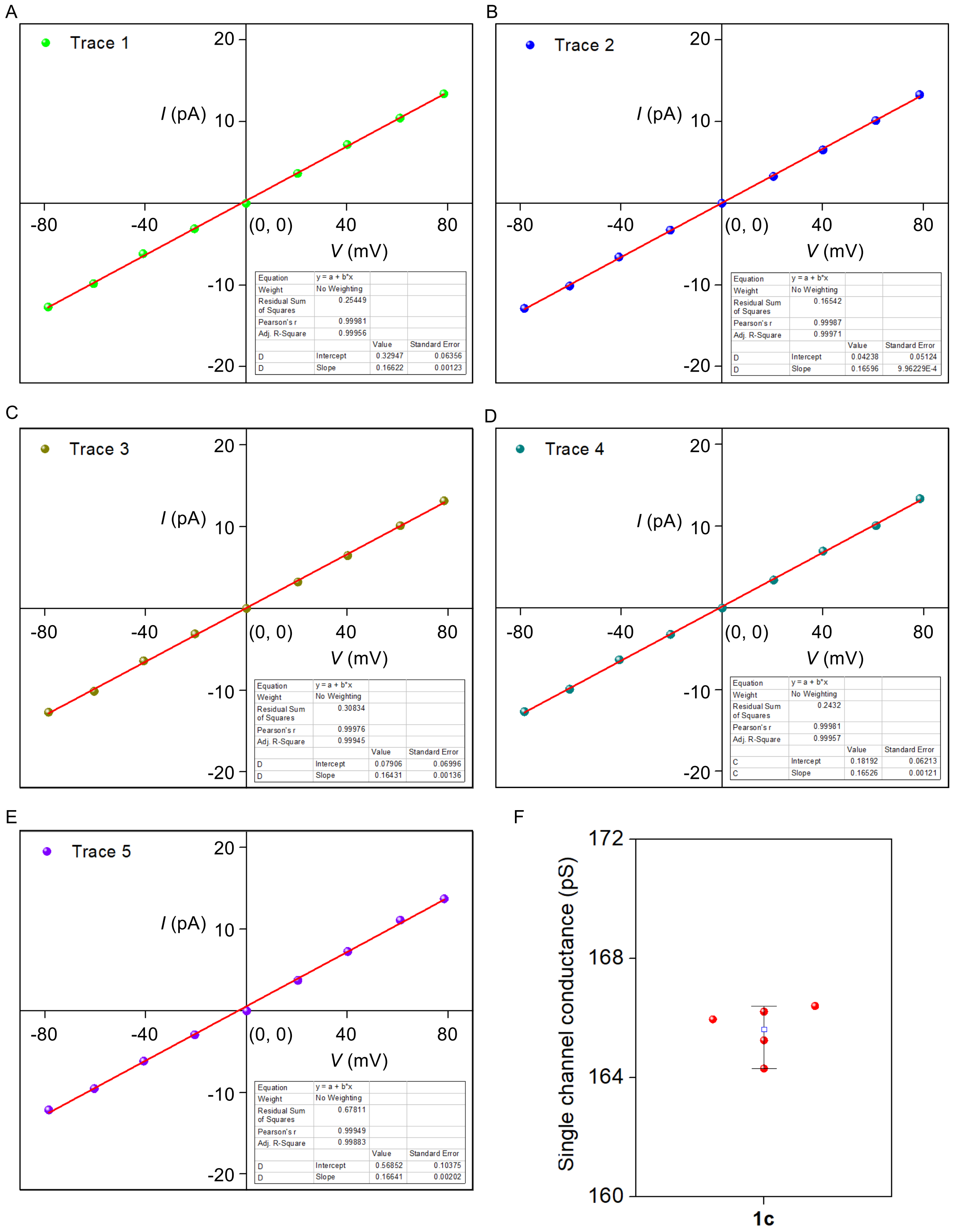

Fig. 32 All five traces of *I*–*V* plots of **1c** in symmetrical (cis/trans = 1 M/1 M) KCl solutions (A, B, C, D, E); box plot of the average single channel conductance obtained from five experiments (represented as red circles) (F).

C. Anion/cation selectivity in BLM:

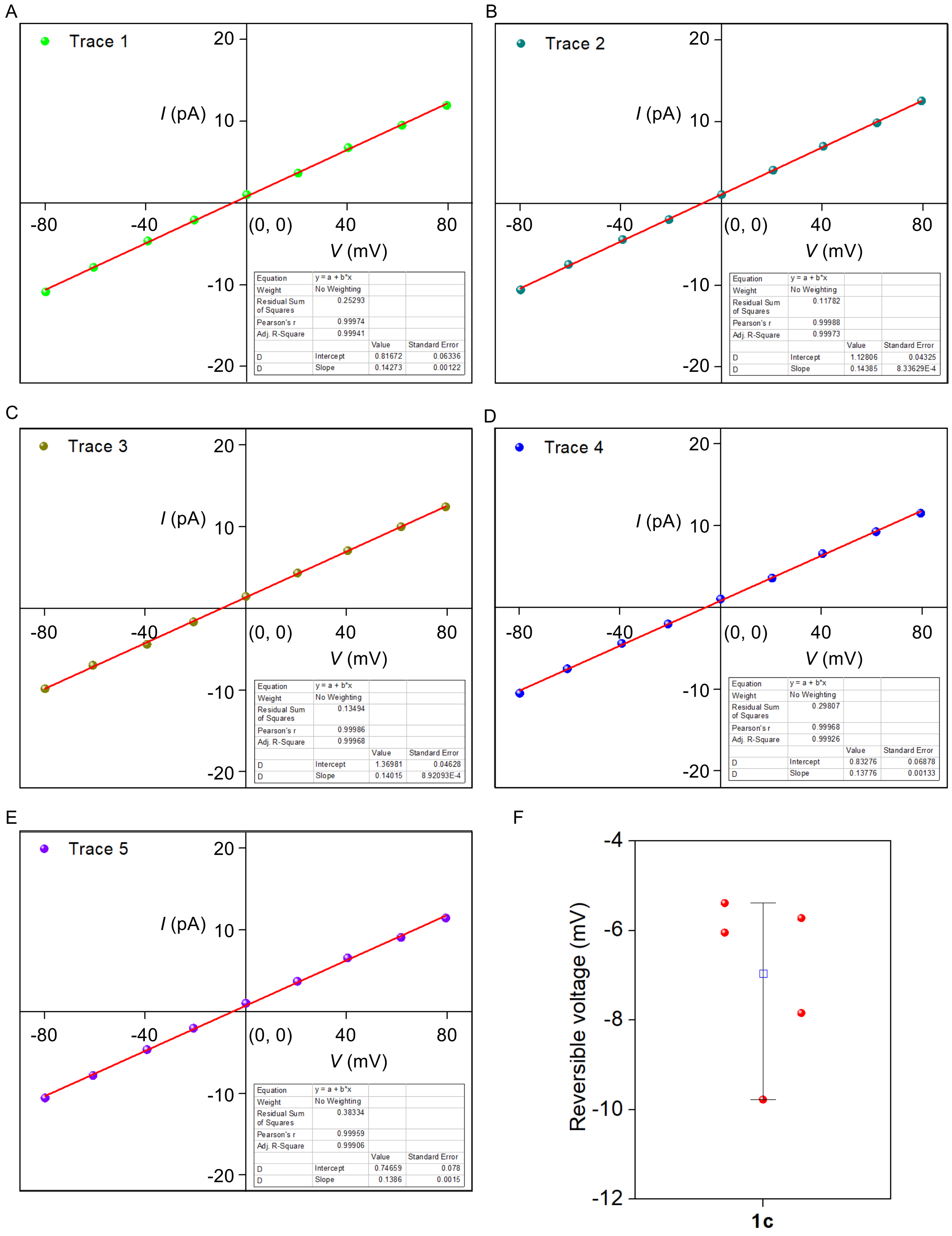

Fig. 33 All five traces of *I*–*V* plots of **1c** in unsymmetrical (cis/trans = 1 M/2 M) KCl solutions (A, B, C, D, E); box plot of the average reversible voltage obtained from five experiments (represented as red circles) (F).

The cis and trans chambers were filled with unsymmetrical KCl solutions. The cis chamber was filled with 1.0 M KCl solution, and the trans chamber was filled with 2 M KCl. The compound **1c** (5 μM) was added to the cis chamber and stirred for 15 min. The average reversal potential of −6.9 mV and permeability ratio $P_{Cl^-}/P_{K^+} = 5.0 \pm 1.3$ (Equation S6) were calculated.

The permeability ratio (P_{Cl^-}/P_{K^+}) was calculated by using Goldman-Hodgkin-Katz equation,

$$\frac{P_{Cl^-}}{P_{K^+}} = \frac{a_{K^+_{cis}} - a_{K^+_{trans}} \times exp\,(-\frac{V_{rev}F}{RT})}{a_{Cl^-_{cis}} \times exp\,(-\frac{V_{rev}F}{RT}) - a_{Cl^-_{trans}}} \quad \text{Equation S6}$$

where, P_{Cl^-}/P_{K^+} = anion/cation permeability ratio; a_{K^+cis} = K^+ activity in the cis chamber; a_{K^+trans} = K^+ activity in the trans chamber; a_{Cl^-cis} = Cl^- activity in the cis chamber; a_{Cl^-trans} = Cl^- activity in the trans chamber; V_{rev} = calculated reversal potential; F = Faraday constant; R = gas constant; T = temperature (K).

Table S2: Table for experimentally calculated permeability ratio of compound **1c**.

Entry	Reversible Voltage (V_r)	P_{Cl^-}/P_{K^+}
**1**	− 5.72213 mV	3.926
**2**	− 7.84192 mV	5.457
**3**	− 9.77389 mV	7.776
**4**	− 6.04500 mV	4.117
**5**	− 5.38665 mV	3.741
	Average = − 6.95 mV	Average = 5.003

XI. GSH-based Release of the Transport Active Ion Channel:

A. ^{1}H NMR experiment:

The generation of the transport active ion channel **1c** from transport inactive protransporter **1c'** was investigated by ^{1}H NMR experiment.

Assay details: A 2 mM sample of **1c'** was dissolved in DMSO-d_6, and ^{1}H NMR was collected on a 400 MHz Bruker NMR instrument at 25 °C. The sample was sequentially treated with 0.5 equiv. and 1 equiv. of GSH and ^{1}H NMR were recorded at 25 °C after each addition of GSH.

The addition of the 2 equiv. of GSH resulted in the disappearance of H_a' peak, and the appearance of H_a peak. Alongside, the H_b', H_c', H_d', and H_e' peaks shifted to H_b, H_c, H_d, and H_e peaks, respectively, which perfectly correlates with the ^{1}H NMR spectra of **1c**. This data demonstrated that the addition of GSH leads to the generation of compound **1c** from compound **1c'**.

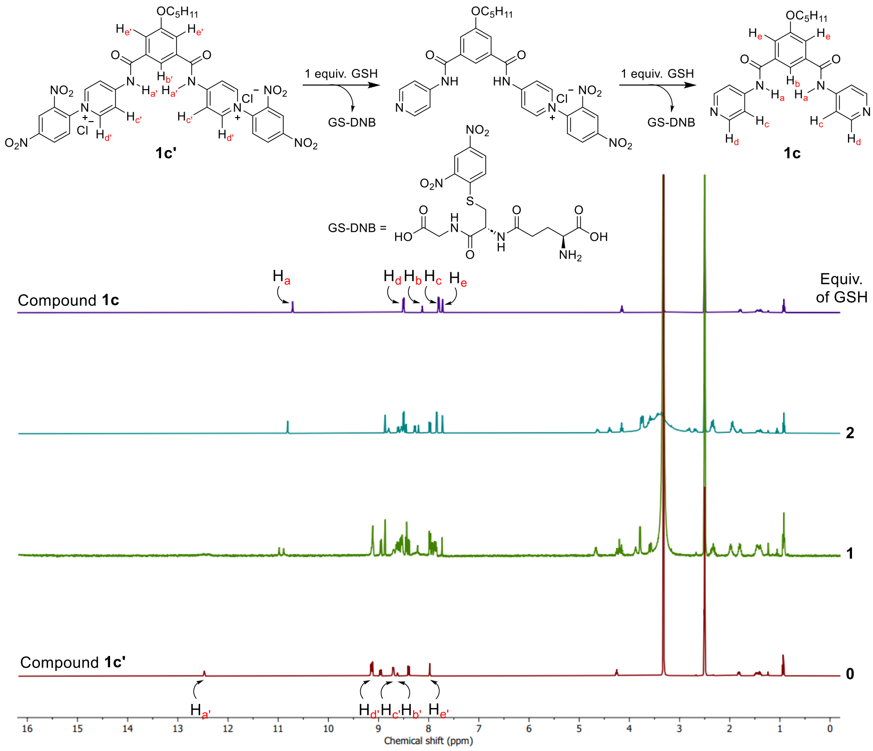

Fig. S34 Release of the transport active ion channel **1c** from the transport inactive protransporter **1c'** by reacting it with GSH.

B. Evidence of formation of the side product GS-DNB:

To distinguish the formation of the side product GS-DNB, during the release of the active channel forming molecule **1c** from protransporter **1c'**, HRMS data was collected in +ve mode. A distinct peak corresponding to the GS-DNB was observed in the HRMS spectra, validating the formation of the side product.

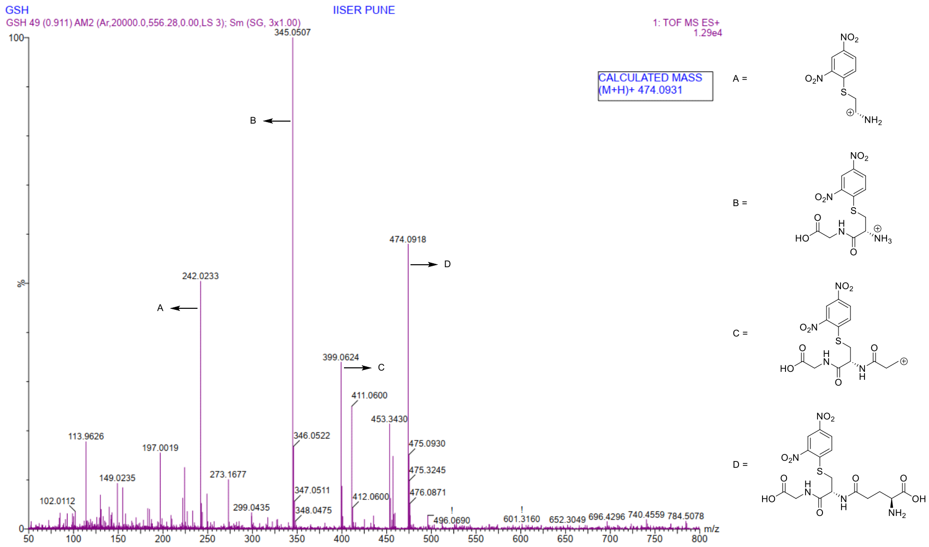

Fig. S35 HRMS spectrum of the side product GS-DNB.

C. GSH-triggered activation of ion transport:

Electrophysiology experiment:

Assay details: Diphytanoylphosphatidylcholine dissolved in n-decane (20 mg/mL) was used to form a Bilayer lipid membrane (BLM) across an aperture of 150 µM diameter in a polystyrene cup. Both cis and trans chambers were filled with symmetrical unbuffered 1 M KCl solution. The cis chamber was connected to the BC 535 head stage by an Ag-AgCl electrode, and the trans chamber was held at virtual ground. Compound **1c**' was added to the cis chamber (working concentration 5 µM), and the solution was stirred with a magnetic stirrer for 10 min, and the opening-closing event was monitored before and after the addition of 3 equiv. of GSH. Currents were low pass filtered at 1 kHz using pClamp9 software and an analog-to-digital converter (Digidata 1440, Molecular Probes). All data were analyzed using the software pClamp 10.7.

The addition of compound **1c'** did not show any opening-closing event at different holding potentials up to 2-3 h, confirming that **1c'** is not forming any ion channel inside the bilayer membrane. On the other hand, the addition of 2.5 equiv. of GSH on the membrane-embedded **1c'** rapidly triggered the current flow with open-close transition events at different holding potentials. This data confirmed that the uncaged **1c** (generated from protransporter **1c'**) could form the ion channels inside the bilayer membrane.

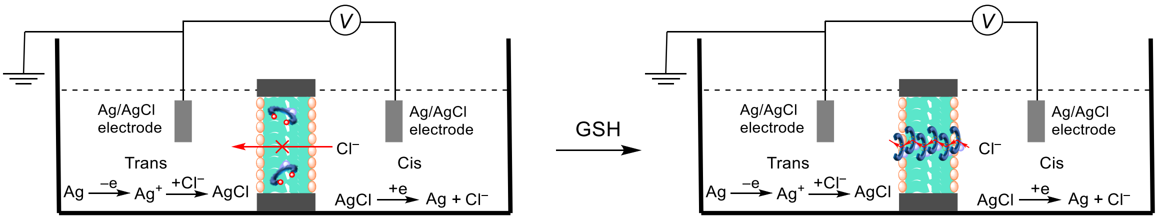

Fig. S36 Schematic illustration of assay details of the GSH-triggered release of active ion channel **1c** from the transport inactive protransporter **1c'** during the BLM experiment.

D. ^{1}H NMR experiment for DNB group cleavage with $Na_2S_2O_4$ as reducing agent:

Assay details: A 2 mM sample of **1c'** was dissolved in DMSO-d_6:H_2O (90:10), and ^{1}H NMR was collected on a 400 MHz Bruker NMR instrument at 25 °C. The sample was sequentially treated with 1 equiv. and 2 equiv. of $Na_2S_2O_4$ and ^{1}H NMR were recorded at 25 °C after each addition of $Na_2S_2O_4$. The addition of the 2 equiv. of $Na_2S_2O_4$ resulted in an insignificant change in the ^{1}H NMR spectra, validating that $Na_2S_2O_4$ cannot cleave the DNB groups from compound **1c'**.

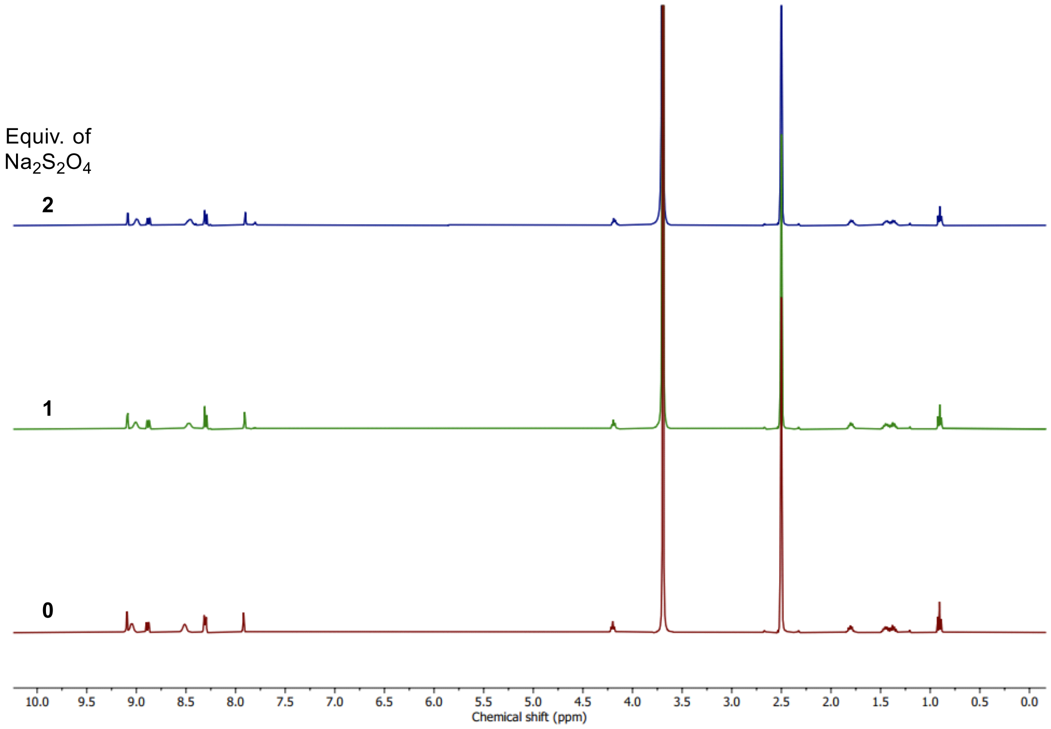

Fig. S37 Change in the ^{1}H NMR of compound **1c'** (2 mM) in DMSO–d_6:D_2O (90:10) at 25 °C upon sequential addition of $Na_2S_2O_4$ as a reducing agent.

XII. Single Crystal X-Ray Diffraction Study:

The single crystals of **1c** were grown from MeOH, allowing for the slow evaporation of the solvents over time. X-ray diffraction data on single crystals of **1c** were collected on a Bruker D8 Venture Duo X-ray diffractometer equipped with a Microfocus X-ray source (operated at 50 W; 50 kV/1 mA) and graded multilayer optics for monochromatic Mo Kα radiation (λ = 0.71073 Å) with a focused X-ray beam and a Photon 100 CMOS chip-based detector system at 150 K. The crystal was mounted on nylon Cryo Loops (Hampton Research) with Paraton-N (Hampton Research). The data integration and reduction were processed with SAINT software.1 A multi-scan absorption correction was applied to the collected reflections. The structure was solved by a direct method using SHELXTL3,4 and refined on F2 with a full-matrix least-squares technique using the SHELXL5 program package. All of the hydrogen atoms were refined anisotropically. All hydrogen atoms were located in successive difference Fourier maps and treated as riding atoms using SHELXL default parameters. The structures were examined using the Adsym subroutine in PLATON to ensure that no additional symmetries could be applied to the models.

Table S3: Details of the crystal structure data of Compound_1c.

Identification code	Compound_1c
CCDC number	2454559
Empirical formula	$C_{23}H_{26}N_4O_4$
Formula weight	422.48
Temperature	150(2) K
Crystal system	Monoclinic
Space group	P 2_1/c
Unit cell dimensions	a = 12.2023(11) Å α = 90°
	b = 10.9757(10) Å β = 105.485(2)°
	c = 16.4527(13) Å γ = 90°
Volume	2123.5(3) $Å^3$
Z	4
Density (calculated)	1.321 g/cm^3
Absorption coefficient	0.092 mm^{-1}
F(000)	896.0
Crystal size	0.7 x 0.4 x 0.3 mm^3
Radiation	MoKα (λ = 0.71073)
Theta range for data collection	4.514 to 50.05°.
Index ranges	$-14 \leq h \leq 14$, $-13 \leq k \leq 13$, $-19 \leq l \leq 17$
Reflections collected	53321
Independent reflections	3749 [R_{int} = 0.1319, R_{sigma} = 0.0539]
Data/restraints/parameters	3749/0/284
Goodness-of-fit on F^2	1.053
Final R indexes [I>=2σ (I)]	R_1 = 0.0527, wR_2 = 0.1234
Final R indexes [all data]	R_1 = 0.0721, wR_2 = 0.1334
Largest diff. peak and hole	0.25 and -0.28 e.$Å^{-3}$

Table S4. Fractional Atomic Coordinates (×10^4) and Equivalent Isotropic Displacement Parameters ($Å^2$ × 10^3) for Compond_1c. U_{eq} is defined as 1/3 of the trace of the orthogonalized U_{IJ} tensor.

Atom	x	y	z	U(eq)
O4	7476.8(13)	6159.5(14)	6618.5(9)	29.1(4)
N1	-510.6(17)	-146.2(18)	2694.7(12)	33.0(5)
C1	82.1(19)	686(2)	2399.5(14)	29.1(5)
O1	3043.9(14)	108.5(15)	5303.4(10)	35.8(4)

N2	2629.3(15)	1248.2(16)	4109.4(10)	22.9(4)
C2	1108.7(18)	1152(2)	2852.4(13)	25.5(5)
O2	6838.2(12)	4950.5(13)	5047.0(8)	21.9(4)
N3	5461.3(14)	4459.8(15)	3859.6(9)	15.8(4)
C3	1590.4(17)	746.0(19)	3671.8(13)	21.4(5)
O3	6786.3(12)	1366.8(13)	7027.5(9)	26.6(4)
N4	5453.4(15)	7397.6(15)	2220.6(10)	19.3(4)
C4	989(2)	-116(2)	3997.5(15)	31.5(6)
C5	-42(2)	-517(2)	3483.5(15)	36.6(6)
C6	3305.5(17)	905.4(19)	4878.9(13)	19.8(5)
C7	4415.9(17)	1579.5(18)	5176.9(12)	16.0(4)
C8	5148.8(17)	1190.8(19)	5922.5(12)	18.8(5)
C9	6168.1(17)	1799.2(18)	6269.0(12)	18.1(5)
C10	6470.8(17)	2780.3(18)	5844.2(12)	18.4(5)
C11	5739.0(16)	3169.1(18)	5087.3(11)	14.7(4)
C12	4705.0(17)	2576.8(18)	4760.4(12)	16.3(4)
C13	6074.5(17)	4271.0(17)	4671.0(12)	15.9(4)
C14	5499.8(17)	5468.1(18)	3347.6(12)	14.6(4)
C15	4620.8(17)	5584.7(18)	2609.6(12)	17.1(4)
C16	4640.8(18)	6543.4(19)	2076.9(12)	19.6(5)
C17	6280.9(18)	7275.5(19)	2940.6(12)	20.8(5)
C18	6353.0(17)	6344.5(18)	3513.0(12)	17.2(5)
C19	7774.1(18)	2036(2)	7474.5(13)	24.2(5)
C20	8099.6(18)	1570(2)	8369.8(13)	24.3(5)
C21	9241.2(19)	2061(2)	8870.4(13)	27.4(5)
C22	9536.7(19)	1726(2)	9799.9(13)	27.9(5)
C23	10783(2)	1954(3)	10235.8(14)	36.2(6)

Table S5. Anisotropic Displacement Parameters (Å^2×10^3) for Compound_1c. The Anisotropic displacement factor exponent takes the form: $-2\pi^2[h^2a^{*2}U_{11}+2hka^*b^*U_{12}+\ldots]$.

Atom	U_{11}	U_{22}	U_{33}	U_{23}	U_{13}	U_{12}
O4	24.2(9)	30.7(9)	25.6(8)	-6.5(7)	-5.3(7)	10.7(7)
N1	23.4(11)	37.3(12)	33.9(11)	-2.0(9)	0.0(9)	-7.7(9)

C1	24.9(12)	36.2(14)	23.4(12)	-1.6(10)	1.6(10)	-2.9(11)
O1	27.9(9)	40.8(10)	32.4(9)	15.1(8)	-3.0(7)	-14.4(8)
N2	21.5(10)	26.8(10)	17.9(9)	4.7(8)	1.0(8)	-8.8(8)
C2	22.6(12)	30.2(13)	22.5(11)	-1.6(10)	4.2(10)	-6.8(10)
O2	23.8(8)	21.8(8)	17.2(7)	-0.5(6)	0.5(6)	-7.3(7)
N3	17.9(9)	14.3(9)	12.7(8)	-1.0(7)	-0.3(7)	-4.3(7)
C3	17.1(11)	22.0(12)	23.3(11)	-4.2(9)	2.0(9)	-2.9(9)
O3	22.7(8)	29.1(9)	21.0(8)	11.0(7)	-6.4(6)	-6.8(7)
N4	23.7(10)	16.8(9)	17.7(9)	1.3(7)	6.2(8)	0.2(8)
C4	28.1(13)	32.9(13)	29.2(13)	5.7(10)	-0.1(11)	-8.4(11)
C5	28.9(14)	38.9(15)	37.8(14)	5.4(12)	1.7(11)	-13.4(12)
C6	19.4(11)	19.2(11)	20.3(11)	1.4(9)	4.5(9)	-0.8(9)
C7	16.0(11)	16.5(10)	15.9(10)	-2.2(8)	4.6(8)	0.2(8)
C8	21.5(11)	17.2(11)	18.3(10)	1.9(8)	6.2(9)	-0.8(9)
C9	18.9(11)	18.7(11)	14.2(10)	2.7(8)	-0.1(8)	1.7(9)
C10	17.4(11)	19.1(11)	17.1(10)	-1.0(8)	2.0(9)	-0.5(9)
C11	16.6(10)	15.7(10)	12.5(9)	-2.5(8)	5.3(8)	2.0(8)
C12	17.5(11)	17.6(11)	12.8(10)	-1.8(8)	2.1(8)	2.3(9)
C13	18.0(11)	15.1(11)	14.1(10)	-2.0(8)	3.6(8)	3.1(9)
C14	18.1(11)	13.5(10)	13.4(10)	-0.4(8)	6.2(8)	2.5(8)
C15	17.4(11)	16.7(10)	15.9(10)	-2.1(8)	2.2(8)	-1.5(9)
C16	24.1(12)	19.8(11)	13.4(10)	-0.4(8)	2.2(9)	5.6(9)
C17	23.4(12)	19.3(11)	19.7(11)	-0.2(9)	5.7(9)	-2.8(9)
C18	16.3(11)	18.6(11)	15.7(10)	1.5(8)	2.5(8)	0.1(9)
C19	19.3(11)	27.5(12)	21.8(11)	3.7(9)	-1.2(9)	-6.1(10)
C20	20.3(12)	28.2(12)	21.4(11)	5.5(9)	0.4(9)	-1.5(10)
C21	22.2(12)	33.6(13)	23.9(12)	2.5(10)	2.0(10)	-1.6(10)
C22	26.6(13)	32.3(13)	22.1(11)	1.4(10)	1.7(10)	2.8(10)

C23	27.9(13)	53.2(17)	22.7(12)	-3.1(11)	-1.8(10)	2.5(12)

Table S6. Bond lengths [Å] for Compound_1c.

Atom	Atom	Length (Å)	Atom	Atom	Length (Å)
N1	C1	1.334(3)	C6	C7	1.506(3)
N1	C5	1.334(3)	C7	C8	1.379(3)
C1	C2	1.374(3)	C7	C12	1.386(3)
O1	C6	1.214(2)	C8	C9	1.393(3)
N2	C3	1.393(3)	C9	C10	1.386(3)
N2	C6	1.367(3)	C10	C11	1.392(3)
C2	C3	1.392(3)	C11	C12	1.393(3)
O2	C13	1.225(2)	C11	C13	1.500(3)
N3	C13	1.361(2)	C14	C15	1.395(3)
N3	C14	1.399(2)	C14	C18	1.390(3)
C3	C4	1.389(3)	C15	C16	1.374(3)
O3	C9	1.360(2)	C17	C18	1.376(3)
O3	C19	1.435(2)	C19	C20	1.509(3)
N4	C16	1.339(3)	C20	C21	1.515(3)
N4	C17	1.342(3)	C21	C22	1.520(3)
C4	C5	1.388(3)	C22	C23	1.519(3)

Table S7. Bond angles for Compound_1c.

Atom	Atom	Atom	Angle (°)	Atom	Atom	Atom	Angle (°)
C1	N1	C5	115.5(2)	C10	C9	C8	119.68(18)
N1	C1	C2	124.2(2)	C9	C10	C11	119.70(19)

C6	N2	C3	127.64(18)	C10	C11	C12	120.06(18)
C1	C2	C3	119.6(2)	C10	C11	C13	117.89(18)
C13	N3	C14	127.81(17)	C12	C11	C13	121.97(17)
C2	C3	N2	117.66(19)	C7	C12	C11	120.09(18)
C4	C3	N2	124.84(19)	O2	C13	N3	123.22(18)
C4	C3	C2	117.48(19)	O2	C13	C11	121.61(17)
C9	O3	C19	118.42(16)	N3	C13	C11	115.16(17)
C16	N4	C17	115.66(17)	C15	C14	N3	117.07(17)
C5	C4	C3	117.9(2)	C18	C14	N3	124.99(17)
N1	C5	C4	125.4(2)	C18	C14	C15	117.94(18)
O1	C6	N2	123.02(19)	C16	C15	C14	119.02(19)
O1	C6	C7	121.23(18)	N4	C16	C15	124.20(19)
N2	C6	C7	115.75(18)	N4	C17	C18	124.99(19)
C8	C7	C6	116.81(18)	C17	C18	C14	118.18(19)
C8	C7	C12	119.60(19)	O3	C19	C20	107.03(17)
C12	C7	C6	123.54(18)	C19	C20	C21	111.97(18)
C7	C8	C9	120.80(19)	C20	C21	C22	113.42(19)
O3	C9	C8	115.10(18)	C23	C22	C21	111.96(19)

Table S8. Torsion Angles for Compound_1c.

A	B	C	D	Angle/°	A	B	C	D	Angle/°
N1	C1	C2	C3	0.4(4)	C8	C7	C12	C11	1.1(3)
C1	N1	C5	C4	-0.5(4)	C8	C9	C10	C11	1.8(3)
C1	C2	C3	N2	-179.7(2)	C9	O3	C19	C20	164.88(18)
C1	C2	C3	C4	-0.7(3)	C9	C10	C11	C12	0.2(3)
O1	C6	C7	C8	4.4(3)	C9	C10	C11	C13	177.07(17)
O1	C6	C7	C12	-173.2(2)	C10	C11	C12	C7	-1.7(3)

N2	C3	C4	C5	179.3(2)	C10	C11	C13	O2	-14.0(3)
N2	C6	C7	C8	-175.45(18)	C10	C11	C13	N3	166.90(17)
N2	C6	C7	C12	7.0(3)	C12	C7	C8	C9	1.0(3)
C2	C3	C4	C5	0.4(3)	C12	C11	C13	O2	162.81(19)
N3	C14	C15	C16	-178.28(17)	C12	C11	C13	N3	-16.3(3)
N3	C14	C18	C17	178.99(18)	C13	N3	C14	C15	-166.01(19)
C3	N2	C6	O1	-2.7(4)	C13	N3	C14	C18	15.1(3)
C3	N2	C6	C7	177.09(19)	C13	C11	C12	C7	-178.37(17)
C3	C4	C5	N1	0.2(4)	C14	N3	C13	O2	-6.4(3)
O3	C9	C10	C11	-177.16(19)	C14	N3	C13	C11	172.70(17)
O3	C19	C20	C21	170.79(18)	C14	C15	C16	N4	-0.6(3)
N4	C17	C18	C14	-1.1(3)	C15	C14	C18	C17	0.1(3)
C5	N1	C1	C2	0.2(4)	C16	N4	C17	C18	1.2(3)
C6	N2	C3	C2	-173.0(2)	C17	N4	C16	C15	-0.3(3)
C6	N2	C3	C4	8.2(4)	C18	C14	C15	C16	0.7(3)
C6	C7	C8	C9	-176.64(18)	C19	O3	C9	C8	-172.37(18)
C6	C7	C12	C11	178.51(18)	C19	O3	C9	C10	6.6(3)
C7	C8	C9	O3	176.63(18)	C19	C20	C21	C22	173.5(2)
C7	C8	C9	C10	-2.4(3)	C20	C21	C22	C23	166.0(2)

Table S9. Hydrogen Atom Coordinates (Å × 10^4) and Isotropic Displacement Parameters (Å^2 × 10^3) for Compound_1c.

Atom	x	y	z	U(eq)
H4A	7340	5692	6191	44
H4B	8062	5854	6958	44
H1	-225	974	1840	35
H2A	2879	1858	3861	27

H2	1487	1748	2608	31
H3	4983	3878	3629	19
H4	1275	-421	4554	38
H5	-449	-1104	3712	44
H8	4956	500	6203	23
H10	7175	3185	6069	22
H12	4197	2857	4251	20
H15	4017	5010	2477	20
H16	4040	6604	1574	24
H17	6861	7878	3065	25
H18	6969	6302	4007	21
H19A	7603	2918	7465	29
H19B	8405	1907	7210	29
H20A	8130	669	8363	29
H20B	7509	1812	8650	29
H21A	9237	2959	8817	33
H21B	9840	1741	8624	33
H22A	9060	2211	10081	33
H22B	9361	854	9856	33
H23A	11257	1441	9979	54
H23B	10933	1755	10836	54
H23C	10963	2814	10173	54

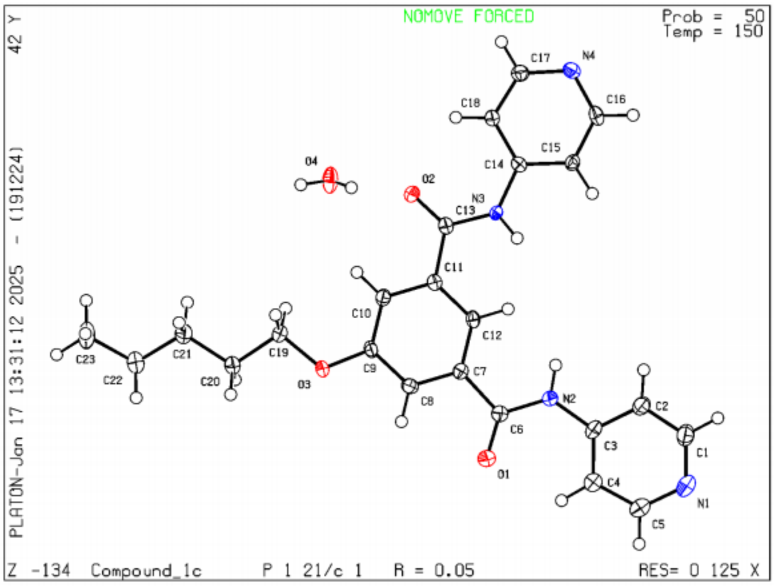

Fig. S38 ORTEP diagram of compound **1c**.

XIII. Theoretical Studies:

Computational details:

All geometry optimizations were carried out by using the Gaussian 09 package[S14] and the MOPAC 2016 package.[S15]

To envisage the disassembled structure of the protransporter **1c'**, geometry optimization of the **1c'** was carried out in the Gaussian 09 package using the B3LYP functional and 6-311++G(d,p) basis set. The geometry optimization yielded no imaginary frequencies, confirming that the optimized structure corresponds to the ground-state minimum. The geometry-optimized structure revealed that the 2,4-dinitrophenyl groups remain approximately perpendicular to the stacking plane. Therefore, restricting it to form a self-assembled barrel-rosette ion channel by forming the π-π stacking, H-bonding interactions. Hence, compound **1c'** is expected to remain disassembled in the membrane for the inadequate self-assembly process.

Moreover, to understand the feasible position of the Cl^- ion, the structure was optimized with the Cl^- ion in different positions (Fig. S38). Furthermore, we evaluate the Gibbs free energy change (ΔG) for the movement of one Cl^- ion from the vicinity of the positive charge to the anion binding pocket. We observed ΔG = −17.403 kcal/mol, indicating that one of the Cl^- ions is more preferred to remain in the ion binding pocket.

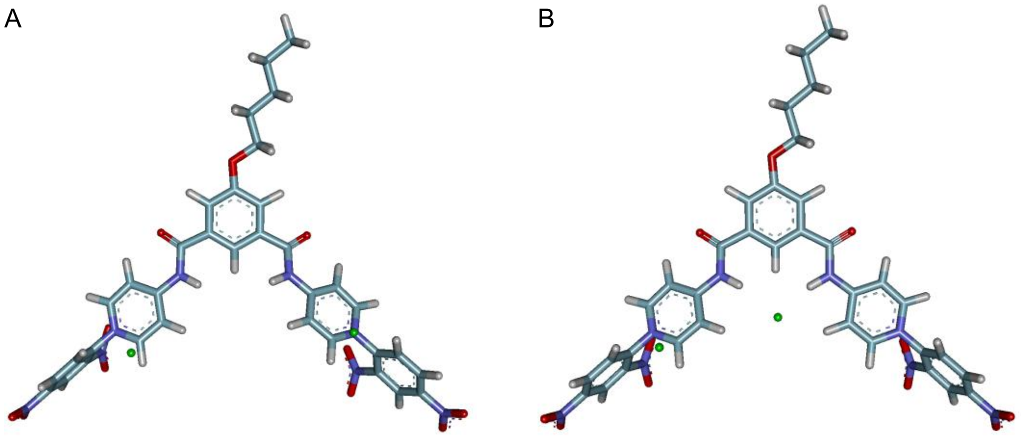

Fig. S39 Geometry optimized structure of protransporter **1c'** having Cl^- ion in a different position.

To understand the Cl^- ion binding with the monomeric unit of the channel forming molecule **1c**, geometry optimization of **1c** was carried out in the Gaussian 09 package using the B3LYP functional and 6-311++G(d,p) basis set. The geometry-optimized structure indicated that H_a, H_b, and H_c protons are involved in the Cl^- ion binding process. This data also supports the experimentally obtained NMR titration data.

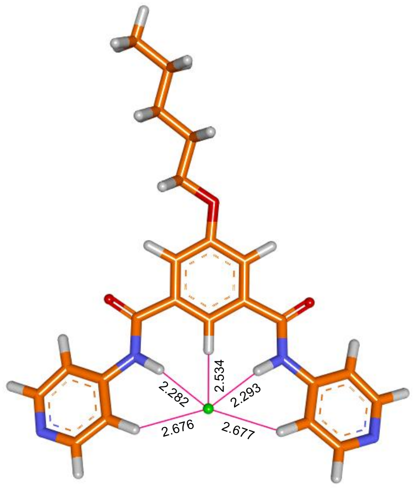

Fig. S40 Geometry optimized structure of channel forming molecule **1c** with the Cl^- ion.

Furthermore, to visualize the barrel-rosette channel formation, the dodecameric assembly of compound **1c** was generated from the monomeric unit of the crystal structure of compound **1c**. During the calculation, the pentyl chain was reduced to a methyl chain to minimize the time required for geometry optimization. The dodecameric assembly was then optimized for geometry using the MOPAC 2016 program package with the PM6-DH+ method. The optimized structure shows a prominent channel path for the translocation of the ions. The calculated average channel diameter is 3.6 Å.

A

B

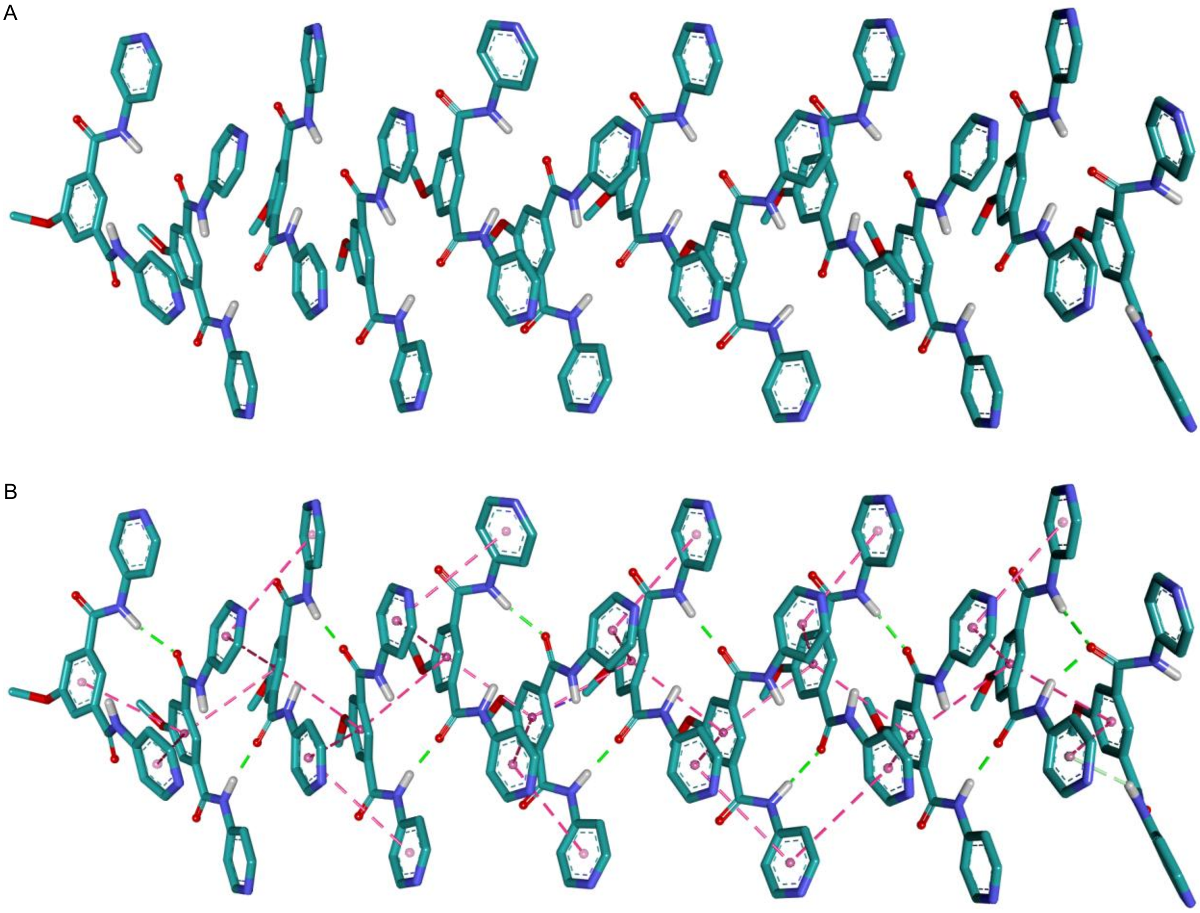

Fig. S41 Side view of geometry optimized channel assembly of $(\mathbf{1a})_{12}$ in the Licorice model (A), Hydrogen bonding, and π–π stacking interactions responsible for stabilization of the channel assembly of $(\mathbf{1a})_{12}$ (B). Only polar hydrogens are shown for better clarity in the self-assembled structure.

To evaluate the possible interactions between channel **1a** and Cl^- ion during transport through the channel cavity, $[(\mathbf{1a})_{12}+Cl^-]$ assembly was further optimized by using the MOPAC 2016 package with the PM6-DH+ method by keeping the Cl^- ion at different positions of the dodecameric assembly of the channel **1a**. The geometry-optimized data of $[(\mathbf{1a})_{12}+Cl^-]$ assembly indicated the Cl^- ion has interactions with amide N-H and aromatic C-H groups. These overall interactions between channel **1a** and Cl^- help the ion to translocate across the membrane.

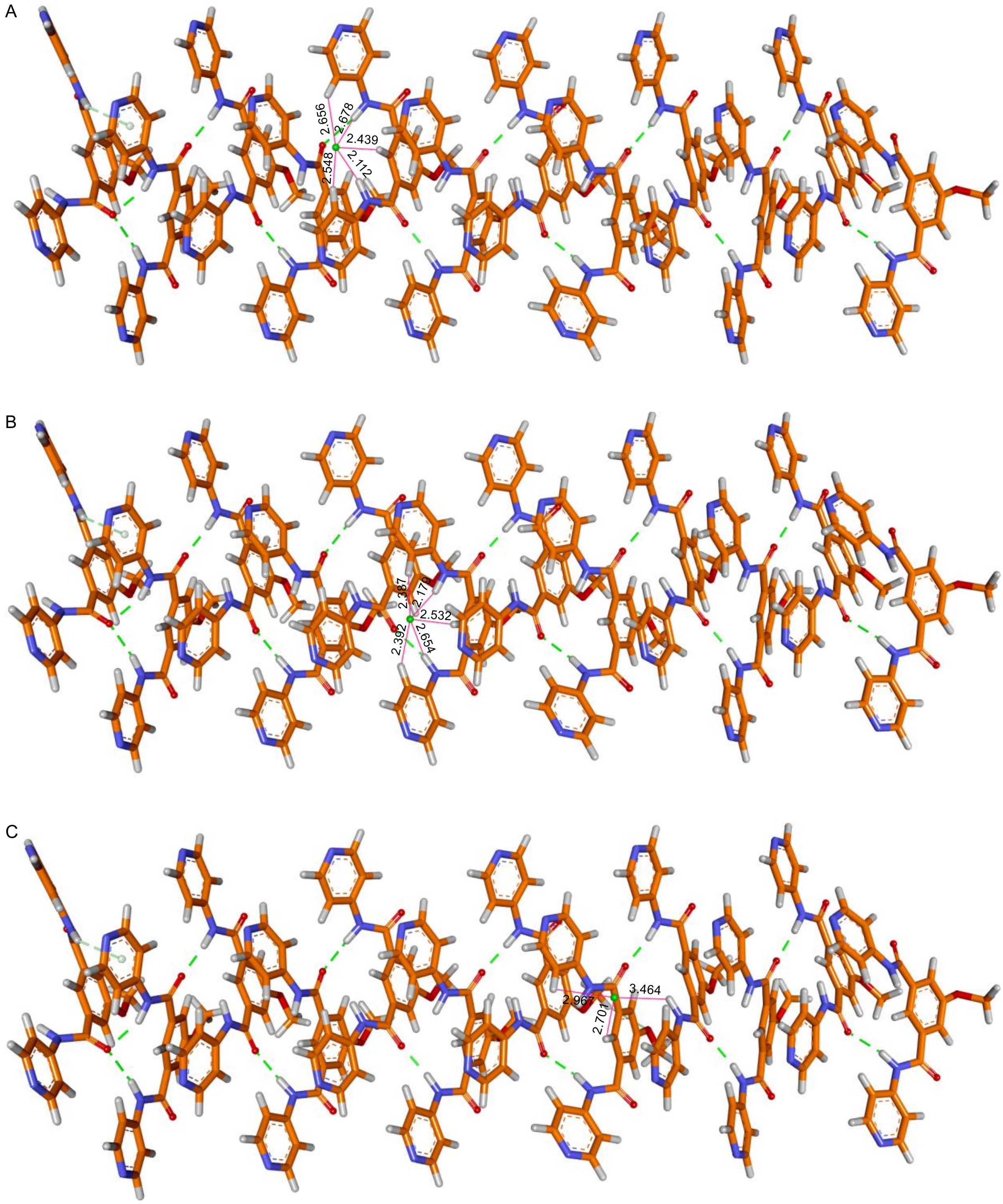

Fig. S42 Side view of geometry optimized [($\mathbf{1a}$)$_{12}$+Cl^{-}] assembly by varying Cl^{-} ion position (A-C).

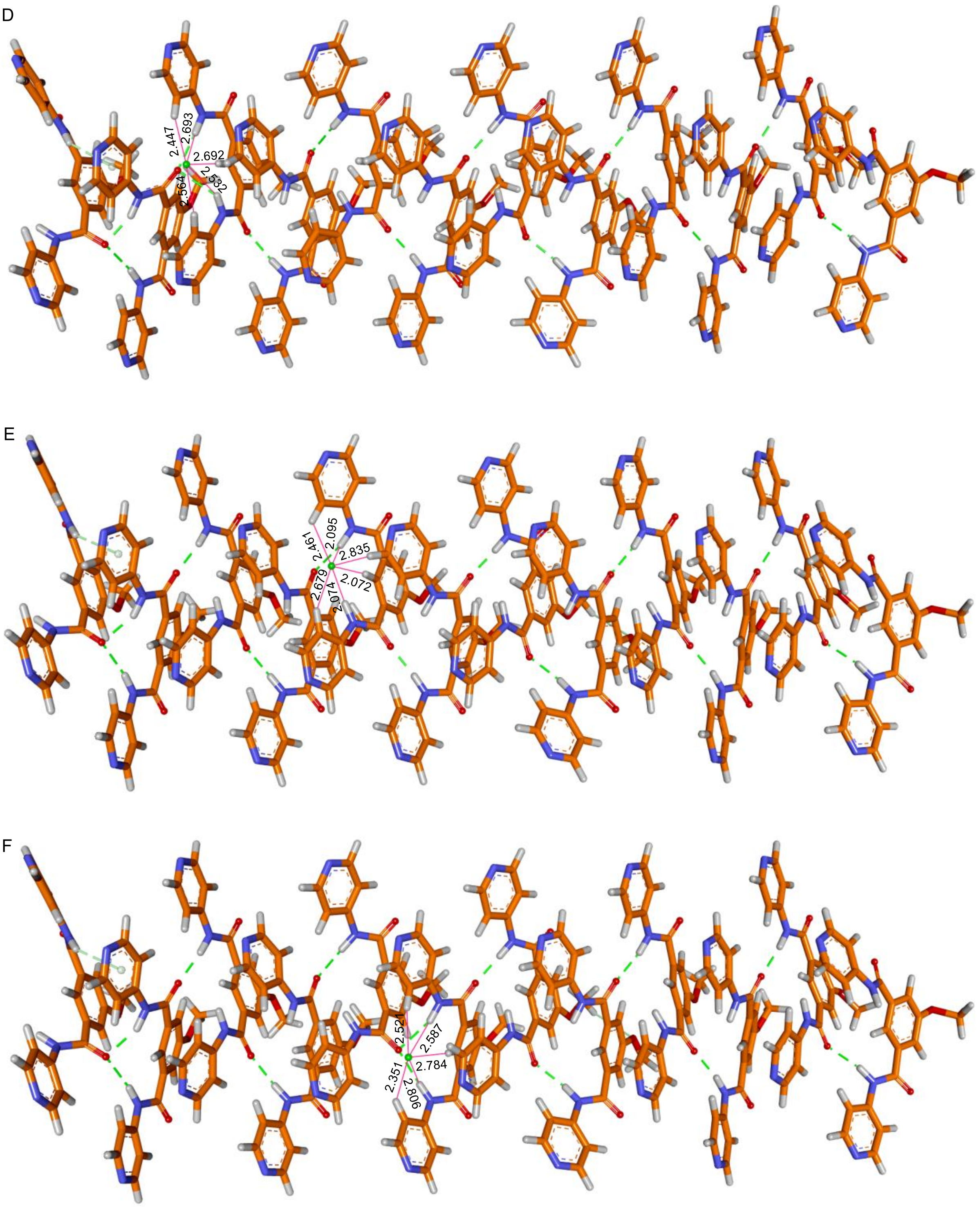

Fig. S43 Side view of geometry optimized [(**1a**)$_{12}$+Cl^{-}] assembly by varying Cl^{-} ion position (D-F).

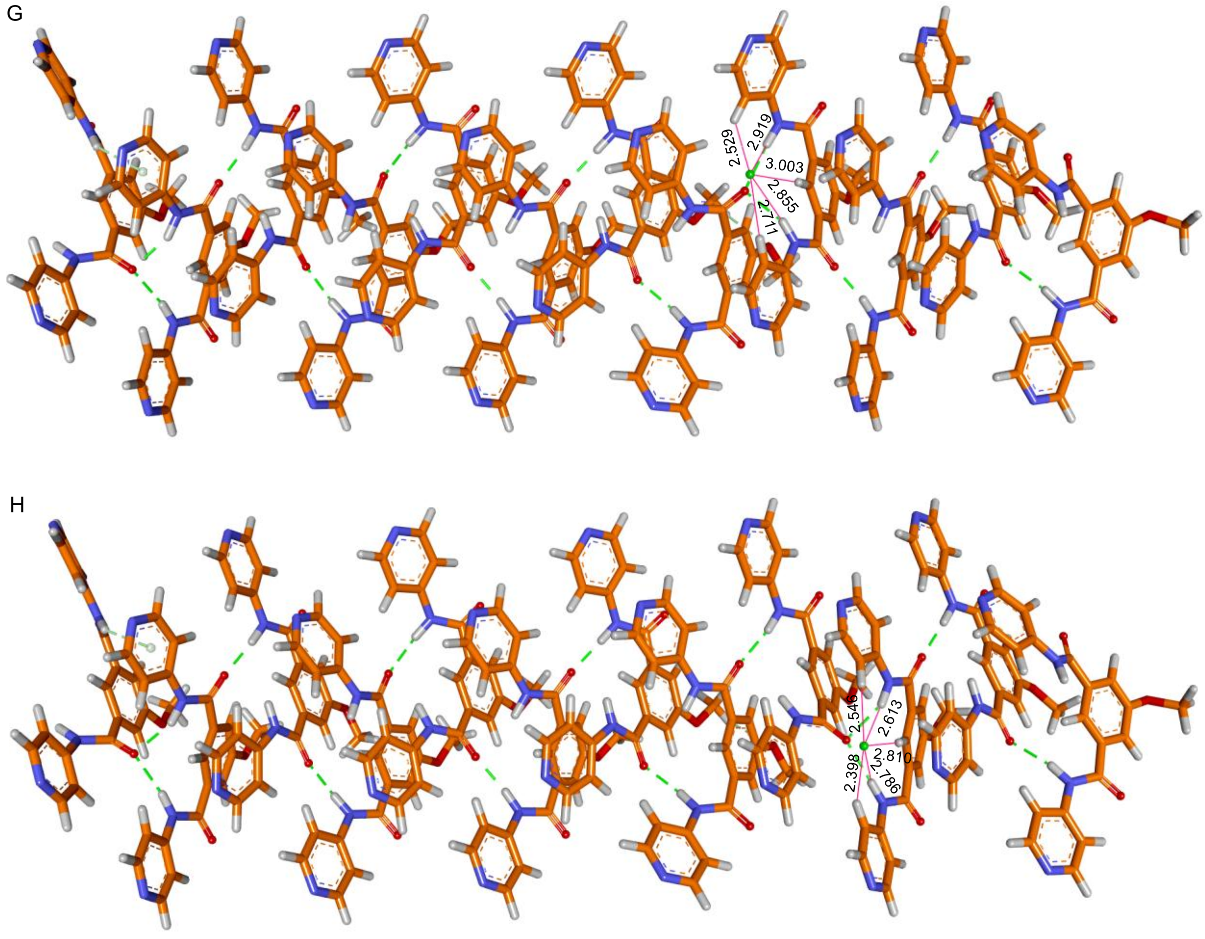

Fig. S44 Side view of geometry optimized [(**1a**)$_{12}$+Cl^{-}] assembly by varying Cl^{-} ion position (G, H).

XIV. NMR Data:

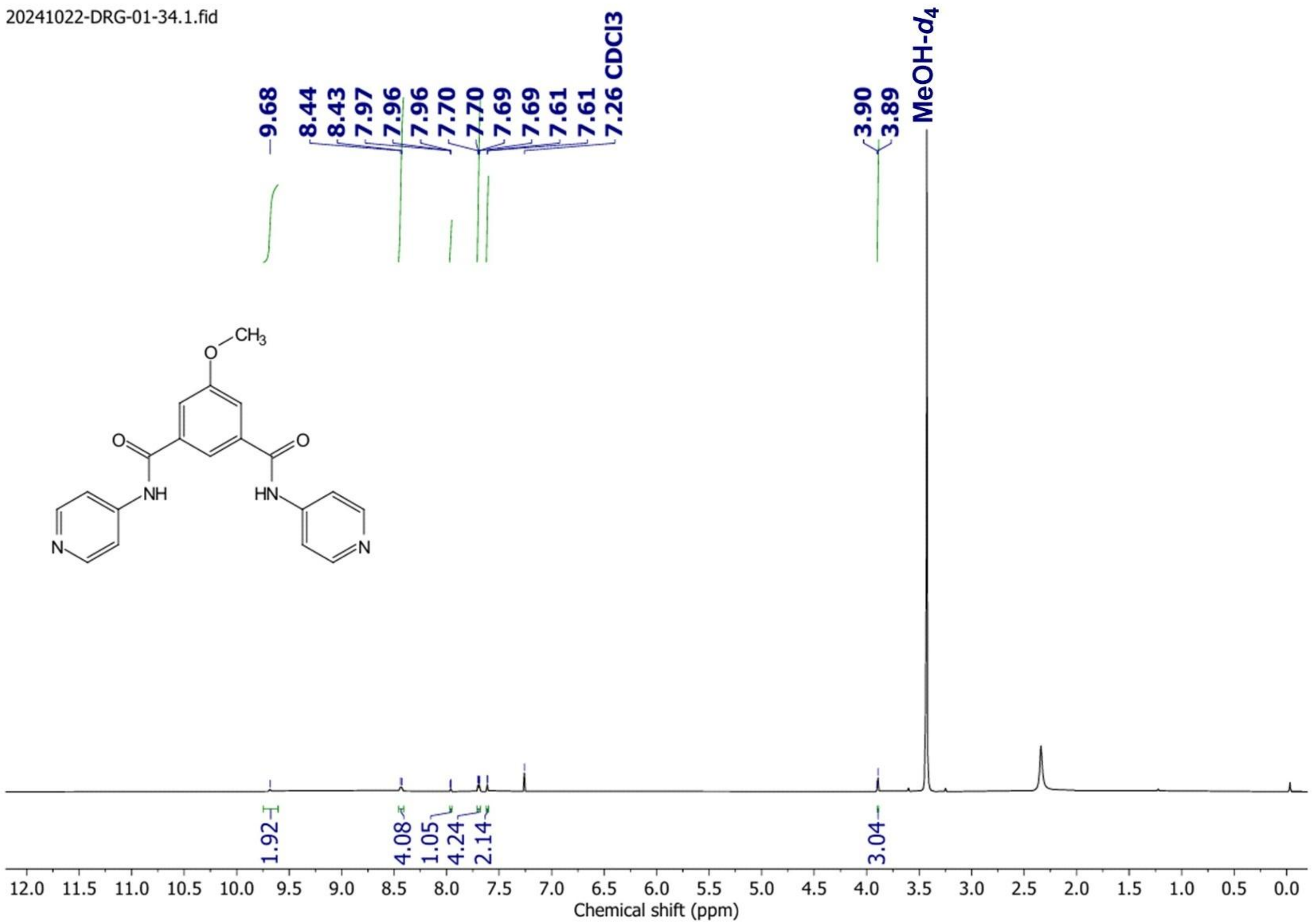

Fig. S45 ^{1}H NMR (400 MHz) spectrum of compound **1a** in 1:10 (v/v) MeOH-d_4:$CDCl_3$.

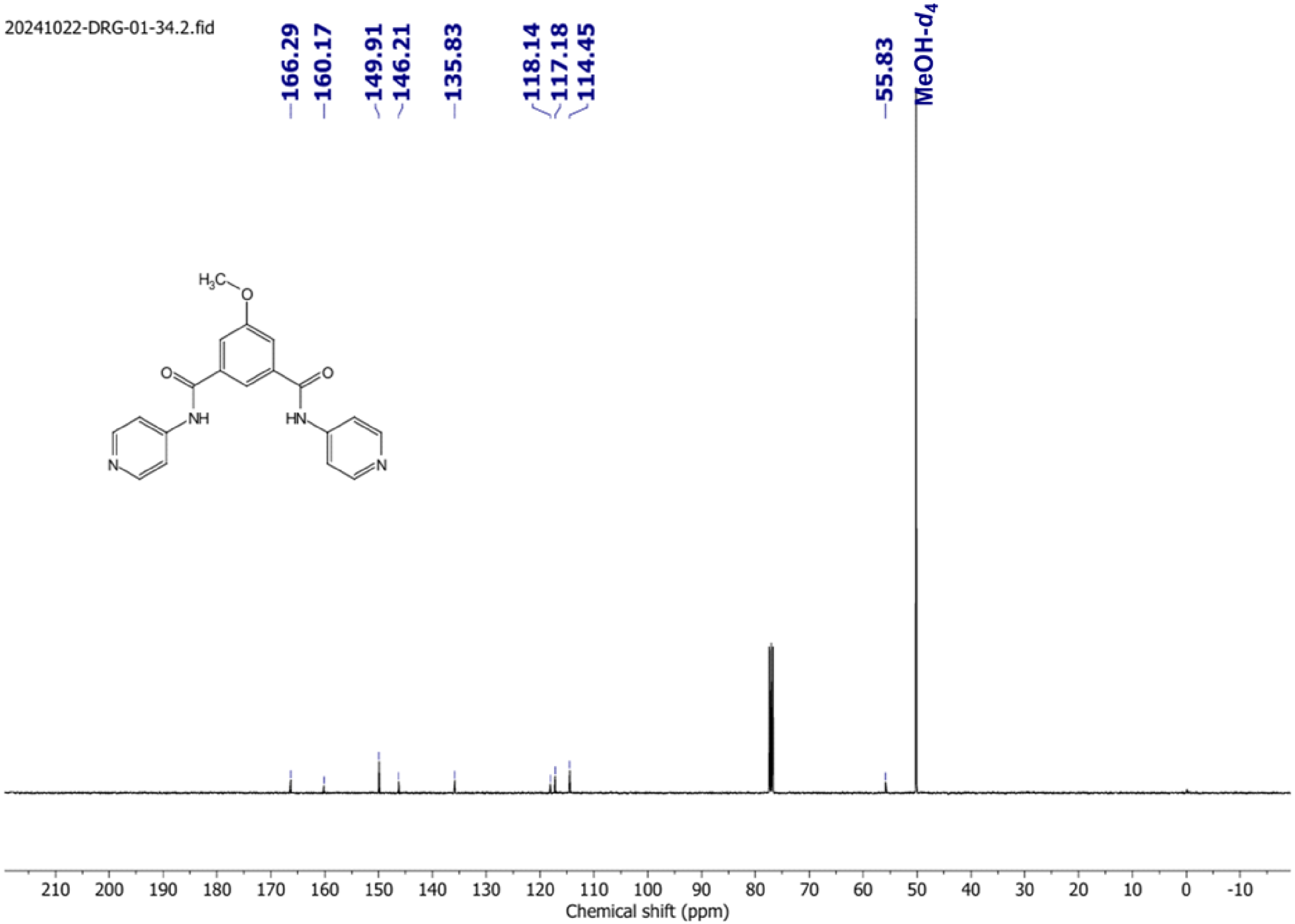

Fig. S46 ^{13}C NMR (101 MHz) spectrum of compound **1a** in 1:10 (v/v) MeOH-d_4:$CDCl_3$.

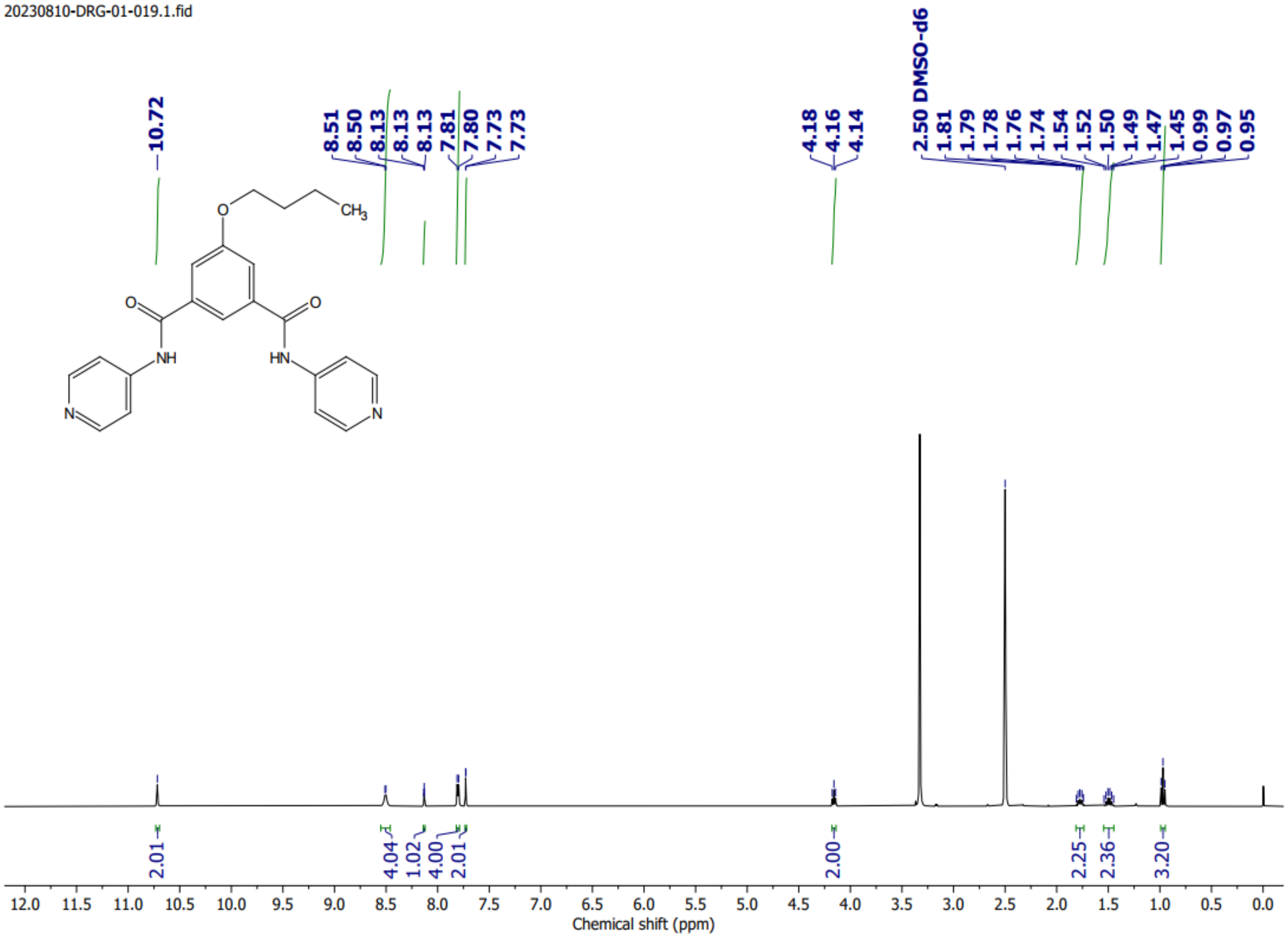

Fig. S47 ^{1}H NMR (400 MHz) spectrum of compound **1b** in DMSO-d_6.

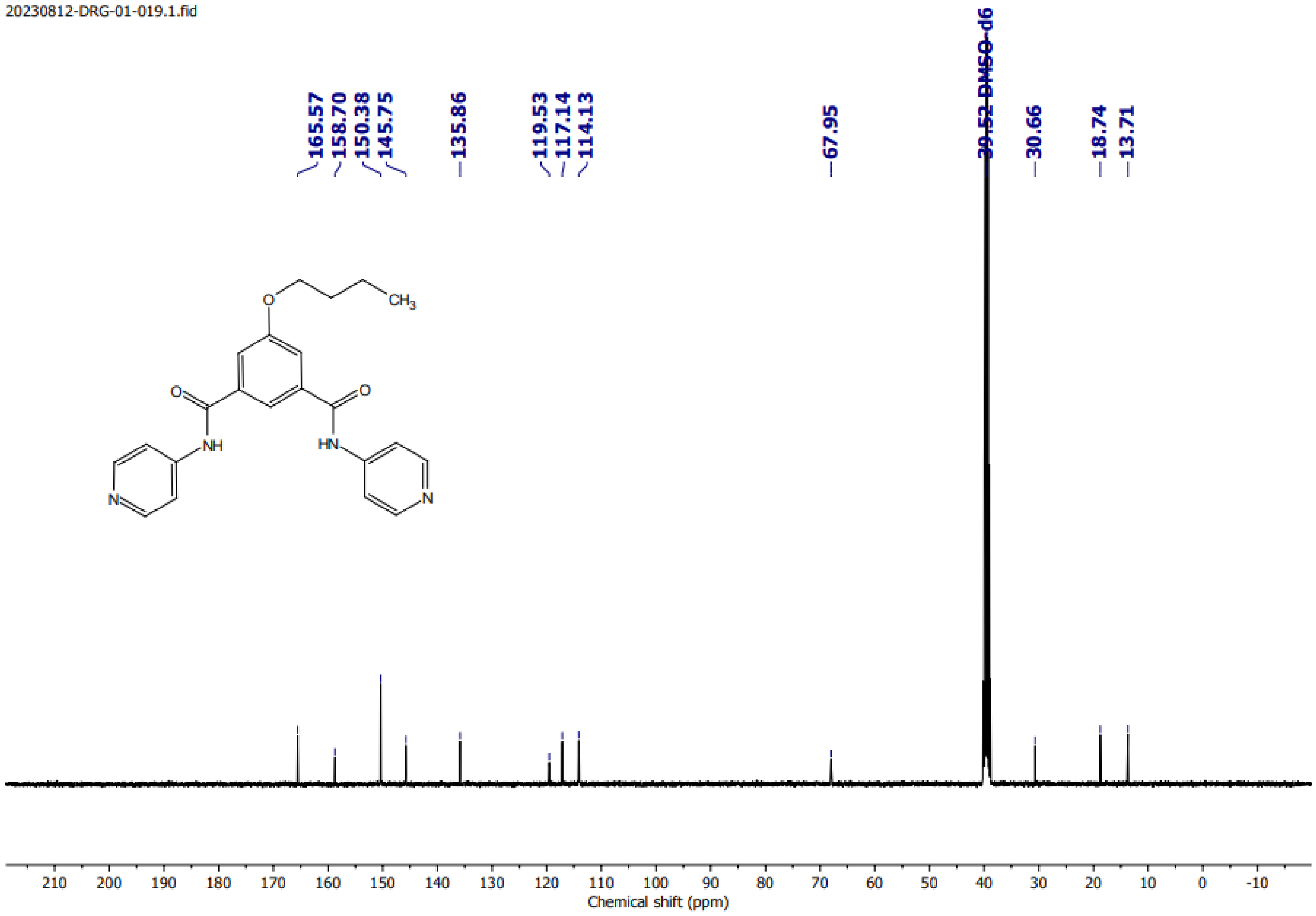

Fig. S48 ^{13}C NMR (101 MHz) spectrum of compound **1b** in DMSO-d_6.

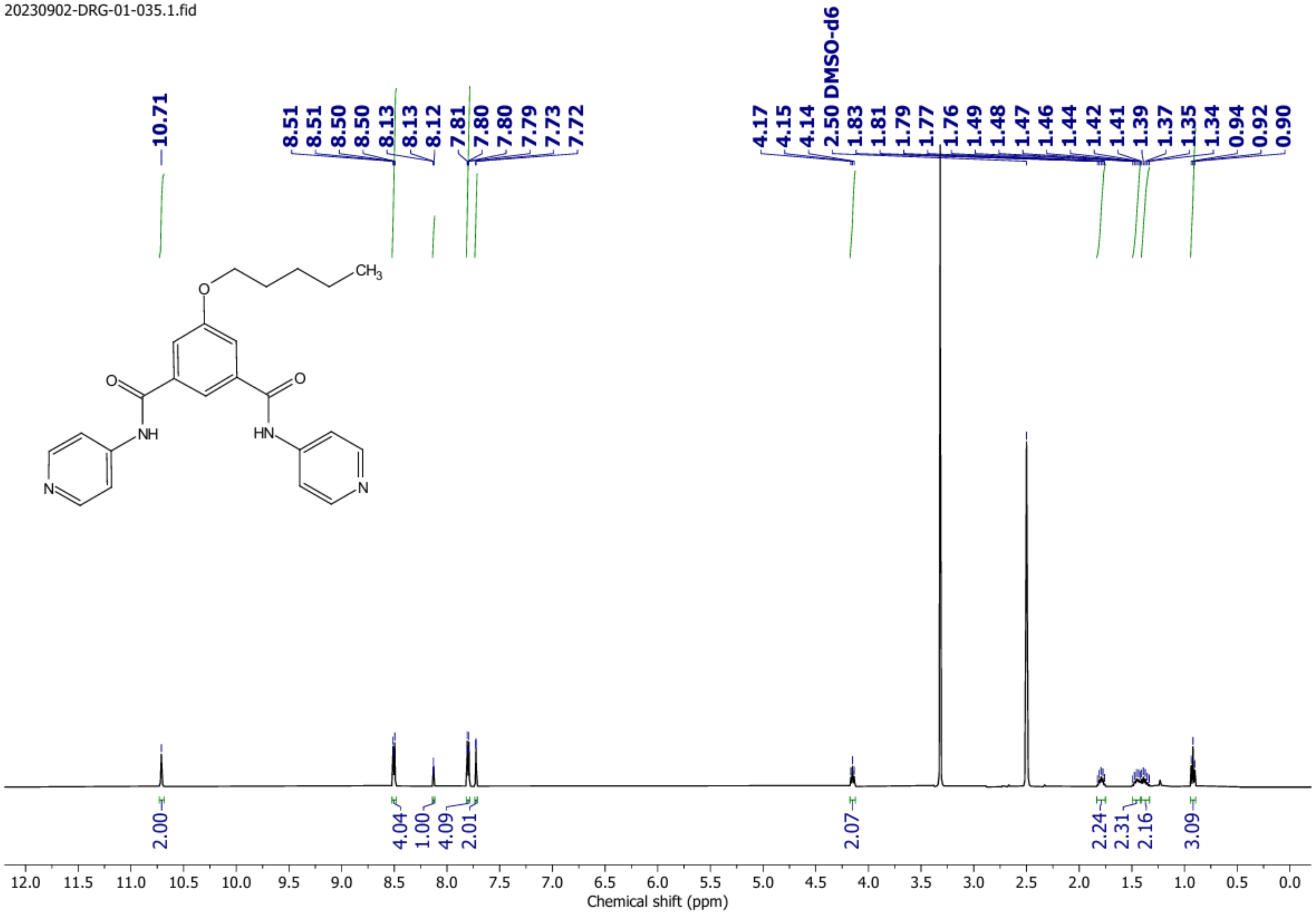

Fig. S49 ^{1}H NMR (400 MHz) spectrum of compound **1c** in DMSO-d_6.

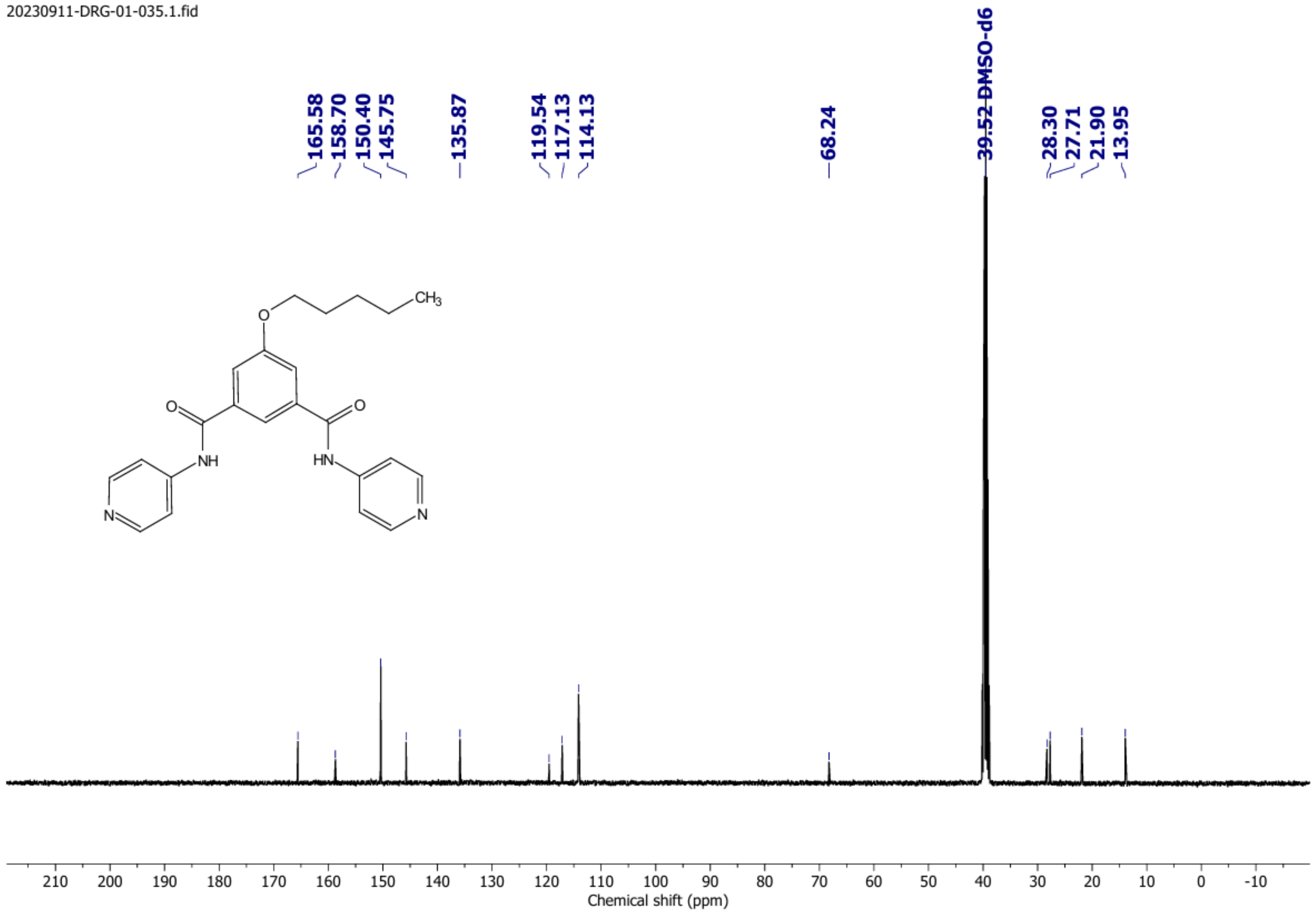

Fig. S50 ^{13}C NMR (101 MHz) spectrum of compound **1c** in DMSO-d_6.

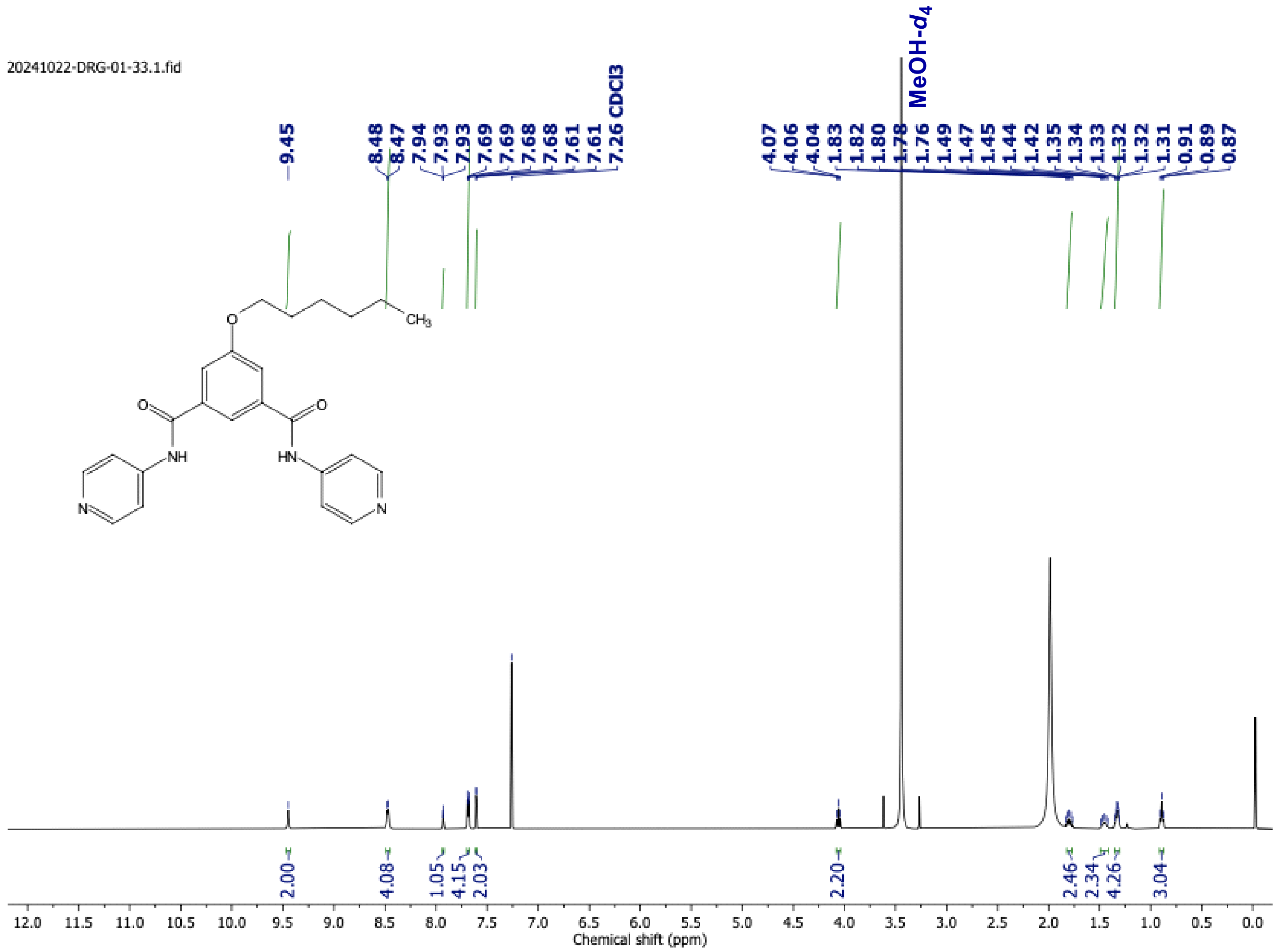

Fig. S51 ^{1}H NMR (400 MHz) spectrum of compound **1d** in 1:10 (v/v) MeOH-d_4:$CDCl_3$.

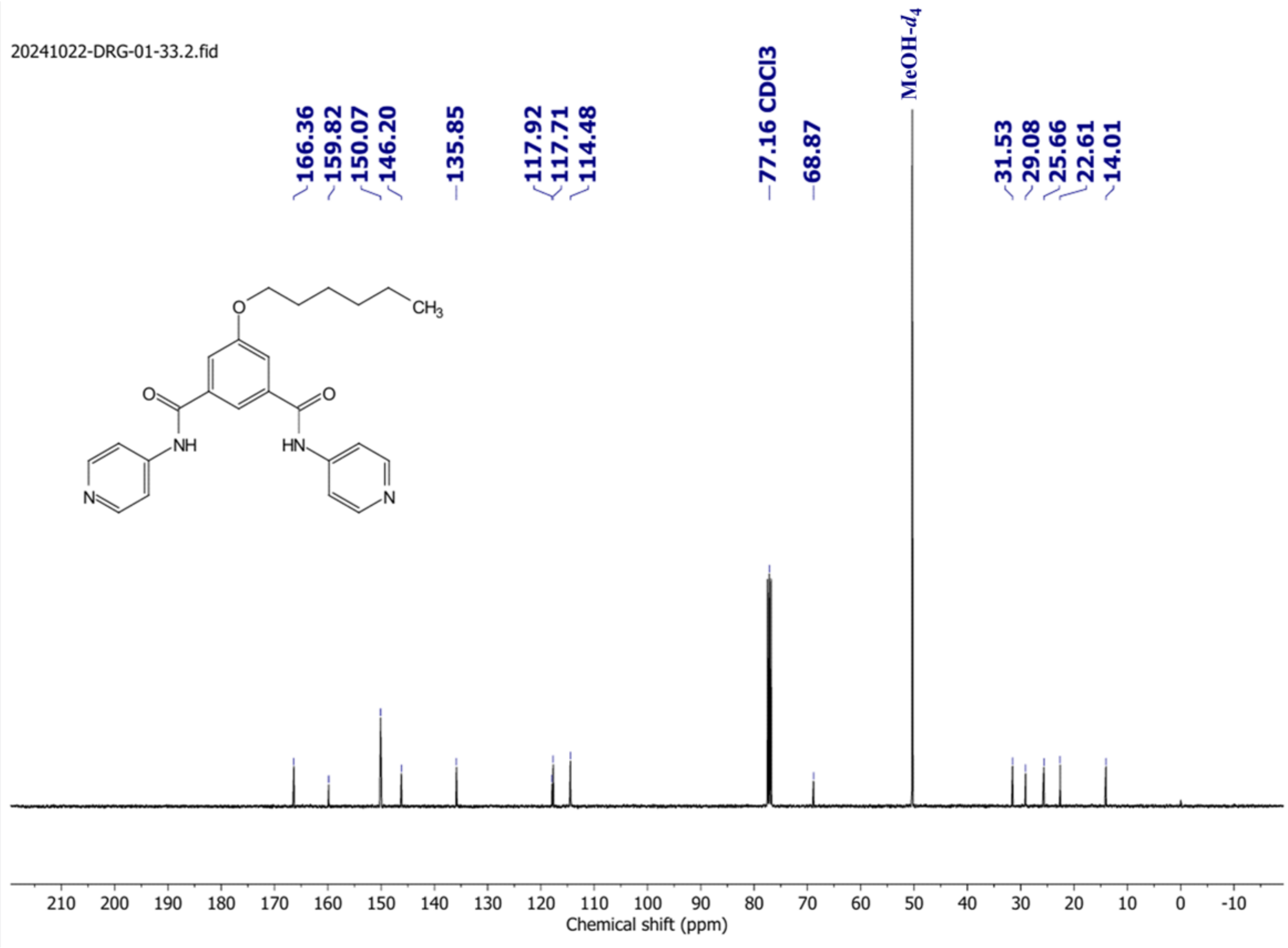

Fig. S52 ^{13}C NMR (101 MHz) spectrum of compound **1d** in 1:10 (v/v) MeOH-d_4:$CDCl_3$.

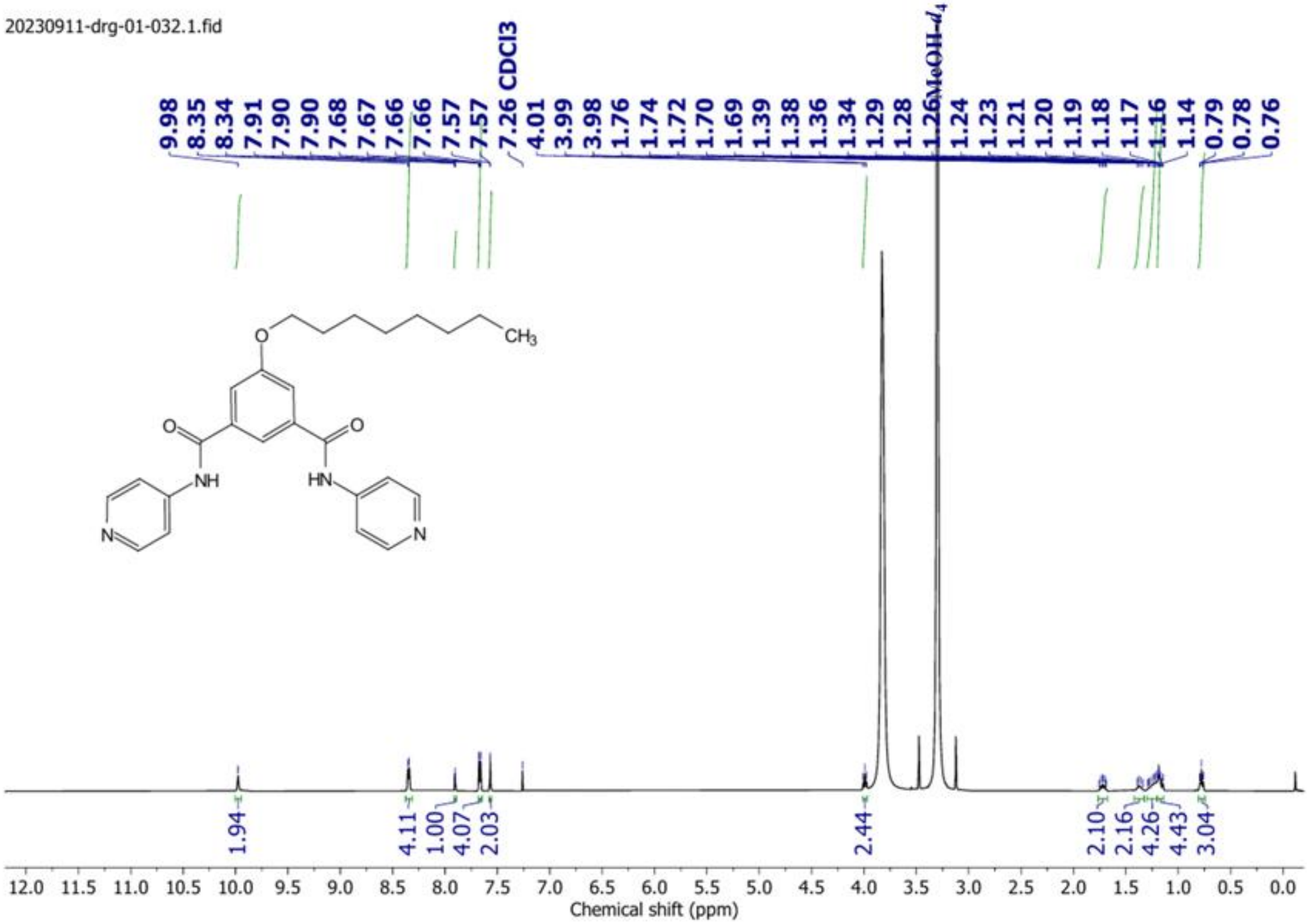

Fig. S53 ^{1}H NMR (400 MHz) spectrum of compound **1e** in 1:10 (v/v) MeOH-d_4:$CDCl_3$.

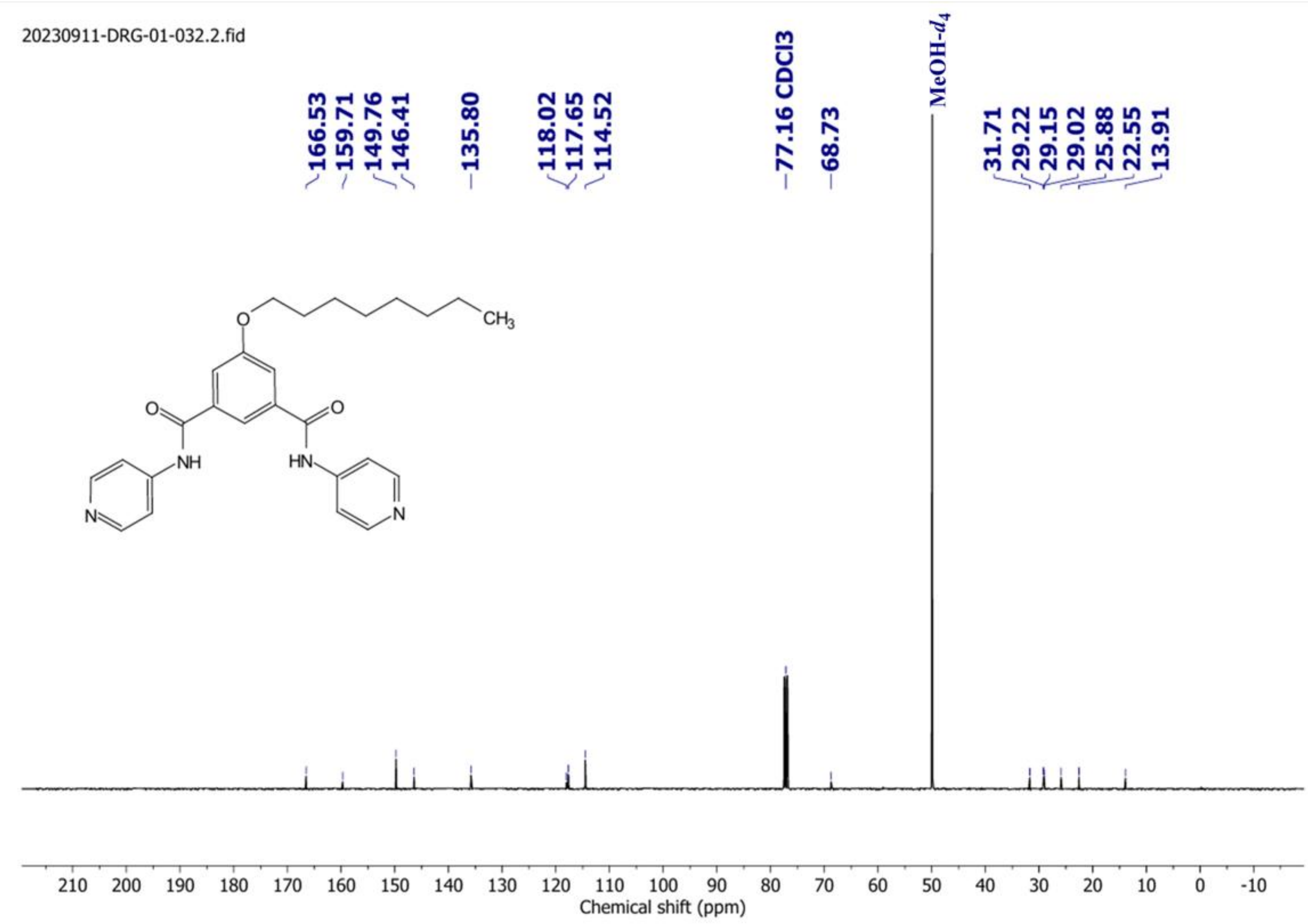

Fig. S54 ^{13}C NMR (101 MHz) spectrum of compound **1e** in 1:10 (v/v) MeOH-d_4:$CDCl_3$.

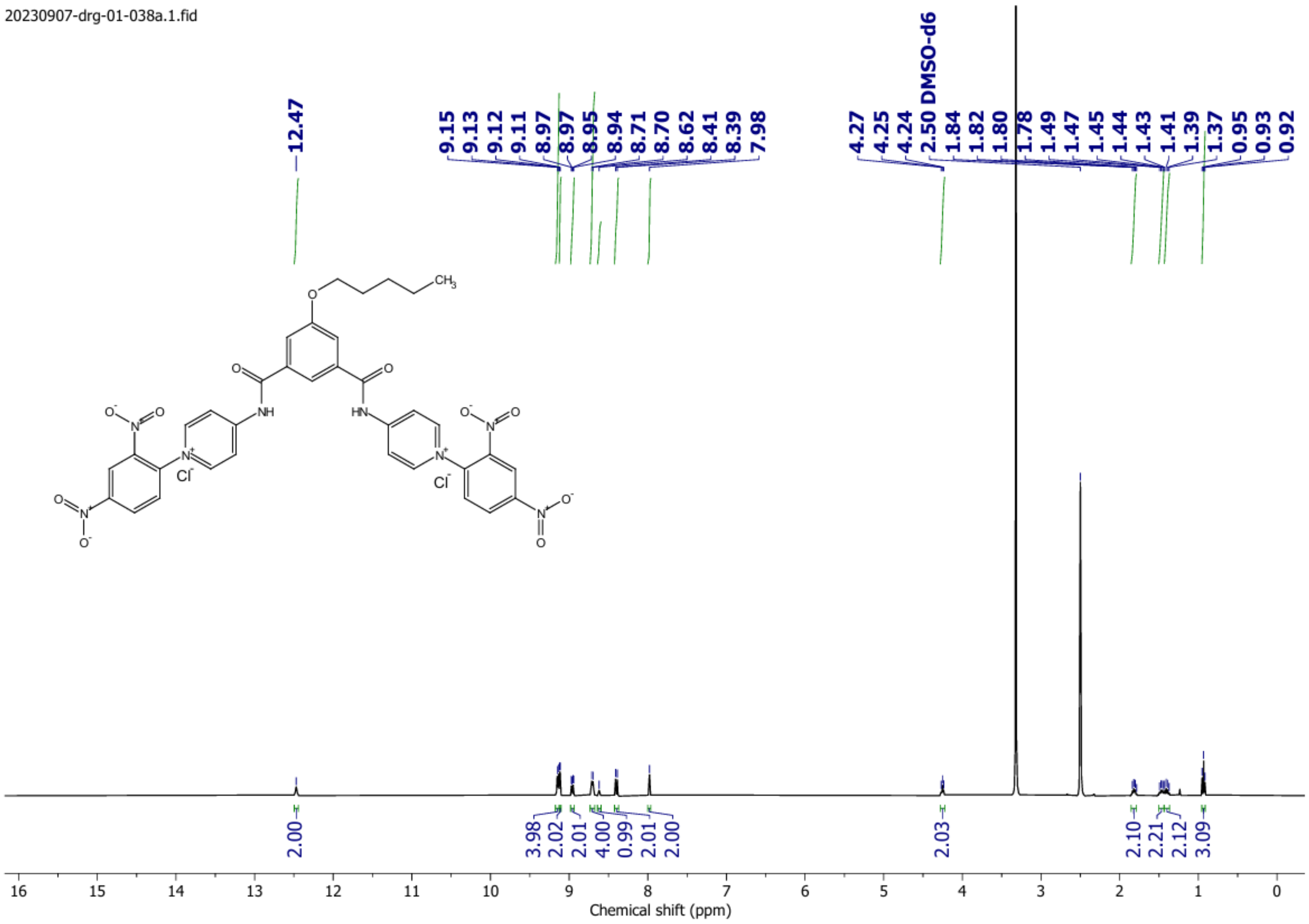

Fig. S55 ^{1}H NMR (400 MHz) spectrum of compound **1c'** in DMSO-d_6.

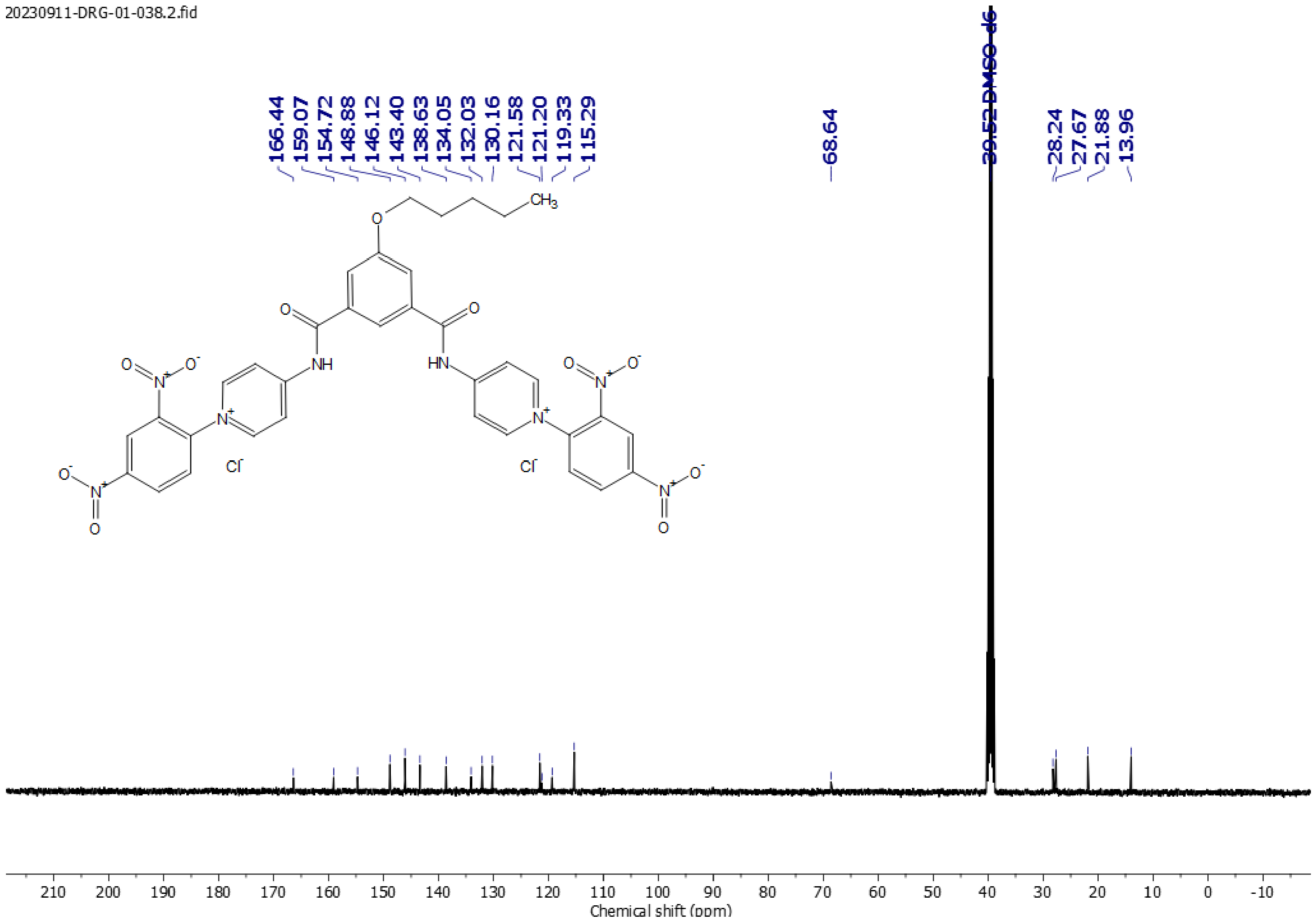

Fig. S56 ^{13}C NMR (101 MHz) spectrum of compound **1c'** in DMSO-d_6.

XV. References:

S1 Y. Yang, M. Xue, J. -F. Xiang and C. -F. Chen, *J. Am. Chem. Soc.*, 2009, **131**, 12657–12663.

S2 L. J. McCormick, S. A. Morris, A. M. Z. Slawin, S. J. Teat and R. E. Morris, *Cryst. Growth Des.*, 2016, **16**, 5771–5780.

S3 J. Hoq and E. D. Bloch, *Chem. Commun.*, 2024, **60**, 6945–6948.

S4 J. M. Granda, O. Staszewska-krajewska and J. Jurczak, *Chem. Eur. J.*, 2014, **20**, 12790–12795.

S5 M. Ahmad, S. Chattopadhayay, D. Mondal, T. Vijayakanth and P. Talukdar, *Org. Lett.*, 2021, **23**, 7319–7324.

S6 T. Saha, S. Dasari, D. Tewari, A. Prathap, K. M. Sureshan, A. K. Bera, A. Mukherjee and P. Talukdar, *J. Am. Chem. Soc.*, 2014, **136**, 14128–14135.

S7 D. Mondal, A. Sathyan, S. V. Shinde, K. K. Mishra and P. Talukdar, *Org. Biomol. Chem.*, 2018, **16**, 8690–8694.

S8 A. Mondal, S. N. Save, S. Sarkar, D. Mondal, J. Mondal, S. Sharma and P. Talukdar, *J. Am. Chem. Soc.*, 2023, **145**, 9737–9745.

S9 S. B. Salunke, J. A. Malla and P. Talukdar, *Angew. Chem., Int. Ed.*, 2019, **58**, 5354–5358.

S10 J. A. Malla, R. M. Umesh, A. Vijay, A. Mukherjee, M. Lahiri and P. Talukdar, *Chem. Sci.*, 2020, **11**, 2420–2428.

S11 S. Kar and N. Madhavan, *Chem. Eur. J.*, 2023, **29**, e202301020.

S12 H. Behera and N. Madhavan, *J. Am. Chem. Soc.*, 2017, **139**, 12919–12922.

S13 S. Chattopadhayay, K. V. Banzal and P. Talukdar, *Angew. Chem., Int. Ed.*, 2025, **64**, e202414354.

S14 M. J. Frisch, G. W. Trucks, H. B. Schlegel, G. E. Scuseria, M. A. Robb, J. R. Cheeseman, G. Scalmani, V. Barone, B. Mennucci, G. A. Petersson, H. Nakatsuji, M. Caricato, X. Li, H. P. Hratchian, A. F. Izmaylov, J. Bloino, G. Zheng, J. L. Sonnenberg, M. Hada, M. Ehara, K. Toyota, R. Fukuda, J. Hasegawa, M. Ishida, T. Nakajima, Y. Honda, O. Kitao, H. Nakai, T. Vreven, J. A. Montgomery Jr, J. E. Peralta, F. Ogliaro, M. Bearpark, J. J. Heyd, E. Brothers, K. N. Kudin, V. N. Staroverov, T. Keith, R. Kobayashi, J. Normand, K. Raghavachari, A. Rendell, J. C. Burant, S. S. Iyengar, J. Tomasi, M. Cossi, N. Rega, J. M. Millam, M. Klene, J. E. Knox, J. B. Cross, V. Bakken, C. Adamo, J. Jaramillo, R. Gomperts, R. E. Stratmann, O. Yazyev, A. J. Austin, R. Cammi, C. Pomelli, J. W. Ochterski, R. L. Martin, K. Morokuma, V. G. Zakrzewski, G. A. Voth, P. Salvador, J. J. Dannenberg, S. Dapprich, A. D. Daniels, O. Farkas, J. B. Foresman, J. V. Ortiz, J. Cioslowski and D. J. Fox, Gaussian 09, Rev. D.01, Gaussian, Inc., Wallingford, CT, 2013.

S15 J. J. P. Stewart, MOPAC2012; Stewart Computational Chemistry: Colorado Springs, CO, 2012, http://openmopac.net/.
